# Altered miRNA Signature of Developing Germ-cells in Infertile Patients Relates to the Severity of Spermatogenic Failure and Persists in Spermatozoa

**DOI:** 10.1038/srep17991

**Published:** 2015-12-09

**Authors:** Xavier Muñoz, Ana Mata, Lluís Bassas, Sara Larriba

**Affiliations:** 1 Human Molecular Genetics Group- Bellvitge Biomedical Research Institute (IDIBELL), 08908 L’Hospitalet de Llobregat, Barcelona, Spain; 2 Laboratory of Seminology and Embryology, Andrology Service-Fundació Puigvert, 08025 Barcelona, Spain

## Abstract

The aim of this study was to assess the cellular miRNA expression behaviour in testes with spermatogenic failure (SpF). We performed a high-throughput screen of 623 mature miRNAs by a quantitative RT-qPCR-based approach in histologically well-defined testicular samples with spermatogenic disruption at different germ-cell stages, which revealed altered patterns of miRNA expression. We focussed on the differentially expressed miRNAs whose expression correlated with the number of testicular mature germ-cells and described the combined expression values of a panel of three miRNAs (miR-449a, miR-34c-5p and miR-122) as a predictive test for the presence of mature germ-cells in testicular biopsy. Additionally, we determined decreased cellular miRNA content in developing germ-cells of SpF testis; this was more noticeable the earlier the stage of germ-cell differentiation was affected by maturation failure. Furthermore, we showed that the miRNA expression profile in mature sperm from mild SpF patients was widely altered. Our results suggest that the cellular miRNA content of developed germ-cells depends heavily on the efficacy of the spermatogenic process. What is more, spermatozoa that have fulfilled the differentiation process still retain the dysregulated miRNA pattern observed in the developing SpF germ-cells. This altered miRNA molecular signature may have functional implications for the male gamete.

Approximately 4% of men worldwide suffer from infertility, and in a significant proportion (∼70% of cases) it is accompanied by some degree of spermatogenic failure. Spermatogenesis is a highly orchestrated developmental process that occurs in the testicular seminiferous tubules, by which primordial germ-cells or spermatogonia develop into mature haploid spermatozoa. During the course of spermatogenesis the three major forms of cell cycle are represented: mitosis of spermatogonia; two rounds of meiosis, from primary spermatocytes to haploid round spermatids; and differentiation including structural and nuclear changes to generate mature/elongated spermatids and spermatozoa. Mature sperm are finally released into the lumen of the seminiferous tubule by a process called spermiation. At all stages of differentiation, the spermatogenic cells are in close contact with Sertoli cells which are thought to provide structural and metabolic support to the developing sperm cells. Production of sperm depends on precise, developmental stage- and germ-cell type-specific gene expression. Spermatogenesis is heavily dependent on post-transcriptional regulatory processes and miRNAs have emerged as important regulators of these events^1^,^2^,^3^.

MicroRNAs (miRNAs) are a class of endogenous small non-coding RNAs (20-23 nucleotides in length) that act as potent negative regulators of mRNA stability and translation by interacting with complementary sites on the 3′ UTR of the target mRNAs. Studies have shown that miRNAs play critical roles in a variety of biological processes such as: cell proliferation, differentiation, apoptosis and carcinogenesis^4^,^5^. MiRNAs actively participate in diverse aspects of vertebrate differentiation and development^6^,^7^,^8^,^9^, and specifically in testis differentiation in the embryo, male germline development and sperm production^3^,^10^,^11^,^12^,^13^,^14^. Accordingly, altered testicular miRNA expression has been found to accompany non-obstructive or secretory azoospermia^15^. Histological characterization was, however, not addressed in the study; therefore the authors did not consider interpreting the results in relation to the arrested maturation stage of the germline and spermatogenic phenotype.

In this study we initiated a high-throughput screen of 623 mature miRNAs using a quantitative RT-qPCR-based approach on histologically well-defined testicular samples with spermatogenic disruption at different germ-cell stages. Of the differentially expressed miRNAs in spermatogenetic deficient testes, we focussed on those whose expression correlated with the number of testicular mature germ-cells to determine their potential use as indicators of spermatogenic efficiency and/or their physiological relevance. Given the specific spatiotemporal germ-cell expression pattern of miRNAs, we also determined miRNA cellular content. Finally, in order to better understand the physiological role of these miRNAs in male infertility, the miRNA expression pattern of spermatozoa from semen was also assessed.

## Results

### Severe spermatogenic disorders show aberrant miRNA expression profiles

**Figure 1:**
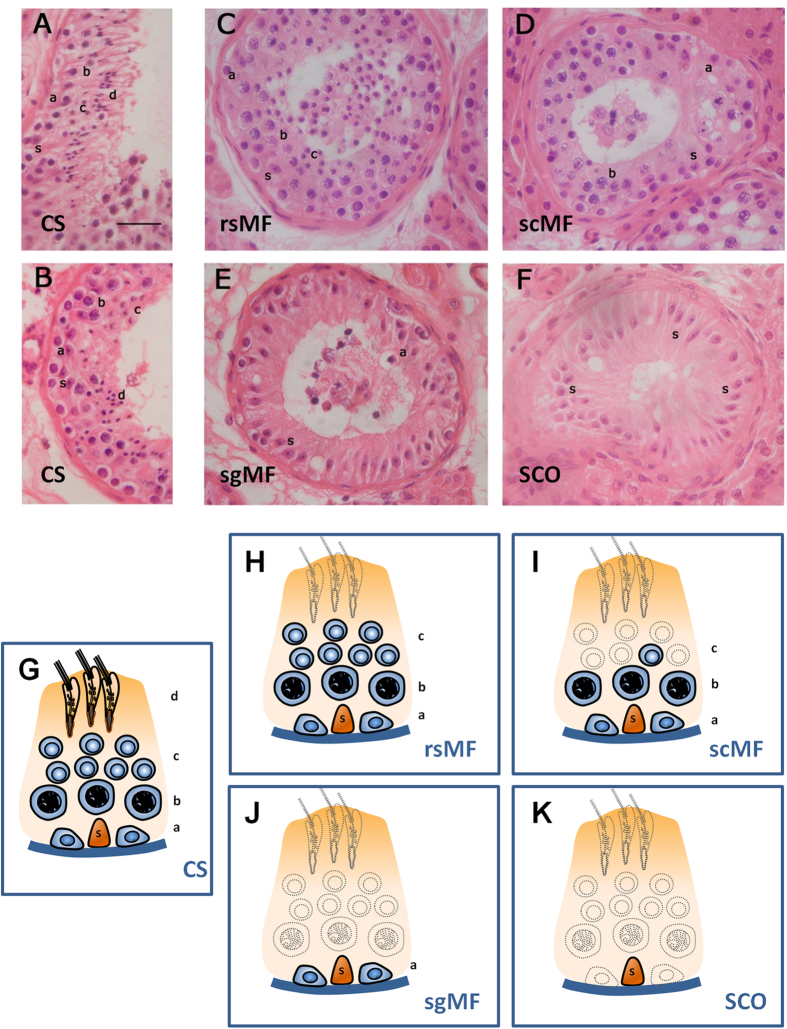
Testicular histology of representative sections of seminiferous tubules from infertile men showing different spermatogenic patterns: conserved spermatogenesis (CS) containing all germ-cell stages (**A,B**), maturation failure at the round spermatid stage (rsMF) showing no elongated spermatids (**C**), maturation failure at the spermatocyte stage (scMF) with spermatogonia and spermatocytes but few or none spermatids (**F**), maturation failure at the spermatogonia stage (sgMF) displaying only these primordial cells (**G**), and Sertoli cell-only phenotype or germ-cell aplasia (SCO) where seminiferous tubules contain exclusively Sertoli cells (**H**). Bellow, schematic diagrams, showing the epithelium of a seminiferous tubule that consists of a single Sertoli cell and the germ-cell components of the different phenotypes, are placed below the histological images: CS (**G**), rsMF (**H**), scMF (**I**), sgMF (**J**), and SCO (**K**). Absent cell populations are drawn as dashed line shadings. Examples of different cell types, including Sertoli cells and specific germ-cell stages, are identified with lower case letters: (**a**): spermatogonia; (**b**): primary spermatocytes; (**c**): round spermatids; (**d**): elongated spermatids; (**s**): Sertoli cell nucleus. Hematoxilin and eosin staining. Bar = 50μm.

In order to identify global testicular miRNA changes associated with severe SpF, we first analysed the level of expression of 623 human miRNAs in testes with different histological phenotypes: conserved spermatogenesis, showing all the stages of spermatogenesis (CS n = 3; Table 1 no. T1-T3; Fig. 1A,B,G); maturation failure at the spermatocyte stage, showing spermatogonia and spermatocytes but rarely spermatids (SpF-scMF n = 3; Table 1 no. T20-T22; Fig 1D,I); and germ-cell aplasia or Sertoli cell-only syndrome, where the seminiferous tubules contained exclusively Sertoli cells (SCO n = 3: Table 1 no. T36-T38; Fig 1F,K). CS and SpF-scMF samples show similar numbers of spermatogonia and spermatocytes in the tubule. Round and elongated spermatids were nearly absent in the SpF group although some mature germ-cells were still present in one of the SpF-scMF samples (Fig. 1, Table 1).

No amplification values were obtained for 90 miRNAs suggesting that the transcript levels were beneath the detection threshold of the technique. Of the amplified miRNAs, 98 were excluded for further analysis due to poor amplification efficiency across samples (missing expression values for 67%). The remaining miRNAs (n = 435) were further statistically evaluated and the results are presented in Supplementary Table S1.

The presence of 421 miRNAs (Cp value <38) was confirmed in the CS samples (63 of them were not expressed in SCO and 7 were not expressed in either SCO or SpF-scMF, suggesting that they are preferentially expressed in the germline and in the postmeiotic germ-cells, respectively), whereas 15 miRNAs were detected in SCO but not in CS samples (Supplementary Table S1) which suggests that they are expressed in somatic cells. We found 186 miRNAs in SCO and/or scMF groups that presented significant differences in expression when compared with CS controls. In detail, 110 miRNAs were significantly over-expressed (fold-change increase range: 1,30-9,77; fold-change increase >2 n = 67) and 72 were under-expressed (fold-change decrease range: 1,44-9000; fold-change decrease >2 n = 67) in the SCO-CS comparison (Supplementary Table S1). From the statistically significant down-regulated miRNAs, 50 miRNAs were absent in SCO; Cp value >38 (Supplementary Table S1). Among the miRNAs presenting the greatest fold-change decrease (>7Cp) we identified: miR-122, miR-31, miR-34b, miR-34c-5p, miR-375, miR-449a, miR-449b, miR-449b*, miR-515-5p, miR-517b, miR-517c, miR-518e, miR-518e*, miR-518f, miR-519a, miR-519b-3p, miR-519d, miR-520b, miR-520c-3p, miR-520f, miR-520g, miR-520h and miR-526b*. The miRNA miR-449a presented the greatest fold-change reduction in expression in the SCO phenotype. Some of the down-regulated miRNAs are located in miRNA clusters on chromosome 19. One is the miR-371,2,3 cluster (miR-371-3p, miR-373, miR-373* were down-regulated in SCO patients) and the other is the non-conserved cluster^16^ that is comprised of 54 miRNAs (42 of which were absent in SCO patients; Supplementary table S1).

In the SpF-scMF samples, 5 miRNAs were found to be up-regulated (fold-change increase range: 1,45-51,40; fold-change increase >2 n = 4) and 16 miRNAs down-regulated (fold-change decrease range: 1,34-46,08; fold-change decrease >2 n = 12) when compared with CS (Supplementary Table S1).

Seventeen of the differentially expressed miRNAs were shared among SCO and SpF-scMF groups, either down- (n = 14) or up-regulated (n = 3), when compared with CS (Supplementary Table S1). Remarkably, among the shared down-regulated miRNAs, there was no expression of miR-511, miR-521 and miR-885-5p in either of the infertile phenotypes, suggesting that they were postmeiotic miRNAs and that the absence of expression might be at least partially due to the loss of round and elongated spermatids in the testis. However, this was not the case for miR-122, miR-182, miR-34b, miR-512-5p, miR-518f and miR-519c-3p, which all presented a null expression in SCO but a moderate reduction of expression in samples with meiotic arrest, suggesting they are expressed along the germline, although preferentially at the postmeiotic level. Furthermore, the presence of shared up-regulated miRNAs (miR-10b*, miR-202*, miR-210) is probably attributable to the reduction of germ-cell number in the tubule and suggests a somatic contribution to their expression; the loss of germ-cells enriches the relative contribution of the remaining testicular somatic cells (Supplementary Table S1).

### The expression of testicular miRNAs correlates with the presence of intratubular mature germ-cells

In order to assess whether there is an association between miRNA expression and histological parameters and confirm whether the results could be of physiological relevance and/or have potential implications for diagnosis, we performed a Pearson’s correlation analysis between molecular and histological variants of the nine samples used for the testicular miRNA profiling study (Supplementary Table S2). Strikingly, TESE value and the number of elongated spermatids per tubule (quantified by the histological analysis) were both highly significantly correlated with 68 miRNAs: 51 miRNAs presented positive Pearson’s correlation coefficients (PCC): r: 0,999-0,672 and 17 miRNAs negative PCC; r: −0,955- −0,696 (Table 2, Supplementary Table S2). The TESE value is defined as the number of spermatozoa that was obtained directly after processing 100 mg of biopsy in 1 mL of medium, and it is highly and positively correlated with the number of intra-testicular mature germ cells (r: 0,993; p < 0,0001). We hypothesize that there may be a threshold level of miRNA expression related to the presence of intra-testicular sperm. For this purpose we selected TESE value >0,002 as the state variable. Interestingly, 28 miRNAs (depicted in bold and italics in Table 2; 22 of them were found to be positively correlated) resulted in good predictive accuracy (AUC > 0,900 for those negatively correlated and AUC < 0,05 for those positively correlated; *p* ≤ 0,03) after the ROC curve analysis, suggesting they have a potential use as indicators of the presence of spermatozoa in the testis.

**Figure 2:**
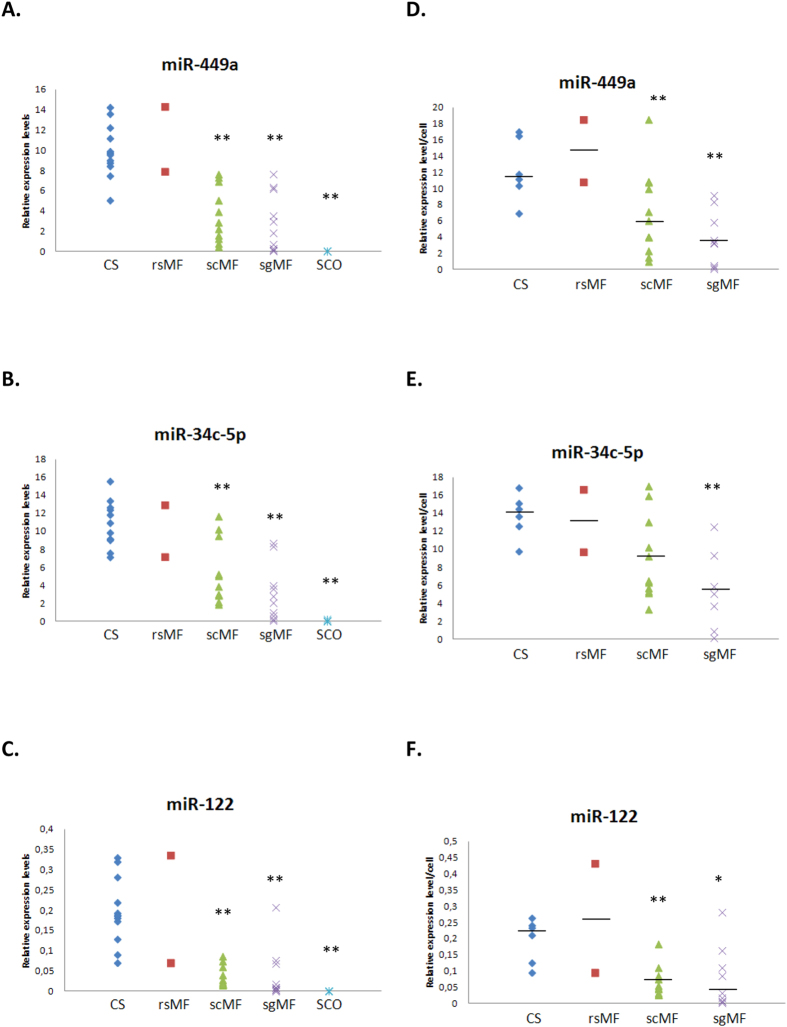
Tissue expression profiling of selected miRNAs: miR-449a (A), miR-34c-5p (**B**) and miR-122 (C) by qPCR in testes with conserved spermatogenesis (CS), maturation failure at the round spermatid (rsMF), the spermatocyte (scMF) and the spermatogonia (sgMF) stages and Sertoli cell-only syndrome (SCO). Normalized expression levels relative to miR-30e-3p are shown. Expression per cell profiling of miR-449a (**D**), miR-34c-5p (**E**) and miR-122 (**F**) displayed as expression ratio per spermatocyte/round spermatid (×100) in testes with conserved spermatogenesis (CS), maturation failure at the round spermatid (rsMF), the spermatocyte (scMF) and the spermatogonia (sgMF) stages (see materials and methods for explanation). The horizontal bar displays the median cellular expression level. Significant differences from the control are indicated: *p < 0,05, **p < 0,01 (Mann-Whitney U test).

In order to validate these data we verified our results for several miRNAs in a larger cohort of SpF testicular samples. We focused our attention on miR-449a, miR-34c-5p and miR-122 that were found to be down-regulated in SpF-scMF and presented the greatest fold-change expression decrease (>7Cp) in SCO as stated above. In detail, five SCO, twenty-three SpF, as well as twelve CS testicular samples (Table 1), were analysed. We found statistically significant differences in miRNA expression levels between CS and SpF samples; this was more remarkable as the maturation failure affects an earlier germline stage showing a hierarchy of values (CS = rsMF > scMF > sgMF > SCO; Fig. 2A–C). Remarkably, miRNA expression levels were significantly and positively correlated with the number of elongated spermatids, as expected (PCC r = 0,654, *p* < 0,0001 for miR-449a; r = 0,680, *p* < 0,0001 for miR-34c-5p; r = 0,449, *p* = 0,011 for miR-122), and consequently with TESE value (PCC r = 0,674, *p* < 0,0001 for miR-449a; r = 0,648, *p* < 0,0001 for miR-34c-5p; r = 0,698, *p* < 0,0001 for miR-122). When TESE value >0,002 was selected as the state variable, all three independently presented AUC >0,90 (*p* < 0,0001). Combined miRNA expression values could also identify the presence of spermatozoa in testicular samples. The sensitivity and the specificity for predicting samples with TESE value >0,002 were 83,3% and 88,5%, respectively, confirming that their expression values could be useful to predict the chance of finding spermatozoa in the testicular biopsy.

### Cellular expression levels of germ-cell–specific miRNAs

To have a better insight into miRNA level related to the testicular sample cell composition and to determine whether cellular miRNA expression is altered in SpF, germ-cell specific-miRNA testicular expression data of the twenty-three SpF individuals were additionally adjusted for the proportion of expressing germ-cell stages per seminiferous tubule for each respective sample (see materials and methods section). The miR-34b/c and miR-449 clusters are preferentially expressed in testicular tissue and localized to spermatocytes and spermatids^12^,^17^, and miR-122 is enriched in late-stage male germ-cells^18^. Therefore, expression data for miR-449a, miR-34c-5p and miR-122 were divided by the proportion of spermatocytes + round spermatids per tubule (x100) obtaining a decreased miRNA cellular expression profile in SpF; this was more noticeable the earlier the stage of germline was affected by maturation failure, as happens in the sgMF phenotype (Fig. 2D–F), which suggests that cellular miRNA content depends heavily on the efficacy of the earliest stages of the spermatogenic process.

**Figure 3:**
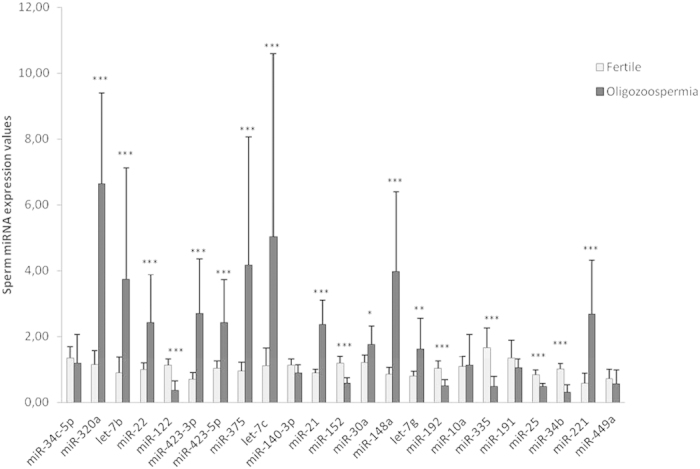
Sperm expression values of selected miRNAs determined by qPCR from fertile (white bars) and oligozoospermic (black bars) semen samples. Normalized expression levels relative to the mean of the twenty-three assays are shown. Significant differences from the control are indicated: *p < 0,05; **p < 0,01; ***p ≤ 0,005 (Mann-Whitney U test).

Furthermore, the expression values of miR-449a, miR-34c-5p, miR-34b and miR-122 were also determined in semen spermatozoa from seven fertile and nine oligozoospermic individuals (with mild impairment of germ-cell maturation process) (Table 3). Reduced levels of all four miRNAs were observed, although the difference was only statistically significant (*p* < 0,0001) for miR-34b (fold-change decrease: 3,22) and miR-122 (fold-change decrease: 3,12), in sperm from oligozoospermic patients compared with controls (Fig. 3). Interestingly, miR-34b and miR-122 expression levels were highly positively and significantly (*p* = 0,001) correlated with the number of spermatozoa in semen (PCC r = 0,765, for miR-122; r = 0,730, for miR-34b). Altogether this analysis suggests that the changes in the SpF testicular miRNA expression patterns cannot be exclusively explained by the proportion of germ-cells in the testicular sample but rather that an altered miRNA expression profile of individual germ-cells is also being observed. Furthermore, changes in developing germ-cell miRNA expression patterns remain in the latest maturation stage of the germline with potential functional implications for the cell.

Additional changes in miRNA expression were observed in spermatozoa from oligozoospermic semen. In total from the 23 miRNAs analysed, 12 miRNAs were significantly more abundant: let-7b, let-7c, let-7g, miR-21, miR-22, miR-30a, miR-148a, miR-221, miR-320a, miR-375, miR-423-3p, miR-423-5p [all but miR-30a presenting an increase of more than 50%], and 6 were significantly less abundant: miR-25, miR-34b, miR-122, miR-152, miR-192, miR-335, compared with normozoospermic fertile controls (Fig. 3).

## Discussion

Several threads of evidence suggest that miRNAs could play an active and important role during spermatogenesis. The miRNA expression profiles of various testicular cell populations^14^,^3^,^18^,^19^,^20^,^21^, as well as their specific downstream effects, have been previously described. Furthermore, Dicer1, a protein necessary for miRNA processing and Dnd1, a protein implicated in the protection of mRNAs from miRNAs, are essential for the completion of spermatogenesis^3^,^11^,^22^. We have previously demonstrated that low spermatogenic efficiency in infertile men is accompanied by an altered gene expression capacity of germ-cells, which contributes to unsuccessful sperm production^23^,^24^, thus, in this context, it is reasonable to study the cellular miRNA expression behavior, as a likely mechanism of gene expression regulation, in spermatogenic disorders.

Our study shows aberrant testicular miRNA expression patterns associated with severe SpF. However, the diversity of cellular composition of the testis is an inherent characteristic of the tissue, which should be taken into account when studying expression profiles in this organ. Several miRNAs are commonly expressed among different cell types, but some miRNAs are specific to either somatic- or germ-cells. Furthermore, similar to protein coding mRNAs, miRNAs follow phase-specific expression patterns inside the testis. Since the pathological seminiferous tubules lack germ-cells to varying degrees, changes in gene expression at the tissue level can reflect specific changes in the cellular miRNA content as well as changes in the cell type composition in pathological testis.

Our data first indicate that SCO specimens exhibited a highly altered miRNA expression profile compared with CS and SpF samples; this must mainly be reflecting the differences in cell type composition, as the germline is completely absent in SCO testes. Therefore, a substantial contribution by somatic cells to testicular expression was inferred for the 110 miRNAs up-regulated in SCO. Phenotypic changes such as a greater number of Sertoli cells in SCO and/or an increase in miRNA levels in somatic cells could be an additional contributing factor. Five of these miRNAs were also up-regulated in SpF-scMF samples (miR-10b*, the Sertoli cell expressed miR-202*^10^, miR-210, miR-504, miR-551a) suggesting that an increase in somatic cell content of these miRNAs accompanies and/or contributes to a meiotic blockade. Some of these miRNAs, such as miR-10b*^25^, have been described to be involved in cell cycle regulation^25^.

Additionally, the absence of expression of 70 miRNAs in SCO samples seems to suggest that they are mainly expressed in germ-cells. Only fourteen of these under-expressed miRNAs are shared with SpF samples suggesting that most of the SCO down-regulated miRNA are expressed all along the germline.

Interestingly, some SCO down-regulated miRNAs were located on chromosome 19, mapping to the non-conserved cluster comprised of 54 miRNAs^16^ and cluster miR-371-3 (both described as oncogenes in testicular germ-cell tumours^26^). Two additional miRNA clusters, miR-34b/c and miR-449 (consisting of three members: miR-449a, miR-449b, and miR-449c) presented the greatest fold-change reduction in expression in SCO testis and three of the miRNAs contained in these clusters, miR-34b, miR-34b*, miR-449a, were also differentially reduced in the SpF-scMF phenotype. In a previous study, microarray analysis also showed that these miRNAs were down-regulated in formalin-fixed paraffin-embedded (FFPE) testicular samples from azoospermic patients^27^, supporting the veracity of these results. Strikingly, a decreased expression level of miR-34c-5p was also found in seminal plasma from histologically uncharacterized men with azoospermia^28^, this is probably related to the absence of the germline in the testis, corroborating the testicular results. There is strong evidence that the miR-34b/c and miR-449 clusters are involved in spermatogenesis. They have been reported to be postmeiotically expressed in the testis and sperm, but absent in oocytes^12^,^17^ and the expression of both miRNA clusters are essential for normal spermiogenesis^29^. Both miRNA clusters have been described as functioning redundantly by targeting the E2F-pRb pathway^17^. *TGIF2* and *NOTCH2* are targets of miR-34c^12^ and NOTCH1, regulated by both miR-34b and miR-34c, is critical for germ-cell differentiation and survival during spermatogenesis. It is worth noting that deletion of both, miR-34b/c and miR-449, loci in mice impairs both meiosis and the final stages of spermatozoa maturation, but does not lead to a SCO phenotype^30^. These findings together with our results suggest that, a widely altered miRNA molecular signature in germ-cells, but not individual miRNAs, is associated with severe spermatogenic disorders.

Although the expression of several miRNAs was found to be altered in frozen samples (our study) and FFPE testicular samples^27^ with severe spermatogenic impairment, there were specific individual miRNAs that were poorly correlated between both studies. Specifically, we found no difference in expression for miR-17-5p, miR-18a, miR-19a, miR-20a, miR-19b-1, located in the testicular-expressed miR-17-92 cluster on chromosome 13, previously found to be associated with severe defects in sperm production by miRNA microarray analysis of FFPE samples^27^. Our results suggest that the expression of this miRNA cluster is not exclusive to germ-cells. Strong expression of miR-17-5p has been identified in CIS testicular cells and pachytene meiotic and postmeiotic germ-cells^31^, but a testis-specific expression pattern was not shown, which is in agreement with our results. Differences in results may arise from the use of different strategies of miRNA quantification in both studies. The quantitative real-time qPCR-based strategy is reported to have a better specificity and sensitivity^32^,^33^ compared with microarray-based analysis; this could explain the differences in the testicular miRNA profile between the microarray study with FFPE samples^27^ and our study based on RT-qPCR with frozen testicular tissue.

Interestingly, a positive correlation with a strong predictive accuracy (AUC > 0,900) between the individual expression values of 22 testicular miRNAs, and the presence of intra-testicular spermatozoa was determined for the first time. What is more, the sensitivity and specificity for predicting samples with TESE value > 0,002 increase notably when the expression values of three miRNAs (miR-449a, miR-34c-5p and miR-122) are combined. Therefore, the use of miRNA expression values have potential implications for diagnosis of infertility as a means of predicting the availability of sperm in the biopsy for assisted reproduction treatment, especially for those individuals who are considering further attempts of invasive testicular extraction after a first negative biopsy with fine needle sperm aspiration. This correlation could be partially attributed to the proportion of germ-cells in the testis that specifically express those miRNAs, however, a statistically significant decrease of cellular expression of germ-specific miRNAs was also observed suggesting that miRNA levels in germ-cells from SpF individuals are additionally modified. What is more, this change in miRNA expression levels, at least those affecting the germ-cell expressed miR-34b and miR-122, might not only reflect and/or contribute to an unsuccessful germ-cell development, but could also potentially affect the fertilization potential of male gametes, as miRNA levels remain altered in mature spermatozoa from oligozoospermic patients. Interestingly, miR-122 and miR-34b miRNAs were also found to be differentially expressed in previous studies between sperm from normospermic and oligoasthenozoospermic subfertile men^34^. Furthermore, miR-122, miR-34c-5p and miR-34b were previously described as potential sperm biomarkers in semen for the diagnosis of male infertility to discriminate individuals with obstructive oligozoospermia from men with non-obstructive subfertility^35^.

Our results additionally provide evidence that the profile of miRNA expression in semen spermatozoa from mild SpF patients is widely altered: eighteen out of the twenty-three miRNAs tested presented changes in their expression levels. Most of the differentially expressed miRNAs in spermatozoa from oligozoospermic men in our study have not been previously described. However, although some of these miRNA changes are attributable to early events in spermatogenesis as we described for miR-34b and miR-122, it remains unclear whether other miRNA altered expression pattern represent the consequence of and/or contribute to spermatogenic defects. For example, several up-regulated miRNAs have been described as being associated with senescence. MiR-22 and miR-30a-5p over-expression suppresses growth and induces acquisition of a senescent phenotype in normal and cancerous human cells^36^. Curiously 10 of the 12 miRNAs found up-regulated in oligozoospermic sperm were also up-regulated in SCO, suggesting that an increase of the cellular levels of these miRNAs took place in both somatic- and germ-cells in spermatogenic disorders. Furthermore, the differences in miRNA expression found in semen spermatozoa from oligozoospermic men suggest that the germ-cells that manage to complete the differentiation process in the SpF testis display a similar altered miRNA molecular signature to the one found in earlier germ-cell stages and this may define the fertilizing capacity of these cells.

In summary, our study provides evidence that testicular somatic- and germ-cells in spermatogenic disorders exhibit associated patterns of miRNA expression deregulation, and that these are more severe the earlier the stage of germ-cell differentiation is affected by maturation failure. This in turn contributes to spermatogenic blockade. We therefore propose that the miRNA signature in germ-cells not only plays a role in the spermatogenic process but is also an indicator of its likelihood of success. Our findings further suggest that the cellular miRNA content of mature germ-cells depends heavily on the efficacy of the spermatogenic process; this fact further determines the miRNA molecular signature of spermatozoa that have fulfilled the differentiation process in SpF testes, with potential functional implications for the gamete. Such information provides a mechanistic molecular insight into the processes that regulate and control fertility.

## Material and Methods

### Subjects of study

Patients and controls participating in the study were selected from men referred to the Andrology Service of the Fundació Puigvert. The study was approved by the Institutional Review Board of the Centre and all the participants signed a written informed consent. The methods were carried out in accordance with the approved guidelines.

For the molecular analysis involving testicular samples, our study recruited twenty-three infertile patients showing spermatogenic failure (SpF) at different germ-cell stages, with a phenotype consistent with non-obstructive/secretory azoospermia or severe oligozoospermia (<5 million sperm/mL). In addition, five patients with complete Sertoli cell-only syndrome (SCO) or germ-cell aplasia, were studied as controls of somatic cells (negative controls) and twelve individuals who were diagnosed with obstructive azoospermia and showed conserved spermatogenesis (CS) were studied as positive controls (Fig. 1, Table 1).

Semen samples were additionally obtained from 7 normozoospermic fertile individuals consulting for vasectomy (control group) and 9 infertile men diagnosed with oligozoospermia (sperm concentration range 4–13 millions/mL) (patient group) (Table 3).

The routine clinical and genetic procedures for infertile patients included medical history, physical examination, semen analyses [performed in accordance with World Health Organization guidelines^37^, hormonal study, karyotype and analysis of Y-chromosome microdeletions. Men with spermatogenic disorders included in the study did not show clinical factors (varicocele, infection, immunologic factors, anatomic malformation, or chemical insults) or genetic causes (chromosomal aberration or a Y-chromosome microdeletion) for their infertility.

### Histological analysis of testicular samples

Testicular biopsies from infertile men were obtained when necessary to confirm the clinical diagnosis and for sperm retrieval (TESE) and cryopreservation purposes. Each specimen was divided into three aliquots, one piece (≈10–20 mg) was fixed in Bouin’s solution and reserved for histological analysis, a second aliquot (≈100–200 mg) processed for sperm extraction and the third (≈10 mg) was immediately transferred to liquid nitrogen and stored at –80 °C until analysis for gene expression experiments.

After hematoxylin-eosin staining of paraffin samples (5-μm sections), patients were first selected on the basis of having a homogeneous histological pattern of >20 tubules from the same testicular section. An assessment of spermatogenic status and of the severity of the alteration was performed by quantification of specific germ-cells (spermatogonia, spermatocytes I, round spermatids and elongated spermatids) and Sertoli cells. The average number per tubule was calculated after analysis of at least 15–20 cross-sectioned tubules per testis. A modified Johnsen score (JS) count^38^ was calculated on the basis of the number of different cell types quantified per tubule.

Using this strategy we confirmed the diagnosis of SCO and CS phenotypes. With respect to SpF patients, two of them presented maturation failure at the round spermatid level (SpF-rsMF), eleven at the spermatocyte level (SpF-scMF) and ten at the spermatogonia level (SpF-sgMF), defined by a diminished number of cells at this specific stage and the subsequent germ-cell stages in their tubules compared with CS samples (Table 1, Fig. 1).

### Selection of spermatozoa

Semen samples were processed by the method of discontinuous density gradient centrifugation (PureSperm®, Nidacon International AB) in order to remove dead sperm, seminal plasma, debris and somatic cells and thus, enrich for motile spermatozoa.

### Small RNA-containing total RNA isolation

RNA was obtained from the testicular biopsy using the miRVANA miRNA isolation kit (Ambion®-Life Technologies), following the manufacturer’s instructions, and the integrity of RNA was assessed using the 2100 Bioanalyzer (Agilent Technologies). All RNA samples from testicular tissue presented a RNA integrity number (RIN) value >7,5 and OD 260/280 nm ratio ≥1,85.

Additionally, the pool of the gradient-purified spermatozoa was processed to obtain total RNA using the miRCURY RNA Isolation Kit-Cell and Plant (Exiqon), according to the instructions provided by the manufacturer with minor modifications. Briefly, lysis buffer was added to the samples at 350 μl /5 × 10^6^ cells. The lysates were homogenized with a 20-gauge needle and heated for 30 min at 60°C. The process then continued with step 3 of the kit.

Both, testicular and sperm, RNA samples were treated with DNAse (DNA-free kit, Ambion®-Life Technologies) to prevent genomic DNA interference.

### Testicular miRNA qPCR profiling

For the miRNA screening, each RNA sample was analyzed in three replicate RT reactions. 40 ng RNA was reverse transcribed in 40 μl reactions using the miRCURY LNA™ Universal RT microRNA PCR, Polyadenylation and cDNA synthesis kit (Exiqon). cDNA was diluted 100x and assayed in 10 μl PCR reactions according to the protocol for miRCURY LNA™ Universal RT microRNA PCR; each miRNA was assayed once by qPCR on the microRNA Ready-to-Use PCR, Human panels I and II that include 623 mature miRNAs of miRBase (*www.**mirbase**.org*/). Negative controls excluding the template from the reverse transcription reaction were performed and profiled like the samples. cDNA and SYBR Green mastermix were transferred to qPCR panels preloaded with primers, using a pipetting robot. The amplification was performed in a LightCycler® 480 Real-Time PCR System (Roche) in 384 well plates. The amplification curves were analyzed using the Roche LC software, both for determination of Crossing Points-Cp (by the 2nd derivative method) and for melting curve analysis. The amplification efficiency was calculated using algorithms similar to the LinReg software. All assays were inspected for distinct melting curves and the Tm was checked to be within known specifications for the assay. Experiments were conducted at Exiqon Services.

Next, to correct for potential overall differences between the samples, for each sample, raw data (Cp values) were normalized to the mean of the 50 most stable assays (mean 50) that were detected in all samples: dCp = mean 50 Cp – assay Cp. Those assays were previously selected as being the ones that presented the lowest coefficient of variation (CV < 0,018) of Cp values among samples in the study (Supplementary Table S3) as well as no statistical differences in absolute expression levels between groups, either individually or the mean value.

The relative quantitative method of 2^dCp^ was used to calculate the relative quantification (RQ) miRNA expression values.

### Quantification of selected miRNAs by RT-qPCR analysis

MiRNA-specific first-stranded cDNA was obtained by reverse transcription (RT) of 20 ng (for testicular samples) and 10 ng (for spermatozoa) of RNA in 10 μl, using the Universal cDNA synthesis kit (Exiqon). Two independent RT reactions were performed for each sperm sample. The resulting cDNA solution was stored at −20 °C.

For quantitative real-time PCR (qPCR) analysis cDNA was diluted (80× for cDNA testicular samples; 12,5× for spermatozoa) and assayed in 10 μl PCR reactions according to the protocol for miRCURY LNA™ Universal RT microRNA PCR (Exiqon). For testicular samples, triplicate amplification reactions of individual assays (Supplementary Table S4) were carried out on a LC96 (Lightcycler® 96 Real-time PCR Instrument; Roche) whereas qPCR experiments of spermatozoa samples were performed in duplicate from each of the two independent RT reactions on the Pick and Mix microRNA PCR Panel (Exiqon) in a LC480 (Roche). The twenty-three miRNAs tested for spermatozoa experiments (Supplementary Table S4) were selected from those identified in fertile spermatozoa^39^.

Target miRNA expression for testicular samples was calculated relative to the expression of miR-30e-3p, chosen from the testicular miRNA qPCR profiling study as one of the 50 most stable miRNAs (Supplementary Table S3). For normalization of data for sperm samples, we applied the mean of the twenty-three assays that were detected in all samples. The RQ values were calculated using the 2^dCp^ strategy.

To determine the cellular miRNA content of testicular germ-cells, we considered the fact that the miRNA expression levels per tubule are the result of a composite of different cell types, as we have previously described for determining mRNA germ-cell content^23^,^24^. Therefore, we calculated the miRNA expression levels per germ-cell by adjusting total expression levels to the proportion of germ-cell stages that specifically express the miRNAs in a seminiferous tubule, to correct for the actual amount of expressing germ-cells in each particular sample.

### Statistical analysis

The non-parametric Kruskal–Wallis test was used to analyze the differences in clinical data and absolute expression levels of reference genes. Unpaired two-tailed t test was used to analyze the differences in relative expression of testicular miRNAs in each patient group compared with controls in the miRNA profiling study. The non-parametric Mann–Whitney U-test was used to evaluate differences in relative expression in testes and sperm of selected miRNAs in each patient group compared with controls. Pearson product-moment correlation coefficients (PCC) were calculated to determine the correlation between the miRNAs RQ values and the various histological parameters in patient groups and controls. Receiver operating characteristic (ROC) curve analysis of the relative expression values was used to distinguish those individuals with TESE value >0,002, which was selected as the state variable. Accuracy was measured as the area under the ROC curve (AUC). A *p*-value <0,05 was considered significant.

## Additional Information

**How to cite this article**: Muñoz, X. *et al*. Altered miRNA Signature of Developing Germ-cells in Infertile Patients Relates to the Severity of Spermatogenic Failure and Persists in Spermatozoa. *Sci. Rep*. **5**, 17991; doi: 10.1038/srep17991 (2015).

## Supplementary Material

Supplementary Information

## Figures and Tables

**Table 1:** Phenotypical and histological description of the testicular samples included in the study.

Patient No.	Diagnosis	Histology	TESE value (million/mL)	Semen sperm conc (million/mL)	Spgonia (n)^(a)^	Spcytes I (n)^(a)^	Round sptids (n)^(a)^	Elongated sptids (n)^(a)^	Sertoli cells	Johnsen score
T1	OA	CS	0,482	0	20,7	33,7	38,5	33,5	13,5	9,6
T2	OA	CS	0,330	0	21,4	28,1	31,3	24,8	9,1	9,7
T3	OA	CS	0,444	0	16,1	37,8	32,5	28,1	10,3	9,5
T4	OA	CS	0,432	0	nd	nd	nd	nd	nd	nd
T5	OA	CS	0,306	0,001	nd	nd	nd	nd	nd	nd
T6	OA	CS	0,880	0	nd	nd	nd	nd	nd	nd
T7	OA	CS	0,532	0	nd	nd	nd	nd	nd	nd
T8	OA	CS	0,418	0	nd	nd	nd	nd	nd	nd
T9	OA	CS	0,400	0	25,7	43,6	33,2	26,5	15,3	9,9
T10	OA	CS	0,226	0	26,7	28,2	20,3	21,1	13,9	9,3
T11	OA	CS	0,292	0	22,8	28,5	16,6	16,0	14,2	9,0
T12	OA	CS	0,386	0	nd	nd	nd	nd	nd	nd
Mean			0,427		22,2	33,3	28,7	25,0	12,7	
T13	SA	SpF (rsMF)	0,003	0	23,4	36,0	19,7	10,1	12,2	8,4
T14	SSO	SpF (rsMF)	0,055	1,6	19,7	32,1	35,4	1,1	10,8	6,4
Mean			0,029		21,6	34,1	27,6	5,6	11,5	
T15	SA	SpF (scMF)	0,002	0	23,7	23,1	3,4	3,6	13,7	7,2
T16	SA	SpF (scMF)	0,006	0	19,5	17,6	4,0	2,0	14,1	6,7
T17	SSO	SpF (scMF)	0,022	0,01	15,8	26,0	6,1	2,1	11,8	6,4
T18	SSO	SpF (scMF)	0,002	0,02	17,2	38,8	5,6	1,4	15,1	6,3
T19	SA	SpF (scMF)	0	0	14,6	18,9	1,0	0,9	8,4	5,7
T20	SSO	SpF (scMF)	0,002	0,4	26,0	19,3	4,0	0,1	9,6	5,2
T21	SA	SpF (scMF)	0,001	0	22,5	39,6	0,0	0,0	16,0	5,0
T22	SA	SpF (scMF)	0	0	23,0	30,0	0,0	0,0	11,0	5,0
T23	SA	SpF (scMF)	0	0	17,6	18,2	0,0	0,0	20,0	5,0
T24	SA	SpF (scMF)	0	0	14,3	14,6	0,3	0,0	23,3	5,0
T25	SSO	SpF (scMF)	0,154	0,015	23,0	6,0	5,0	5,0	11,0	4,4
Mean			0,017		19,7	22,9	2,7	1,4	14,0	
T26	SA	SpF (sgMF)	0,048	0,009	12,7	23,1	18,7	10,6	15,3	8,1
T27	SSO	SpF (sgMF)	0,019	0,4	10,8	12,8	7,4	10,2	7,4	6,9
T28	SSO	SpF (sgMF)	0,02	0,45	11,6	17,4	4,7	3,7	13,9	6,2
T29	SA	SpF (sgMF)	0,07	0	11,7	18,1	0,1	0,0	11,7	4,9
T30	SA	SpF (sgMF)	0	0	9,0	10,6	0	0,0	10,9	4,8
T31	SA	SpF (sgMF)	0	0	10,9	12,9	0,2	0,0	9,0	4,7
T32	SA	SpF (sgMF)	0,002	0	15,0	7,0	1,2	0,0	12,0	4,7
T33	SA	SpF (sgMF)	0	0	4,0	1,4	0,1	0,0	6,6	3,6
T34	SA	SpF (sgMF)	0,001	0,004	8,3	12,6	0,0	0,0	6,6	3,5
T35	SA	SpF (sgMF)	0	0	8,5	0,2	0,0	0,0	5,2	2,8
Mean			0,016		10,2	11,6	3,2	2,4	9,9	
T36	SA	SCO	0	0	0,0	0,0	0,0	0,0	19,5	2,0
T37	SA	SCO	0	0	0,0	0,0	0,0	0,0	15,6	2,0
T38	SA	SCO	0	0	0,0	0,0	0,0	0,0	26,3	2,0
T39	SA	SCO	0	0	0,0	0,0	0,0	0,0	17,8	2,0
T40	SA	SCO	0,033	0	0,0	0,0	0,0	0,0	46,3	2,0
Mean					0,0	0,0	0,0	0,0	25,1	

Abbreviations: conc., concentration; CS, conserved spermatogenesis; nd, not determined; OA, obstructive azoospermia; TESE value, number of spermatozoa obtained directly from testicular biopsy; SA, secretory/non-obstructive azoospermia; SCO, Sertoli cell-only syndrome; Spcyte, spermatocyte; SpF, spermatogenic failure; Spgonia, spermatogonia; Sptid, spermatid; SSO, severe secretory/non-obstructive oligozoospermia; sgMF, maturation failure at spermatogonia level; scMF, maturation failure at spermatocyte level; rsMF, maturation failure at round spermatid level.

^a^The mean number of the different type of cells per tubule is given in each group.

**Table 2:** Pearson correlation coefficients and adjusted p values (r;p) between the significantly correlated molecular and histological parameters for the nine samples analysed in the testicular miRNA profiling.

	TESE-value	Elongated spermatids/tubule	AUC	95% CI	p-value
hsa-let-7b*	−0,703;p = 0,035	−0,719;p = 0,029	0,950	0,806–1,094	**0,027**
hsa-miR-106b*	0,718;p = 0,029	0,692;p = 0,039	0,250	−0,090–0,590	0,221
hsa-miR-10b*	−0,884;p = 0,002	−0,879;p = 0,002	1,000	1,000–1,000	**0,014**
***hsa-miR-122***	0,948;p = 0,0001	0,975;p < 0,0001	0,000	0,000–0,000	**0,014**
hsa-miR-124	0,904;p = 0,002	0,880;p = 0,004	0,125	−0,142–0,392	0,083
hsa-miR-148*	−0,845;p = 0,008	−0,842;p = 0,009	0,750	0,326–1,174	0,248
hsa-miR-15b	0,805;p = 0,009	0,833;p = 0,005	0,100	−0,121–0,321	0,05
hsa-miR-16-2*	0,879;p = 0,002	0,885;p = 0,002	0,100	−0,121–0,321	0,05
hsa-miR-182	0,951;p < 0,0001	0,970;p < 0,0001	0,100	−0,121–0,321	0,05
hsa-miR-194	0,828;p = 0,006	0,839;p = 0,005	0,150	−0,112–0,412	0,086
hsa-miR-202*	−0,814;p = 0,008	−0,800;p = 0,010	1,000	1,000–1,000	**0,014**
***hsa-miR-210***	−0,793;p = 0,011	−0,788;p = 0,012	0,950	0,806–1,094	**0,027**
hsa-miR-215	0,744;p = 0,022	0,738;p = 0,023	0,100	−0,119–0,319	0,05
hsa-miR-23a*	0,854;p = 0,007	0,815;p = 0,014	0,000	0,000–0,000	**0,025**
hsa-miR-26a	−0,696;p = 0,037	−0,692;p = 0,039	0,900	0,679–1,121	0,05
hsa-miR-26a-2*	−0,721;p = 0,028	−0,715;p = 0,030	1,000	1,000–1,000	**0,014**
hsa-miR-27b*	−0,786;p = 0,036	−0,759;p = 0,048	1,000	1,000–1,000	**0,034**
***hsa-miR-296-3p***	0,834;p = 0,005	0,868;p = 0,002	0,050	−0,094–0,194	**0,027**
hsa-miR-296-5p	0,733;p = 0,025	0,733;p = 0,025	0,150	−0,142–0,442	0,086
hsa-miR-30e*	0,761;p = 0,017	0,804;p = 0,009	0,200	−0,119–0,510	0,142
hsa-miR-31*	0,755;p = 0,030	0,711;p = 0,048	0,125	−0,142–0,392	0,083
hsa-miR-330-5p	−0,761;p = 0,028	−0,756;p = 0,031	0,750	0,326–1,174	0,248
hsa-miR-340*	0,749;p = 0,032	0,740;p = 0,036	0,000	0,000–0,000	**0,025**
hsa-miR-342-3p	−0,703;p = 0,035	−0,667;p = 0,050	0,700	0,319–1,081	0,327
***hsa-miR-34b***	0,921;p = 0,0004	0,931;p = 0,0002	0,050	−0,094–0,194	**0,027**
hsa-miR-34b*	0,882;p = 0,002	0,899;p = 0,001	0,050	−0,094–0,194	**0,027**
***hsa-miR-34c*****–*****5p***	0,862;p = 0,003	0,883;p = 0,002	0,050	−0,094-0,194	**0,027**
hsa-miR-375	0,787;p = 0,012	0,815;p = 0,007	0,100	−0,119–0,319	0,05
***hsa-miR-449a***	0,890;p = 0,001	0,915;p = 0,001	0,050	−0,094–0,194	**0,027**
***hsa-miR-449b***	0,836;p = 0,005	0,845;p = 0,004	0,050	−0,094–0,194	**0,027**
hsa-miR-449b*	0,807;p = 0,028	0,779;p = 0,039	0,000	0,000–0,000	**0,034**
hsa-miR-488	0,776;p = 0,014	0,784;p = 0,012	0,100	−0,121–0,321	0,05
hsa-miR-499-5p	0,766;p = 0,027	0,728;p = 0,041	0,188	−0,160–0,535	0,149
hsa-miR-502-3p	−0,703;p = 0,035	−0,726;p = 0,027	0,900	0,679–1,121	0,05
hsa-miR-504	−0.955;p = 0,001	−0,950;p = 0,001	0,833	0,500–1,167	0,157
***hsa-miR-511***	0,984;p < 0,0001	0,969;p < 0,0001	0,000	0,000–0,000	**0,025**
***hsa-miR-512-5p***	0,719;p = 0,029	0,713;p = 0,030	0,000	0,000–0,000	**0,014**
***hsa-miR-516a-5p***	0,695;p = 0,038	0,678;p = 0,045	0,000	0,000–0,000	**0,014**
***hsa-miR-516b***	0,999;p < 0,0001	0,999;p < 0,0001	0,000	0,000–0,000	**0,025**
hsa-miR-517a	0,672;p = 0,047	0,691;p = 0,039	0,150	−0,112–0,412	0,086
***hsa-miR-517b***	0,757;p = 0,018	0,744;p = 0,021	0,050	−0,094–0,194	**0,027**
***hsa-miR-517c***	0,854;p = 0,003	0,860;p = 0,003	0,000	0,000–0,000	**0,014**
hsa-miR-518a-3p	0,754;p = 0,019	0,781;p = 0,013	0,150	0,137–0,437	0,086
***hsa-miR-518e***	0,805;p = 0,009	0,781;p = 0,013	0,050	−0,094–0,194	**0,027**
hsa-miR-518e*	0,719;p = 0,045	0,714;p = 0,047	0,067	−0,118–0,251	0,053
***hsa-miR-518f***	0,759;p = 0,018	0,761;p = 0,017	0,050	−0,094–0,194	**0,027**
***hsa-miR-519a***	0,784;p = 0,012	0,773;p = 0,015	0,050	−0,094–0,194	**0,027**
hsa-miR-519c-3p	0,857;p = 0,003	0,875;p = 0,002	0,100	−0,119–0,319	0,05
hsa-miR-520a-3p	0,736;p = 0,024	0,779;p = 0,013	0,100	−0,119–0,319	0,05
hsa-miR-520a-5p	0,767;p = 0,016	0,771;p = 0,015	0,100	−0,119–0,319	0,05
hsa-miR-520d-5p	0,832;p = 0,040	0,828;p = 0,042	0,000	0,000–0,000	0,064
***hsa-miR-521***	0,933;p = 0,0002	0,950;p < 0,0001	0,000	0,000–0,000	**0,014**
hsa-miR-525-5p	0,776;p = 0,024	0,808;p = 0,015	0,067	−0,118–0,251	0,053
hsa-miR-532-3p	−0,704;p = 0,034	−0,717;p = 0,030	0,900	0,679–1,121	0,05
hsa-miR-548o	−0,770;p = 0,015	−0,767;p = 0,016	0,850	0,558–1,142	0,086
hsa-miR-551a	−0,825;p = 0,012	−0,822;p = 0,012	0,875	0,608–1,142	0,083
hsa-miR-551b	0,828;p = 0,011	0,854;p = 0,007	0,188	−0,126–0,501	0,149
hsa-miR-566	0,879;p = 0,009	0,880;p = 0,009	0,083	−0,143–0,309	0,077
hsa-miR-625*	0,973;p < 0,0001	0,954;p < 0,0001	0,100	−0,121–0,321	0,05
hsa-miR-629	0,847;p = 0,004	0,834;p = 0,005	0,250	−0,174–0,674	0,221
hsa-miR-643	0,914;p = 0,002	0,929;p = 0,001	0,100	−0,119–0,319	0,05
hsa-miR-769-5p	0,753;p = 0,019	0,728;p = 0,026	0,100	−0,119–0,319	0,05
hsa-miR-885-5p	0,884;p = 0,002	0,901;p = 0,001	0,100	−0,121–0,321	0,05
***hsa-miR-888***	0,991;p < 0,0001	0,984;p < 0,0001	0,000	0,000–0,000	**0,025**
hsa-miR-9	0,741;p = 0,022	0,802;p = 0,009	0,100	−0,119–0,319	0,05
***hsa-miR-96***	0,926;p = 0,0003	0,956;p < 0,0001	0,050	−0,094–0,194	**0,027**
hsa-miR-99b*	−0,737;p = 0,024	−0,744;p = 0,021	0,850	0,558–1,142	0,086
hsa-miR-505*	−0,882;p = 0,020	−0,877;p = 0,022	1,000	1,000–1,000	0,064

ROC analysis showing the predictive efficiency of miRNA expression values for determining the presence of intratesticular mature sperm cells (TESE value > 0,002; elongated spermatids/tubule > 0,1).

Only those miRNAs that presented significant correlation coefficients for both TESE value and elongated spermatids/tubules are indicated. Those miRNAs that resulted in good predictive accuracy (AUC > 0,900 for those negatively correlated and AUC < 0,05 for those positively correlated; p ≤ 0,03) after the ROC analysis are depicted in bold and italics.

TESE value is defined as the number of spermatozoa that were obtained directly after processing 100 mg of biopsy in 1 mL of medium.

**Table 3:** Clinical data of individuals included in the study of spermatozoa.

Patient No.	Group	Male age^(a)^	Spermiogram	Semen volume (mL)	Sperm count (×10^6^/mL)	Progressive motility (%)	Normal morphology (%)	Female age^(a)^	Female partner gynecological assessment
S1	Fertile	44	NORMO	1	120	68	9	38	Normal (default)
S2	Fertile	41	NORMO	4,5	174	46	11	36	Normal (default)
S3	Fertile	49	NORMO	4,5	122	46	11	39	Normal (default)
S4	Fertile	33	NORMO	4,5	75	46	8	31	Normal (default)
S5	Fertile	39	NORMO	1,5	96	58	10	34	Normal (default)
S6	Fertile	36	NORMO	3,5	42	42	6	33	Normal (default)
S7	Fertile	34	NORMO	4,5	40	65	3	35	Normal (default)
S8	Infertile	40	OLIGO (OT)	6,1	10	37	2	39	Normal
S9	Infertile	45	OLIGO (OT)	2,5	10	59	2	34	Normal
S10	Infertile	38	OLIGO (OT)	1,5	13	55	2	36	Ovulatory dysfunction
S11	Infertile	42	OLIGO (OT)	4,25	5	48	1	30	Normal
S12	Infertile	38	OLIGO (OT)	7,3	4	45	0	38	Ovulatory dysfunction
S13	Infertile	31	OLIGO (OAT)	4	9	29	1	31	Normal
S14	Infertile	38	OLIGO	1,9	11	34	4	38	Normal
S15	Infertile	36	OLIGO	3,75	12	46	3	36	Normal
S16	Infertile	32	OLIGO (OAT)	4	7	28	1	34	Ovulatory dysfunction

Abbreviations: NORMO, normozoospermia; OLIGO, oligozoospermia; OT, oligo and teratozoospermia; OAT, oligo, asteno and teratozoospermia. ^a^age at the time of clinical assessment.
