# Supplementary material for: Altered miRNA Signature of Developing Germ-cells in Infertile Patients Relates to the Severity of Spermatogenic Failure and Persists in Spermatozoa
Source: Sci Rep. 2015 Dec 9;5:17991. doi: 10.1038/srep17991 (PMC4673613; doi:10.1038/srep17991)
Supplement: Supplementary Information [file srep17991-s1.pdf]

ALTERED miRNA SIGNATURE OF DEVELOPING GERM-CELLS IN INFERTILE PATIENTS RELATES TO THE SEVERITY OF SPERMATOGENIC FAILURE AND PERSISTS IN SPERMATOZOA

Xavier Muñoz[1#], Ana Mata[2], Lluís Bassas[2], Sara Larriba[1]*

ONLINE-ONLY MATERIAL

In this section we have included supporting information concerning four tables.

Supplemental Table S1. Summary of miRNA expression data in scMF and in SCO phenotypes related to CS

Statistically increased miRNA expression levels are depicted in green (dark colour intensity is related to a fold-change increase >2);
statistically decreased miRNA expression levels are depicted in red (dark colour intensity is related to a fold-change decrease <2),
when compared with controls. Those miRNAs clustered in chr. 19 are depicted in italic.
Cp values >38 are depicted in bold.
* p<0,05; ** p<0,005. Statistically significant p-values are depicted in bold.

miRNA	Average *Cp* CS	Average *Cp* *scMF*	Average *Cp* SCO	miRNA expression CS	scMF	SCO	***p*-value** scMF-CS	SCO-CS	Sequence
hsa-let-7a	28,10	28,35	26,55	1	0,923	2,434*	> 0,10	**0,0060**	ugagguaguagguuguauaguu
hsa-let-7a*	33,51	33,30	32,37	1	1,268	1,829	> 0,10	0,0592	
hsa-let-7a-2*	35,89	35,52	35,42	1	1,416	1,150	> 0,10	> 0,10	
hsa-let-7b	25,85	25,92	24,32	1	1,048	2,398**	> 0,10	**0,0024**	ugagguaguagguugugugguu
hsa-let-7b*	31,54	31,35	30,63	1	1,251	1,566*	> 0,10	**0,0086**	cuauacaaccuacugccuuccc
hsa-let-7c	28,97	29,16	27,33	1	0,964	2,583*	> 0,10	**0,0106**	ugagguaguagguuguaugguu
hsa-let-7d	31,01	31,35	29,63	1	0,870	2,167*	> 0,10	**0,0411**	agagguaguagguugcauaguu
hsa-let-7d*	25,40	25,33	25,30	1	0,974	0,755	> 0,10	> 0,10	
hsa-let-7e	28,95	28,87	27,71	1	1,162	1,963*	> 0,10	**0,0097**	ugagguaggagguuguauaguu
hsa-let-7f	29,29	29,23	27,99	1	1,147	2,044	> 0,10	0,0508	
hsa-let-7f-1*	34,05	34,09	32,89	1	1,073	1,869	> 0,10	**0,0270**	cuauacaaucuauugccuuccc
hsa-let-7f-2*	32,56	33,04	31,64	1	0,789	1,577	> 0,10	0,0915	
hsa-let-7g	27,85	27,82	26,45	1	1,128	2,200*	> 0,10	**0,0024**	ugagguaguaguuuguacaguu
hsa-let-7g*	33,55	33,63	31,99	1	1,043	2,456*	> 0,10	**0,0183**	cuguacaggccacugccuugc
hsa-let-7i	29,41	29,51	27,97	1	1,027	2,274*	> 0,10	**0,0220**	ugagguaguaguuugugcuguu
hsa-let-7i*	31,54	31,33	30,31	1	1,272	1,950*	> 0,10	**0,0226**	cugcgcaagcuacugccuugcu
hsa-miR-1	32,62	31,62	31,80	1	2,199	1,470	> 0,10	0,0842	
hsa-miR-100	31,92	31,75	30,57	1	1,238	2,131**	0,1011	**0,0157**	aacccguagauccgaacuugug
hsa-miR-101	28,11	28,09	27,16	1	1,119	1,617	> 0,10	> 0,10	
hsa-miR-101*	35,71	35,78	35,05	1	1,055	1,319	> 0,10	> 0,10	
hsa-miR-103	27,32	27,31	26,27	1	1,105	1,720*	> 0,10	**0,0116**	agcagcauuguacagggcuauga
hsa-miR-103 (duplicate)	27,06	27,06	25,99	1	1,102	1,760*	> 0,10	**0,0298**	
hsa-miR-103-2*	35,02	35,26	33,76	1	0,919	1,972*	> 0,10	**0,0059**	agcuucuuuacagugcugccuug
hsa-miR-103-as	36,60	36,99	34,59	1	1,067	3,371	> 0,10	> 0,10	

hsa-miR-105	35,69	36,19	**40,00**	1	0,781	0,042**	> 0,10	**0,0022**	ucaaaugcucagacuccuguggu
hsa-miR-106a	28,34	28,51	27,68	1	0,980	1,324	> 0,10	> 0,10	
hsa-miR-106b	33,94	34,26	33,67	1	0,885	1,007	> 0,10	> 0,10	
hsa-miR-106b*	33,08	33,90	35,28	1	0,626	0,181*	> 0,10	**0,0123**	ccgcacuguggguacuugcugc
hsa-miR-107	28,09	28,08	27,01	1	1,112	1,757*	> 0,10	**0,0281**	agcagcauuguacagggcuauca
hsa-miR-10a	34,92	34,94	36,12	1	1,085	0,363	> 0,10	0,0701	
hsa-miR-10b	30,67	30,35	29,59	1	1,370	1,759	> 0,10	0,0747	
hsa-miR-10b*	**39,63**	37,66	36,65	1	4,340*	6,610**	**0,0497**	**0,0002**	acagauucgauucuaggggaau
hsa-miR-1185	35,10	35,34	33,95	1	0,930	1,841	> 0,10	0,0692	
hsa-miR-1201	31,79	32,11	31,82	1	0,883	0,816	> 0,10	> 0,10	
hsa-miR-122	32,54	36,57	**39,48**	1	0,067*	0,007*	**0,0168**	**0,0233**	uggagugugacaaugguguuug
hsa-miR-124	32,08	33,42	34,92	1	0,433*	0,120*	**0,0454**	**0,0244**	uaaggcacgcggugaaugcc
hsa-miR-1247	31,83	32,00	30,30	1	0,979	2,400	> 0,10	0,0824	
hsa-miR-1248	32,14	31,78	32,02	1	1,416	0,907	> 0,10	> 0,10	
hsa-miR-125a-3p	34,19	34,21	32,85	1	1,091	2,116*	> 0,10	**0,0319**	acaggugagguucuugggagcc
hsa-miR-125a-5p	26,44	26,33	25,24	1	1,185	1,920*	> 0,10	**0,0012**	ucccugagacccuuuaaccuguga
hsa-miR-125b	24,60	24,41	23,14	1	1,257	2,305*	0,0912	**0,0009**	ucccugagacccuaacuuguga
hsa-miR-125b-1*	34,18	33,96	32,55	1	1,279	2,574*	> 0,10	**0,0076**	acggguuaggcucuugggagcu
hsa-miR-125b-2*	34,17	34,13	32,87	1	1,128	2,050*	> 0,10	**0,0195**	ucacaagucaggcucuugggac
hsa-miR-126	27,32	27,36	26,97	1	1,068	1,057	> 0,10	> 0,10	
hsa-miR-126*	30,84	30,97	30,51	1	1,012	1,048	> 0,10	> 0,10	
hsa-miR-1260	27,89	28,27	28,34	1	0,844	0,611	> 0,10	> 0,10	
hsa-miR-1270	**40,00**	**39,72**	36,85	1	1,330	7,405	> 0,10	0,0892	cuggagauauggaagagcugugu
hsa-miR-1271	**40,00**	37,74	35,43	1	5,288	17,602	> 0,10	> 0,10	cuuggcaccuagcaagcacuca
hsa-miR-127-3p	30,03	29,90	28,26	1	1,207	2,850	> 0,10	0,0660	
hsa-miR-127-5p	33,82	34,57	32,80	1	0,654	1,691	> 0,10	> 0,10	
hsa-miR-129*	**40,00**	**40,00**	35,22	1	1,102	22,870	> 0,10	> 0,10	aagcccuuaccccaaaaaguau
hsa-miR-129-3p	32,24	31,76	30,56	1	1,539	2,681	> 0,10	> 0,10	
hsa-miR-129-5p	32,46	32,71	30,46	1	0,928	3,342	> 0,10	> 0,10	
hsa-miR-1296	31,85	31,90	32,29	1	1,063	0,613*	> 0,10	**0,0207**	uuagggcccuggcuccaucucc
hsa-miR-130a	31,33	31,18	30,24	1	1,221	1,768	> 0,10	0,0962	
hsa-miR-130b	36,72	36,49	36,27	1	1,292	1,144	> 0,10	> 0,10	
hsa-miR-130b*	35,91	36,16	36,37	1	0,865	0,607	> 0,10	> 0,10	

hsa-miR-132	30,85	31,09	30,54	1	0,931	1,035	> 0,10	> 0,10	
hsa-miR-132*	33,12	34,36	33,50	1	0,466	0,644	0,0779	> 0,10	
hsa-miR-133a	33,96	32,93	32,95	1	2,245	1,674	> 0,10	0,0895	
hsa-miR-133b	32,68	31,25	31,74	1	2,978	1,603	> 0,10	> 0,10	
hsa-miR-134	33,16	32,54	31,48	1	1,694	2,663	> 0,10	0,0786	
hsa-miR-135a	30,07	29,99	28,50	1	1,164	2,470	> 0,10	0,0851	
hsa-miR-135a*	35,64	36,22	35,58	1	0,764	0,902	> 0,10	> 0,10	
hsa-miR-135b	30,77	30,93	28,74	1	0,990	3,411*	> 0,10	**0,0255**	uauggcuuuucauuccuauguga
hsa-miR-136	28,90	29,02	27,40	1	1,010	2,359	> 0,10	> 0,10	
hsa-miR-138	34,74	34,97	**40,00**	1	0,942	0,022*	> 0,10	**0,0478**	agcugguguugugaaucaggccg
hsa-miR-139-5p	31,88	32,37	31,32	1	0,785	1,229	> 0,10	> 0,10	
hsa-miR-140-3p	30,24	30,08	28,96	1	1,224	2,023**	> 0,10	**0,0017**	uaccacaggguagaaccacgg
hsa-miR-140-5p	31,70	31,75	30,69	1	1,104	1,748*	> 0,10	**0,0435**	cagugguuuuacccuaugguag
hsa-miR-141	35,73	36,67	35,77	1	0,615	0,834	> 0,10	> 0,10	
hsa-miR-142-3p	29,81	29,55	28,72	1	1,319	1,777	> 0,10	> 0,10	
hsa-miR-142-5p	34,87	34,40	33,70	1	1,529	1,876	> 0,10	> 0,10	
hsa-miR-143	25,46	25,00	24,25	1	1,521	1,932*	> 0,10	**0,0362**	ugagaugaagcacuguagcuc
hsa-miR-143*	31,28	30,89	30,43	1	1,450	1,509	> 0,10	0,0803	
hsa-miR-144	28,15	29,94	30,01	1	0,319	0,229*	> 0,10	**0,0210**	uacaguauagaugauguacu
hsa-miR-144*	33,67	34,88	35,23	1	0,478	0,284	> 0,10	> 0,10	
hsa-miR-145	24,82	24,42	23,69	1	1,456	1,826	> 0,10	0,0842	
hsa-miR-145*	32,17	32,07	31,15	1	1,179	1,696	> 0,10	0,0667	
hsa-miR-1468	36,03	36,34	34,75	1	0,895	2,025	> 0,10	> 0,10	
hsa-miR-146a	35,79	35,24	33,41	1	1,618	4,335	> 0,10	0,0766	
hsa-miR-146b-3p	35,37	36,30	35,42	1	0,578	0,807	> 0,10	> 0,10	
hsa-miR-146b-5p	34,29	34,38	33,19	1	1,036	1,784*	> 0,10	**0,0355**	ugagaacugaauuccauaggcu
hsa-miR-147b	34,24	34,92	35,80	1	0,681	0,256	> 0,10	**0,0009**	gugugcggaaaugcuucugcua
hsa-miR-148a	30,04	29,83	28,53	1	1,277	2,373*	> 0,10	**0,0158**	ucagugcacuacagaacuuugu
hsa-miR-148a*	**40,00**	36,28	36,45	1	15,548	9,769*	> 0,10	**0,0396**	aaaguucugagacacuccgacu
hsa-miR-148b	31,05	31,15	30,03	1	1,024	1,688	> 0,10	0,0538	
hsa-miR-149	31,41	31,02	29,99	1	1,442	2,230*	> 0,10	**0,0312**	ucuggcuccgugucuucacuccc
hsa-miR-149*	33,95	34,25	34,22	1	0,901	0,692*	> 0,10	**0,0330**	agggagggacgggggcugugc
hsa-miR-150	31,08	30,76	30,57	1	1,373	1,188	> 0,10	> 0,10	

hsa-miR-151-3p	31,68	31,74	30,23	1	1,060	2,284*	> 0,10	**0,0117**	cuagacugaagcuccuugagg
hsa-miR-151-5p	29,17	29,20	27,84	1	1,079	2,101*	> 0,10	**0,0427**	ucgaggagcucacagucuagu
hsa-miR-152	29,98	30,36	29,48	1	0,850	1,185	> 0,10	> 0,10	
hsa-miR-153	31,05	31,95	31,84	1	0,587	0,481	> 0,10	> 0,10	
hsa-miR-1537	36,68	**40,00**	36,38	1	0,110	1,027	> 0,10	> 0,10	aaaaccgucuaguuacaguugu
hsa-miR-154	31,78	31,57	29,86	1	1,278	3,155	> 0,10	0,0528	
hsa-miR-154*	35,96	36,10	35,04	1	0,999	1,586	> 0,10	> 0,10	
hsa-miR-155	34,17	34,59	33,69	1	0,821	1,159	> 0,10	> 0,10	
hsa-miR-15a	27,49	27,72	26,91	1	0,936	1,248	> 0,10	> 0,10	
hsa-miR-15a*	35,30	36,11	34,96	1	0,579	1,074	> 0,10	> 0,10	
hsa-miR-15b	28,01	28,70	28,37	1	0,682	0,649*	0,0986	**0,0235**	uagcagcacaucaugguuuaca
hsa-miR-15b*	34,50	34,37	34,13	1	1,186	1,057	> 0,10	> 0,10	
hsa-miR-16	26,50	26,80	26,73	1	0,893	0,714	> 0,10	> 0,10	
hsa-miR-16-2*	32,50	33,34	33,75	1	0,614*	0,350*	**0,0285**	**0,0054**	ccaauauuacugugcugcuuua
hsa-miR-17	31,87	32,41	31,14	1	0,757	1,387	> 0,10	> 0,10	
hsa-miR-17*	30,98	30,98	29,61	1	1,108	2,159*	> 0,10	**0,0313**	acugcagugaaggcacuuguag
hsa-miR-181a	28,81	28,73	27,49	1	1,160	2,082**	> 0,10	**0,0123**	aacauucaacgcugucggugagu
hsa-miR-181a*	35,74	35,20	34,01	1	1,600	2,766	> 0,10	0,0719	
hsa-miR-181a-2*	35,88	36,00	34,41	1	0,940	2,303	> 0,10	> 0,10	
hsa-miR-181b	32,99	32,87	30,98	1	1,204	3,366*	> 0,10	**0,0029**	aacauucauugcugucggugggu
hsa-miR-181c	35,01	34,10	34,26	1	2,066	1,399	0,0879	> 0,10	
hsa-miR-181c*	35,99	35,52	36,72	1	1,525	0,505	> 0,10	> 0,10	
hsa-miR-181d	35,81	35,63	34,52	1	1,244	2,030	> 0,10	> 0,10	
hsa-miR-182	34,40	37,20	**40,00**	1	0,157*	0,017*	**0,0292**	**0,0304**	uuuggcaaugguagaacucacacu
hsa-miR-182*	33,74	35,35	36,55	1	0,356	0,103	> 0,10	> 0,10	
hsa-miR-185	32,20	32,67	32,20	1	0,798	0,838	> 0,10	> 0,10	
hsa-miR-186	30,61	30,53	29,04	1	1,165	2,471**	> 0,10	**0,0072**	caaagaauucuccuuuugggcu
hsa-miR-187	30,72	30,71	28,91	1	1,106	2,997	> 0,10	0,0831	
hsa-miR-187*	35,80	36,04	35,40	1	1,036	1,139	> 0,10	> 0,10	
hsa-miR-188-5p	34,92	34,80	34,18	1	1,194	1,392*	> 0,10	**0,0372**	caucccuugcaugguggaggg
hsa-miR-18a	33,43	33,77	34,22	1	0,858	0,477	> 0,10	> 0,10	
hsa-miR-18a*	33,44	33,57	33,36	1	1,003	0,877	> 0,10	> 0,10	
hsa-miR-18b	32,21	32,66	32,61	1	0,805	0,632	> 0,10	> 0,10	

hsa-miR-190	32,83	33,27	32,08	1	0,810	1,402**	> 0,10	**0,0079**	ugauauguuugauauauuaggu
hsa-miR-1908	36,22	36,77	34,46	1	0,807	2,820	> 0,10	> 0,10	
hsa-miR-191	30,13	30,50	29,55	1	0,852	1,245	> 0,10	0,1030	
hsa-miR-191 (duplicate)	30,26	30,49	29,60	1	0,943	1,318	> 0,10	0,1030	
hsa-miR-191*	33,98	33,42	35,55	1	1,631	0,281	> 0,10	0,0867	
hsa-miR-1911*	**40,00**	**40,00**	**38,05**	1	1,102	3,224	> 0,10	> 0,10	caccaggcauuguggucucc
hsa-miR-192	32,56	32,56	33,51	1	1,100	0,429	> 0,10	> 0,10	
hsa-miR-193a-3p	34,57	34,49	33,35	1	1,164	1,936	> 0,10	> 0,10	
hsa-miR-193a-5p	35,95	36,00	35,19	1	1,069	1,418	> 0,10	0,0767	
hsa-miR-193b	29,12	29,07	27,52	1	1,143	2,536*	> 0,10	**0,0071**	aacuggcccucaaagucccgcu
hsa-miR-193b*	33,91	34,02	32,36	1	1,058	2,536	> 0,10	> 0,10	
hsa-miR-194	34,27	35,12	35,22	1	0,611	0,432	> 0,10	> 0,10	
hsa-miR-195	27,13	27,23	26,05	1	1,027	1,759	> 0,10	0,0714	
hsa-miR-195*	34,89	34,88	34,01	1	1,186	1,532	> 0,10	0,1011	
hsa-miR-196a	36,04	33,61	35,35	1	6,247	1,415	> 0,10	> 0,10	
hsa-miR-196b	37,72	35,33	36,87	1	5,361	1,376	> 0,10	> 0,10	
hsa-miR-197	30,48	30,44	29,30	1	1,130	1,888**	> 0,10	**0,0037**	uucaccaccuucuccacccagc
hsa-miR-1974	21,67	21,91	21,41	1	0,927	0,995	> 0,10	> 0,10	
hsa-miR-199a-3p	28,61	28,61	26,91	1	1,107	2,722	> 0,10	0,0824	
hsa-miR-199a-5p	27,18	26,91	25,70	1	1,326	2,325	> 0,10	0,0594	
hsa-miR-199b-5p	31,73	31,90	30,52	1	0,983	1,938	> 0,10	> 0,10	
hsa-miR-19a	31,43	31,57	30,72	1	1,002	1,360	> 0,10	> 0,10	
hsa-miR-19b	26,34	26,48	25,60	1	1,000	1,399	> 0,10	0,0550	
hsa-miR-19b-1*	34,53	35,01	34,42	1	0,782	0,887	> 0,10	> 0,10	
hsa-miR-200a	35,24	35,76	35,85	1	0,770	0,546	> 0,10	> 0,10	
hsa-miR-200b	36,28	36,63	36,37	1	0,841	0,809	> 0,10	> 0,10	
hsa-miR-202	27,25	27,02	25,55	1	1,344	2,819*	> 0,10	**0,0438**	agagguauagggcaugggaa
hsa-miR-202*	27,70	26,79	25,94	1	2,069*	2,838*	**0,0090**	**0,0063**	uuccuaugcauauacuucuuug
hsa-miR-204	27,49	27,32	26,04	1	1,241	2,285*	> 0,10	**0,0308**	uucccuuugucauccuaugccu
hsa-miR-205	35,52	35,45	**40,00**	1	1,286	0,039*	> 0,10	> 0,10	uccuucauuccaccggagucug
hsa-miR-20a	27,33	27,52	26,96	1	0,966	1,071	> 0,10	> 0,10	
hsa-miR-20a*	33,15	32,92	32,00	1	1,296	1,849*	> 0,10	**0,0313**	acugcauuaugagcacuuaaag
hsa-miR-20b	36,03	35,76	**40,00**	1	1,426	0,053	> 0,10	0,0501	caaagugcucauagugcagguag

hsa-miR-21	27,32	27,18	26,11	1	1,220	1,930*	> 0,10	**0,0122**	uagcuuaucagacugauguuga
hsa-miR-21*	35,07	34,80	33,19	1	1,325	3,072*	> 0,10	**0,0393**	caacaccagucgaugggcugu
hsa-miR-210	31,77	31,37	30,57	1	1,446*	1,903*	**0,0263**	**0,0270**	cugugcgugugacagcggcuga
hsa-miR-212	34,12	35,35	33,94	1	0,505	0,950	0,0535	> 0,10	
hsa-miR-214	30,61	30,47	28,99	1	1,213	2,555*	> 0,10	**0,0324**	acagcaggcacagacaggcagu
hsa-miR-214*	31,88	31,74	30,32	1	1,211	2,457	> 0,10	0,0993	
hsa-miR-215	33,84	34,24	34,71	1	0,834	0,458*	> 0,10	**0,0292**	augaccuaugaauugacagac
hsa-miR-216a	35,25	35,99	**40,00**	1	0,687	0,032	> 0,10	0,1055	uaaucucagcuggcaacuguga
hsa-miR-218	33,41	33,29	32,53	1	1,279	1,533	> 0,10	> 0,10	
hsa-miR-219-5p	33,14	34,29	32,17	1	0,497**	1,632	**0,0067**	0,0718	ugauuguccaaacgcaauucu
hsa-miR-22	29,87	29,81	28,26	1	1,143	2,549*	> 0,10	**0,0096**	aagcugccaguugaagaacugu
hsa-miR-22*	30,33	30,53	29,14	1	0,960	1,909*	> 0,10	**0,0349**	aguucuucaguggcaagcuuua
hsa-miR-221	32,57	32,18	31,34	1	1,446	1,960	> 0,10	0,0987	
hsa-miR-222	31,32	31,15	30,86	1	1,236	1,142	> 0,10	> 0,10	
hsa-miR-223	30,51	30,16	29,15	1	1,404	2,147	> 0,10	0,0988	
hsa-miR-23a	27,98	27,90	26,76	1	1,164	1,940*	> 0,10	**0,0283**	aucacauugccagggauuucc
hsa-miR-23a*	35,25	**40,00**	**40,00**	1	0,039	0,030	> 0,10	> 0,10	gggguuccuggggaugggauuu
hsa-miR-23b	27,47	27,37	26,47	1	1,185	1,673	> 0,10	0,0916	
hsa-miR-24	26,81	26,63	25,68	1	1,249	1,830*	> 0,10	**0,0270**	uggcucaguucagcaggaacag
hsa-miR-24-1*	34,11	34,06	33,42	1	1,138	1,231	> 0,10	> 0,10	
hsa-miR-24-2*	34,12	33,88	33,35	1	1,399	1,426	> 0,10	> 0,10	
hsa-miR-25	31,47	31,93	31,40	1	0,803	0,873	> 0,10	> 0,10	
hsa-miR-26a	26,33	26,00	25,07	1	1,386	1,993*	0,0649	**0,0033**	uucaaguaauccaggauaggcu
hsa-miR-26a-1*	34,39	35,01	34,57	1	0,668	0,736	> 0,10	> 0,10	
hsa-miR-26a-2*	**40,00**	**38,61**	37,77	1	2,885	3,917	0,0568	0,0795	ccuauucuugauuacuuguuuc
hsa-miR-26b	30,00	30,18	29,05	1	0,973	1,614	> 0,10	> 0,10	
hsa-miR-26b*	35,29	36,37	34,64	1	0,551	1,295	> 0,10	> 0,10	
hsa-miR-27a	27,53	27,45	26,41	1	1,172	1,823*	> 0,10	**0,0357**	uucacaguggcuaaguuccgc
hsa-miR-27b	27,55	27,47	26,52	1	1,162	1,691	> 0,10	0,0586	
hsa-miR-27b*	36,81	34,79	35,35	1	3,893	2,285*	> 0,10	**0,0498**	agagcuuagcugauuggugaac
hsa-miR-28-3p	32,23	32,35	31,22	1	1,017	1,682*	> 0,10	**0,0219**	cacuagauugugagcuccugga
hsa-miR-28-5p	31,43	31,53	30,47	1	1,026	1,620*	> 0,10	**0,0436**	aaggagcucacagucuauugag
hsa-miR-296-3p	33,14	34,78	34,89	1	0,353	0,248*	0,0821	**0,0387**	gaggguugggguggaggcucucc

hsa-miR-296-5p	31,70	32,27	32,49	1	0,743	0,483*	0,0778	**0,0029**	agggcccccccucaauccugu
hsa-miR-299-3p	36,53	36,34	34,53	1	1,248	3,295	> 0,10	> 0,10	
hsa-miR-299-5p	32,72	32,22	30,87	1	1,560	3,019*	> 0,10	**0,0436**	ugguuuaccgucccacauacau
hsa-miR-29a	28,14	28,22	26,89	1	1,043	1,985*	> 0,10	**0,0268**	uagcaccaucugaaaucgguua
hsa-miR-29a*	32,33	32,53	31,13	1	0,958	1,910*	> 0,10	**0,0198**	acugauuucuuuugguguucag
hsa-miR-29b	28,91	28,78	27,82	1	1,206	1,768*	> 0,10	**0,0445**	uagcaccauuugaaaucaguguu
hsa-miR-29b-1*	33,46	33,55	32,69	1	1,034	1,417	> 0,10	> 0,10	
hsa-miR-29b-2*	32,54	32,85	31,45	1	0,886	1,769	> 0,10	0,0986	
hsa-miR-29c	27,43	27,36	26,18	1	1,156	1,983*	> 0,10	**0,0244**	uagcaccauuugaaaucgguua
hsa-miR-29c*	32,89	32,88	31,43	1	1,111	2,296**	> 0,10	**0,0002**	ugaccgauuucuccugguguuc
hsa-miR-301a	32,52	33,03	32,29	1	0,774	0,978	0,0991	> 0,10	
hsa-miR-30a*	31,95	32,84	32,46	1	0,597	0,587*	> 0,10	**0,0344**	cuuucagucggauguuugcagc
hsa-miR-30b	28,23	28,21	27,37	1	1,111	1,515	> 0,10	0,1225	
hsa-miR-30c	27,51	27,58	26,86	1	1,048	1,306*	> 0,10	**0,0229**	uguaaacauccuacacucucagc
hsa-miR-30d	31,62	31,60	30,67	1	1,118	1,612	> 0,10	0,0532	
hsa-miR-30d*	33,47	34,12	34,12	1	0,700	0,531	> 0,10	> 0,10	
hsa-miR-30e	32,56	32,42	31,92	1	1,212	1,293	> 0,10	> 0,10	
hsa-miR-30e*	31,06	31,58	31,19	1	0,766	0,762	> 0,10	> 0,10	
hsa-miR-31	30,50	31,05	**40,00**	1	0,756	0,001*	> 0,10	**0,0100**	aggcaagaugcuggcauagcu
hsa-miR-31*	33,79	34,54	36,70	1	0,655	0,101	> 0,10	**0,0480**	ugcuaugccaacauauugccau
hsa-miR-32	29,81	29,90	28,96	1	1,031	1,495	> 0,10	0,1064	
hsa-miR-320a	28,04	27,99	26,72	1	1,146	2,085**	> 0,10	**0,0007**	aaaagcugggguugagagggcga
hsa-miR-320b	28,73	28,45	27,25	1	1,333	2,322**	0,0784	**0,0030**	aaaagcugggguugagagggcaa
hsa-miR-323-3p	36,72	35,91	34,26	1	1,934	4,581	> 0,10	> 0,10	
hsa-miR-324-3p	31,14	31,14	29,94	1	1,106	1,913*	> 0,10	**0,0034**	acugccccaggugcugcugg
hsa-miR-324-5p	31,81	31,73	30,69	1	1,167	1,816*	> 0,10	**0,0105**	cgcauccccuagggcauuggugu
hsa-miR-326	33,22	33,44	32,55	1	0,951	1,326	> 0,10	> 0,10	
hsa-miR-328	31,80	31,68	30,57	1	1,198	1,967**	> 0,10	**0,0015**	cuggcccucucugcccuuccgu
hsa-miR-329	35,79	34,64	33,34	1	2,442	4,552*	> 0,10	**0,0100**	aacacaccugguuaaccucuuu
hsa-miR-330-5p	**40,00**	37,40	36,27	1	6,684	10,122	> 0,10	> 0,10	ucucugggccugugucuuaggc
hsa-miR-331-3p	30,03	29,94	28,75	1	1,172	2,025	> 0,10	0,0590	
hsa-miR-335	32,11	32,14	31,52	1	1,083	1,260	> 0,10	> 0,10	
hsa-miR-337-3p	35,44	35,57	34,30	1	1,009	1,837	> 0,10	0,0504	

hsa-miR-337-5p	36,98	36,30	34,88	1	1,772	3,579	> 0,10	0,1079	
hsa-miR-338-3p	30,73	31,27	30,06	1	0,755	1,321	> 0,10	> 0,10	
hsa-miR-339-3p	33,14	32,86	31,51	1	1,343	2,578**	> 0,10	**0,0078**	ucccuguccuccaggagcucacg
hsa-miR-339-5p	31,88	31,51	30,38	1	1,424	2,356	0,0748	0,0517	
hsa-miR-33a	30,04	30,02	28,26	1	1,113	2,853	> 0,10	0,0785	
hsa-miR-33a*	**39,02**	35,93	34,50	1	9,356	19,140	> 0,10	> 0,10	caauguuuccacagugcaucac
hsa-miR-33b	32,54	32,70	30,98	1	0,923	2,467*	> 0,10	**0,0092**	gugcauugcuguugcauugc
hsa-miR-340	36,42	36,41	36,43	1	1,110	0,829	> 0,10	> 0,10	
hsa-miR-340*	35,47	36,42	36,58	1	0,824	0,381	> 0,10	> 0,10	
hsa-miR-342-3p	30,60	30,23	29,89	1	1,422	1,370	0,0916	0,0554	
hsa-miR-34a	27,58	27,72	26,34	1	0,998	1,962*	> 0,10	**0,0187**	uggcagugucuuagcugguugu
hsa-miR-34a*	31,97	31,90	30,77	1	1,159	1,920	> 0,10	0,0655	
hsa-miR-34b	30,46	32,64	**38,70**	1	0,243*	0,002*	**0,0178**	**0,0147**	caaucacuaacuccacugccau
hsa-miR-34b*	27,42	29,75	33,84	1	0,219*	0,010*	**0,0414**	**0,0124**	uaggcagugucauuagcugauug
hsa-miR-34c-5p	27,21	29,25	34,16	1	0,268	0,007*	0,0528	**0,0199**	aggcaguguaguuagcugauugc
hsa-miR-361-3p	32,23	32,16	30,99	1	1,164	1,977	> 0,10	0,0813	
hsa-miR-362-3p	36,25	36,57	35,33	1	0,883	1,578	> 0,10	> 0,10	
hsa-miR-362-5p	37,11	36,99	35,54	1	1,477	2,475	> 0,10	0,0904	
hsa-miR-363	35,62	35,99	36,14	1	0,852	0,516	> 0,10	> 0,10	
hsa-miR-365	29,88	29,94	28,51	1	1,054	2,156*	> 0,10	**0,0101**	uaaugccccuaaaaauccuuau
hsa-miR-369-5p	34,65	34,33	33,62	1	1,372	1,704	> 0,10	> 0,10	
hsa-miR-370	32,86	33,21	31,45	1	0,865	2,224	> 0,10	0,0976	
hsa-miR-371-3p	36,46	37,34	**40,00**	1	0,600	0,072	> 0,10	> 0,10	aagugccgccaucuuuugagugu
hsa-miR-372	33,38	35,11	36,61	1	0,332	0,079	> 0,10	> 0,10	
hsa-miR-373	35,25	35,98	**40,00**	1	0,712	0,031	> 0,10	0,0522	gaagugcuucgauuuuggggugu
hsa-miR-373*	34,59	36,24	35,78	1	0,376	0,333	> 0,10	> 0,10	
hsa-miR-374a	33,08	33,08	31,80	1	1,100	2,022**	> 0,10	**0,0076**	uuauaauacaaccugauaagug
hsa-miR-374b	31,69	31,32	30,56	1	1,426	1,833*	> 0,10	**0,0246**	auauaauacaaccugcuaagug
hsa-miR-374b*	**40,00**	**40,00**	37,17	1	1,102	5,936**	> 0,10	**0,0138**	cuuagcagguuguauuaucauu
hsa-miR-375	32,75	34,70	**40,00**	1	0,285	0,005**	> 0,10	**0,0214**	uuuguucguucggcucgcguga
hsa-miR-376a	31,12	31,24	29,82	1	1,013	2,060	> 0,10	0,0932	
hsa-miR-376b	33,75	33,83	31,74	1	1,040	3,360	> 0,10	0,0527	
hsa-miR-376c	31,34	31,40	29,61	1	1,053	2,764	> 0,10	> 0,10	

hsa-miR-377	31,84	31,83	30,19	1	1,109	2,615	> 0,10	0,0950	
hsa-miR-378	32,45	32,89	32,36	1	0,812	0,892	> 0,10	> 0,10	
hsa-miR-379	35,08	35,16	33,38	1	1,039	2,707	> 0,10	> 0,10	
hsa-miR-381	35,30	35,06	33,19	1	1,303	3,593*	> 0,10	**0,0437**	uauacaagggcaagcucucugu
hsa-miR-382	33,15	33,18	31,43	1	1,081	2,742	> 0,10	> 0,10	
hsa-miR-383	37,26	36,76	36,34	1	1,730	1,636	> 0,10	> 0,10	
hsa-miR-409-3p	32,37	32,07	30,74	1	1,356	2,577	> 0,10	0,0651	
hsa-miR-409-5p	35,45	35,58	35,07	1	1,005	1,087	> 0,10	> 0,10	
hsa-miR-410	35,76	35,27	34,29	1	1,551	2,317	> 0,10	> 0,10	
hsa-miR-411	31,78	31,95	30,16	1	0,978	2,570	> 0,10	> 0,10	
hsa-miR-421	34,90	35,00	33,50	1	1,027	2,205	> 0,10	> 0,10	
hsa-miR-423-3p	28,94	29,03	27,92	1	1,030	1,682*	> 0,10	**0,0072**	agcucggucugaggccccucagu
hsa-miR-423-5p	30,78	30,57	29,45	1	1,270	2,091*	> 0,10	**0,0104**	ugaggggcagagagcgagacuuu
hsa-miR-423-5p (duplicate)	30,66	30,70	29,53	1	1,068	1,830**	> 0,10	**0,0023**	
hsa-miR-424	24,51	25,11	23,10	1	0,720	2,197*	> 0,10	**0,0451**	cagcagcaauucauguuuugaa
hsa-miR-424*	33,08	33,50	31,43	1	0,824	2,615*	> 0,10	**0,0294**	caaaacgugaggcgcugcuau
hsa-miR-425	31,51	31,69	31,00	1	0,969	1,187	> 0,10	> 0,10	
hsa-miR-425*	32,89	33,12	32,27	1	0,942	1,282	> 0,10	> 0,10	
hsa-miR-431	35,69	36,08	35,49	1	0,820	0,936	> 0,10	> 0,10	
hsa-miR-431*	36,34	36,69	34,28	1	0,801	3,477	> 0,10	0,0632	
hsa-miR-432	36,38	36,66	35,05	1	0,908	2,094	> 0,10	> 0,10	
hsa-miR-433	35,06	35,84	34,37	1	0,677	1,328	> 0,10	> 0,10	
hsa-miR-449a	27,13	29,78	**40,00**	1	0,175	0,001**	**0,0308**	**0,0228**	uggcaguguauuguuagcuggu
hsa-miR-449b	31,83	33,98	**40,00**	1	0,247	0,003**	0,1014	**0,0082**	aggcaguguauuguuagcuggc
hsa-miR-449b*	32,72	32,97	**40,00**	1	0,811	0,005**	> 0,10	**0,0086**	cagccacaacuacccugccacu
hsa-miR-450a	32,16	32,35	31,02	1	0,963	1,829*	> 0,10	**0,0230**	uuuugcgauguguuccuaauau
hsa-miR-450b-3p	34,71	36,48	34,10	1	0,345	1,275	> 0,10	> 0,10	
hsa-miR-450b-5p	37,35	**38,41**	36,21	1	0,529	1,841	0,0519	> 0,10	uuuugcaauauguuccugaaua
hsa-miR-451	25,35	26,55	27,12	1	0,479	0,245*	> 0,10	**0,0172**	aaaccguuaccauuacugaguu
hsa-miR-452	36,17	36,27	36,37	1	1,103	0,745	> 0,10	> 0,10	
hsa-miR-454	37,19	36,77	35,88	1	1,470	2,073*	> 0,10	**0,0336**	uagugcaauauugcuuauagggu
hsa-miR-455-3p	31,80	31,47	29,04	1	1,384	5,642**	> 0,10	**0,0059**	gcaguccaugggcauauacac
hsa-miR-455-5p	35,01	34,74	32,57	1	1,328	4,531**	> 0,10	**0,0115**	uaugugccuuuggacuacaucg

hsa-miR-483-3p	34,77	34,76	34,19	1	1,109	1,253	> 0,10	> 0,10	
hsa-miR-484	30,98	31,08	30,56	1	1,032	1,121	> 0,10	> 0,10	
hsa-miR-485-3p	34,31	33,98	33,11	1	1,387	1,917	0,0543	0,1015	
hsa-miR-486-5p	31,68	32,72	33,63	1	0,522	0,210	> 0,10	**0,0189**	uccuguacugagcugccccgag
hsa-miR-487b	35,03	35,51	33,97	1	0,790	1,741	> 0,10	> 0,10	
hsa-miR-488	35,00	36,14	**40,00**	1	0,501	0,026**	0,0688	**0,0211**	uugaaaggcuauuucuugguc
hsa-miR-489	36,45	35,97	34,36	1	1,439	3,254*	> 0,10	**0,0425**	gugacaucacauauacggcagc
hsa-miR-491-5p	34,22	33,73	32,56	1	1,439	2,639	> 0,10	0,0732	
hsa-miR-493*	33,58	33,50	32,11	1	1,161	2,301	> 0,10	> 0,10	
hsa-miR-494	37,34	36,93	35,43	1	1,461	3,133	> 0,10	0,0855	
hsa-miR-495	32,71	32,11	31,06	1	1,672	2,628	> 0,10	> 0,10	
hsa-miR-497	27,84	28,05	26,60	1	0,953	1,958*	> 0,10	**0,0249**	cagcagcacacugugguuugu
hsa-miR-498	35,83	36,76	**40,00**	1	0,581	0,046**	> 0,10	**0,0065**	uuucaagccagggggcguuuuuc
hsa-miR-499-5p	35,29	35,85	35,56	1	0,748*	0,714	**0,0499**	> 0,10	uuaagacuugcagugauguuu
hsa-miR-500	32,48	32,72	31,34	1	0,935	1,844*	> 0,10	**0,0119**	uaauccuugcuaccugggugaga
hsa-miR-501-3p	33,56	33,20	32,20	1	1,416	2,142	> 0,10	> 0,10	
hsa-miR-501-5p	32,40	32,26	31,47	1	1,214	1,589	> 0,10	> 0,10	
hsa-miR-502-3p	32,55	31,85	30,68	1	1,790	3,046*	0,0746	**0,0044**	aaugcaccugggcaaggauuca
hsa-miR-503	30,50	30,53	29,01	1	1,080	2,343*	> 0,10	**0,0038**	uagcagcgggaacaguucugcag
hsa-miR-504	**40,00**	35,86	35,31	1	19,442**	19,719	**0,0069**	> 0,10	agacccuggucugcacucuauc
hsa-miR-505	34,20	33,62	32,55	1	1,649	2,609	> 0,10	> 0,10	
hsa-miR-506	31,92	31,75	30,30	1	1,236	2,565*	> 0,10	**0,0351**	uaaggcacccuucugaguaga
hsa-miR-508-3p	30,62	30,62	28,40	1	1,077	3,806*	> 0,10	**0,0327**	uacuccagagggcgucacucaug
hsa-miR-508-5p	30,85	30,87	29,35	1	1,083	2,356	> 0,10	0,0721	
hsa-miR-509-3-5p	28,78	28,88	27,37	1	1,027	2,206	> 0,10	0,0573	
hsa-miR-509-3p	28,85	28,92	27,10	1	1,049	2,803	> 0,10	0,0508	
hsa-miR-510	29,71	29,58	28,27	1	1,204	2,256	> 0,10	0,0551	
hsa-miR-511	34,37	**40,00**	**40,00**	1	0,022**	0,016**	**0,0045**	**0,0048**	gugucuuuugcucugcaguca
hsa-miR-512-5p	33,38	34,05	**40,00**	1	0,695*	0,008**	**0,0042**	**0,0002**	cacucagccuugagggcacuuuc
hsa-miR-513a-3p	30,28	30,29	28,85	1	1,093	2,244*	> 0,10	**0,0170**	uaaauuucaccuuucugagaagg
hsa-miR-513a-5p	30,45	30,53	29,07	1	1,038	2,175	> 0,10	0,0845	
hsa-miR-513c	29,29	29,34	28,06	1	1,065	1,952	> 0,10	0,1008	
hsa-miR-514	28,58	28,40	27,60	1	1,251	1,644	> 0,10	> 0,10	

hsa-miR-515-3p	35,07	35,32	**40,00**	1	0,926	0,027**	> 0,10	**0,0111**	gagugccuucuuuuggagcguu
hsa-miR-515-5p	32,28	32,42	**40,00**	1	1,001	0,004**	> 0,10	**0,0270**	uucuccaaaagaaagcacuuucug
hsa-miR-516a-3p	36,40	36,65	**40,00**	1	0,930	0,069	> 0,10	0,0600	ugcuuccuuucagagggu
hsa-miR-516a-5p	36,18	37,72	**40,00**	1	0,379	0,059**	> 0,10	**0,0042**	uucucgaggaaagaagcacuuuc
hsa-miR-516b	36,36	**40,00**	**40,00**	1	0,087	0,066	0,0995	0,0974	aucuggagguaagaagcacuuu
hsa-miR-517a	33,11	33,68	**40,00**	1	0,742	0,007**	0,0967	**0,0093**	aucgugcaucccuuuagagugu
hsa-miR-517b	31,78	32,61	**40,00**	1	0,621	0,003**	0,0645	**0,0021**	aucgugcaucccuuuagagugu
hsa-miR-517c	31,02	32,19	**39,02**	1	0,487	0,003*	0,0569	**0,0207**	aucgugcauccuuuuagagugu
hsa-miR-518a-3p	33,50	34,66	**40,00**	1	0,495	0,009**	> 0,10	**0,0325**	gaaagcgcuucccuuugcugga
hsa-miR-518a-5p	36,02	35,83	**40,00**	1	1,173	0,053**	> 0,10	**0,0298**	cugcaaagggaagcccuuuc
hsa-miR-518b	34,07	34,17	**40,00**	1	1,062	0,014*	> 0,10	**0,0331**	caaagcgcuccccuuuagaggu
hsa-miR-518c	33,18	33,42	**40,00**	1	0,929	0,007**	> 0,10	**0,0021**	caaagcgcuucucuuuagagugu
*hsa-miR-518c**	35,16	35,54	**40,00**	1	0,827	0,028	> 0,10	> 0,10	ucucuggagggaagcacuuucug
hsa-miR-518d-5p	34,10	34,59	**38,20**	1	0,731	0,052	> 0,10	0,0726	cucuagagggaagcacuuucug
hsa-miR-518e	31,55	32,69	**40,00**	1	0,498	0,002**	0,0765	**0,0104**	aaagcgcuucccuucagagug
*hsa-miR-518e**	32,44	32,76	**40,00**	1	0,819	0,004**	> 0,10	**0,0043**	cucuagagggaagcgcuuucug
hsa-miR-518f	32,80	33,52	**40,00**	1	0,665*	0,006**	**0,0349**	**0,0069**	gaaagcgcuucucuuuagagg
*hsa-miR-518f**	33,53	33,72	**38,52**	1	0,969	0,026*	> 0,10	**0,0275**	cucuagagggaagcacuuucuc
hsa-miR-519a	33,02	34,08	**40,00**	1	0,529	0,007**	0,0986	**0,0009**	aaagugcauccuuuuagagugu
hsa-miR-519b-3p	32,28	32,71	**40,00**	1	0,816	0,004**	> 0,10	**0,0275**	aaagugcauccuuuuagagguu
hsa-miR-519c-3p	33,34	34,49	**40,00**	1	0,494*	0,008**	**0,0483**	**0,0169**	aaagugcaucuuuuuagaggau
hsa-miR-519d	32,83	32,44	**40,00**	1	1,438	0,006**	> 0,10	**0,0216**	caaagugccucccuuuagagug
hsa-miR-519e	35,69	36,28	**40,00**	1	0,735	0,042**	> 0,10	**0,0336**	aagugccuccuuuuagaguguu
*hsa-miR-519e**	33,84	34,28	**40,00**	1	0,814	0,012**	> 0,10	**0,0139**	uucuccaaaagggagcacuuuc
hsa-miR-520a-3p	36,82	**38,31**	**40,00**	1	0,391	0,092**	> 0,10	**0,0494**	aaagugcuucccuuuggacugu
hsa-miR-520a-5p	33,89	34,77	**40,00**	1	0,599	0,012**	> 0,10	**0,0157**	cuccagagggaaguacuuucu
hsa-miR-520b	31,03	31,22	**40,00**	1	0,968	0,002**	> 0,10	**0,0048**	aaagugcuuccuuuuagaggg
hsa-miR-520c-3p	30,67	30,95	**40,00**	1	0,908	0,001**	> 0,10	**0,0131**	aaagugcuuccuuuuagagggu
hsa-miR-520d-5p	35,45	35,82	**40,00**	1	0,770	0,037	> 0,10	> 0,10	cuacaaagggaagcccuuuc
hsa-miR-520e	34,52	34,54	**40,00**	1	1,084	0,018	> 0,10	0,0973	aaagugcuuccuuuuugaggg
hsa-miR-520f	31,25	31,69	**40,00**	1	0,807	0,002**	> 0,10	**0,0169**	aagugcuuccuuuuagaggguu
hsa-miR-520g	32,82	33,22	**40,00**	1	0,836	0,006**	> 0,10	**0,0020**	acaaagugcuucccuuuagagugu
hsa-miR-520h	32,39	32,71	**40,00**	1	0,795	0,004**	> 0,10	**0,0480**	acaaagugcuucccuuuagagu

hsa-miR-521	36,43	**40,00**	**40,00**	1	0,093**	0,070**	**0,0147**	**0,0141**	aacgcacuucccuuuagagugu
hsa-miR-522	37,12	37,83	**40,00**	1	0,671	0,113**	> 0,10	**0,0091**	aaaaugguucccuuuagagugu
hsa-miR-523	34,38	34,73	**38,92**	1	0,866	0,036*	> 0,10	**0,0318**	gaacgcgcuucccuauagagggu
hsa-miR-524-3p	35,88	35,44	**40,00**	1	1,485	0,047	> 0,10	> 0,10	gaaggcgcuucccuuuggagu
hsa-miR-524-5p	34,46	33,78	**39,27**	1	1,766	0,030*	0,0694	**0,0462**	cuacaaagggaagcacuuucuc
hsa-miR-525-3p	34,44	34,05	37,66	1	1,444	0,090	> 0,10	> 0,10	
hsa-miR-525-5p	34,31	35,50	**40,00**	1	0,449	0,016**	> 0,10	**0,0247**	cuccagagggaugcacuuucu
hsa-miR-526b	35,48	36,20	**40,00**	1	0,669	0,036	> 0,10	0,0864	cucuugagggaagcacuuucugu
*hsa-miR-526b**	32,94	32,94	**40,00**	1	1,100	0,006**	> 0,10	**0,0024**	gaaagugcuuccuuuuagaggc
hsa-miR-532-3p	31,46	31,01	29,98	1	1,505	2,333**	0,0512	**0,0137**	ccucccacacccaaggcuugca
hsa-miR-532-5p	32,31	31,87	30,65	1	1,496	2,642*	> 0,10	**0,0013**	caugccuugaguguaggaccgu
hsa-miR-542-5p	31,79	31,91	30,79	1	1,011	1,665*	> 0,10	**0,0464**	ucggggaucaucaugucacgaga
hsa-miR-543	33,54	33,48	31,57	1	1,145	3,257	> 0,10	> 0,10	
hsa-miR-545	35,75	36,31	34,97	1	0,746	1,430	> 0,10	> 0,10	
hsa-miR-548o	**40,00**	36,82	36,69	1	9,952	8,101*	0,1027	**0,0230**	ccaaaacugcaguuacuuuugc
hsa-miR-550	36,30	36,04	**40,00**	1	1,366	0,067	> 0,10	0,0762	agugccugagggaguaagagccc
hsa-miR-550*	34,95	35,30	35,77	1	0,860	0,470	> 0,10	0,0704	
hsa-miR-551a	**40,00**	34,46	35,11	1	51,436**	26,342	**0,0092**	> 0,10	gcgacccacucuugguuucca
hsa-miR-551b	31,98	33,02	34,99	1	0,534	0,094	> 0,10	0,0573	
hsa-miR-566	35,11	36,33	35,65	1	0,498	1,000	0,0647	0,0524	
hsa-miR-574-3p	29,45	29,21	27,90	1	1,305	2,437*	> 0,10	**0,0053**	cacgcucaugcacacacccaca
hsa-miR-582-5p	36,27	36,47	33,95	1	0,962	4,181*	> 0,10	**0,0312**	uuacaguuguucaaccaguuacu
hsa-miR-584	36,28	34,65	34,26	1	2,415	2,384	> 0,10	> 0,10	
hsa-miR-590-5p	32,47	32,70	32,10	1	0,941	1,072	> 0,10	> 0,10	
hsa-miR-597	**40,00**	**40,00**	37,39	1	1,102	5,090**	> 0,10	**0,0144**	ugugucacucgaugaccacugu
hsa-miR-598	33,50	33,84	32,49	1	0,867	1,673	> 0,10	> 0,10	
hsa-miR-605	31,07	30,96	31,35	1	1,195	0,690	> 0,10	> 0,10	
hsa-miR-615-3p	34,77	35,37	34,72	1	0,717	0,878	> 0,10	> 0,10	
hsa-miR-625*	33,53	35,42	35,12	1	0,296*	0,276**	**0,0293**	**0,0351**	gacuauagaacuuucccccuca
hsa-miR-628-3p	34,34	34,96	34,84	1	0,716	0,587	> 0,10	0,0837	
hsa-miR-629	34,12	35,44	35,08	1	0,440	0,427*	0,0702	**0,0032**	uggguuuacguugggagaacu
hsa-miR-643	31,78	33,48	33,72	1	0,353	0,225*	0,0547	**0,0404**	acuuguaugcuagcucagguag
hsa-miR-651	35,55	35,43	34,62	1	1,199	1,597	> 0,10	> 0,10	

hsa-miR-652	32,61	32,93	31,58	1	0,879	1,694	> 0,10	0,0717	
hsa-miR-654-3p	32,70	33,11	31,81	1	0,826	1,541	> 0,10	> 0,10	
hsa-miR-654-5p	36,41	36,83	35,47	1	0,821	1,601	> 0,10	> 0,10	
hsa-miR-660	32,54	32,75	31,34	1	0,950	1,914	> 0,10	**0,0241**	uacccauugcauaucggaguug
hsa-miR-663	29,26	29,66	29,41	1	0,839	0,753	> 0,10	> 0,10	
hsa-miR-664	32,17	31,87	30,87	1	1,362	2,064	0,1040	0,1091	
hsa-miR-675*	34,33	34,27	33,50	1	1,105	1,539	> 0,10	> 0,10	
hsa-miR-7	33,82	33,72	**38,00**	1	1,181	0,046*	> 0,10	**0,0000**	uggaagacuagugauuuuguugu
hsa-miR-708	34,12	33,43	32,30	1	1,775	2,929	> 0,10	> 0,10	
hsa-miR-708*	36,29	35,85	35,58	1	1,574	1,271	> 0,10	> 0,10	
hsa-miR-7-1*	35,99	36,27	37,50	1	0,902	0,292**	> 0,10	**0,0036**	caacaaaucacagucugccaua
hsa-miR-720	21,91	21,70	21,79	1	1,275	0,906	> 0,10	> 0,10	
hsa-miR-744	35,37	35,40	34,09	1	1,079	2,031*	> 0,10	**0,0135**	ugcggggcuagggcuaacagca
hsa-miR-744*	34,41	34,49	33,21	1	1,018	1,994	> 0,10	> 0,10	
hsa-miR-760	33,89	33,38	33,58	1	1,537	1,008	> 0,10	> 0,10	
hsa-miR-766	31,43	31,15	30,01	1	1,344	2,241*	> 0,10	**0,0393**	acuccagccccacagccucagc
hsa-miR-769-3p	35,69	36,50	36,12	1	0,629	0,635	> 0,10	> 0,10	
hsa-miR-769-5p	32,07	32,82	32,80	1	0,656	0,503*	> 0,10	**0,0236**	ugagaccucuggguucugagcu
hsa-miR-873	33,46	34,26	32,08	1	0,632	2,158	> 0,10	> 0,10	
hsa-miR-876-3p	36,21	37,21	34,82	1	0,549	2,247	> 0,10	> 0,10	
hsa-miR-876-5p	35,87	37,76	34,30	1	0,296	2,550	> 0,10	> 0,10	
hsa-miR-885-5p	36,79	**38,27**	**40,00**	1	0,397	0,090**	**0,0278**	**0,0164**	uccauuacacuacccugccucu
hsa-miR-886-3p	30,57	30,42	28,74	1	1,222	2,966	> 0,10	0,1004	
hsa-miR-886-5p	33,81	33,83	33,81	1	1,081	0,830	> 0,10	> 0,10	
hsa-miR-887	34,77	34,48	34,14	1	1,252	1,287	> 0,10	> 0,10	
hsa-miR-888	35,88	**40,00**	**40,00**	1	0,062	0,047	> 0,10	> 0,10	uacucaaaaagcugucaguca
hsa-miR-891a	32,78	33,07	**38,60**	1	0,902	0,015	> 0,10	**0,0001**	ugcaacgaaccugagccacuga
hsa-miR-9	32,60	33,54	34,23	1	0,575	0,270	> 0,10	> 0,10	
hsa-miR-92a	27,14	27,21	26,14	1	1,051	1,670*	> 0,10	**0,0166**	uauugcacuugucccggccugu
hsa-miR-92b	32,85	33,11	32,68	1	0,922	0,934	> 0,10	> 0,10	
hsa-miR-92b*	36,21	36,48	36,40	1	0,914	0,729	> 0,10	> 0,10	
hsa-miR-93	29,67	30,02	30,14	1	0,865	0,602	> 0,10	0,0772	
hsa-miR-93*	33,83	33,74	34,10	1	1,174	0,694	> 0,10	> 0,10	

hsa-miR-940	27,13	27,43	26,95	1	0,897	0,945	> 0,10	> 0,10	
hsa-miR-941	35,39	35,54	33,99	1	0,994	2,207	> 0,10	> 0,10	
hsa-miR-95	34,13	34,37	33,86	1	0,938	1,009	> 0,10	> 0,10	
hsa-miR-96	33,08	35,49	**39,29**	1	0,207	0,011	0,0850	0,0594	uuuggcacuagcacauuuuugcu
hsa-miR-98	36,93	36,60	35,37	1	1,389	2,469	> 0,10	**0,0335**	ugagguaguaaguuguauuguu
hsa-miR-99a	26,56	26,42	25,14	1	1,212	2,241*	> 0,10	**0,0116**	aacccguagauccgaucuugug
hsa-miR-99a*	31,98	31,32	30,17	1	1,735	2,923	> 0,10	**0,0235**	caagcucgcuucuaugggucug
hsa-miR-99b	28,30	28,10	27,04	1	1,264	1,990*	0,0878	**0,0264**	cacccguagaaccgaccuugcg
hsa-miR-99b*	33,18	32,71	31,74	1	1,525	2,266*	0,1013	**0,0161**	caagcucgugucugugggguccg
hsa-miR-505*	**40,00**	36,66	35,35	1	12,882	19,181	> 0,10	> 0,10	gggagccaggaaguauugaugu
hsa-miR-629*	36,24	34,83	**40,00**	1	2,527	0,061	> 0,10	> 0,10	guucucccaacguaagcccagc
hsa-miR-661	34,76	36,17	**40,00**	1	0,357	0,022	> 0,10	> 0,10	ugccugggucucuggccugcgcgu
hsa-miR-649	36,43	36,92	**40,00**	1	0,934	0,073	> 0,10	> 0,10	aaaccuguguuguucaagaguc
hsa-miR-20b*	32,16	32,62	30,92	1	0,774	1,750	> 0,10	0,0543	
hsa-miR-767-5p	35,72	35,50	37,74	1	1,488	0,213	> 0,10	> 0,10	
hsa-miR-548b-3p	36,09	36,50	36,91	1	0,719	0,432	> 0,10	> 0,10	
hsa-miR-675b	35,23	35,96	35,31	1	0,569	0,775	> 0,10	> 0,10	
hsa-miR-338-5p	36,92	36,51	37,56	1	1,670	0,528	> 0,10	> 0,10	
hsa-miRPlus-C1089	34,41	34,53	33,61	1	1,011	1,444	> 0,10	0,1010	
hsa-miRPlus-D1033	33,54	34,30	33,56	1	0,651	0,823	> 0,10	> 0,10	
SNORD38B	24,38	24,22	23,90	1	1,226	1,166	> 0,10	> 0,10	
SNORD38B	24,20	24,07	23,78	1	1,207	1,122	> 0,10	> 0,10	
SNORD49A	25,12	24,97	24,53	1	1,224	1,258	> 0,10	> 0,10	
SNORD49A	24,79	24,79	24,23	1	1,099	1,226	> 0,10	> 0,10	
U6	21,34	21,18	20,90	1	1,230	1,132	> 0,10	> 0,10	
U6	21,34	21,18	20,90	1	1,230	1,132	> 0,10	> 0,10	

Supplemental Table S2. Pearson correlation coefficients (r) and adjusted p-values (p) between the molecular and histological parameters for the nine samples analysed in miRNA profiling. Significant differences are indicated in bold.

		Sertoli cell	Spermatogonia	Spermatocyte I	Round sp	Elongated sp	TESE value	Johnsen Score
hsa_let_7a	Pearson correlation coefficient (r)	**0,743**	**-0,958**	**-0,834**	-0,454	-0,382	-0,400	**-0,730**
	Adjusted p-value	**0,022**	**0,000**	**0,005**	0,220	0,310	0,287	**0,026**
hsa_let_7a#	Pearson correlation coefficient (r)	0,433	**-0,752**	**-0,715**	-0,586	-0,513	-0,525	**-0,768**
	Adjusted p-value	0,245	**0,019**	**0,030**	0,097	0,158	0,146	**0,016**
hsa_let_7a_2#	Pearson correlation coefficient (r)	-0,054	0,075	-0,064	-0,376	-0,445	-0,407	-0,285
	Adjusted p-value	0,890	0,849	0,869	0,319	0,230	0,277	0,458
hsa_let_7b	Pearson correlation coefficient (r)	**0,763**	**-0,941**	**-0,918**	-0,533	-0,484	-0,501	**-0,794**
	Adjusted p-value	**0,017**	**0,000**	**0,000**	0,140	0,186	0,170	**0,011**
hsa_let_7b#	Pearson correlation coefficient (r)	**0,828**	**-0,742**	**-0,807**	**-0,745**	**-0,719**	**-0,703**	**-0,912**
	Adjusted p-value	**0,006**	**0,022**	**0,009**	**0,021**	**0,029**	**0,035**	**0,001**
hsa_let_7c	Pearson correlation coefficient (r)	**0,768**	**-0,944**	**-0,873**	-0,469	-0,410	-0,424	**-0,746**
	Adjusted p-value	**0,016**	**0,000**	**0,002**	0,203	0,273	0,256	**0,021**
hsa_let_7d	Pearson correlation coefficient (r)	**0,747**	**-0,972**	**-0,797**	-0,397	-0,298	-0,330	**-0,682**
	Adjusted p-value	**0,021**	**0,000**	**0,010**	0,290	0,436	0,386	**0,043**
hsa_let_7d#	Pearson correlation coefficient (r)	-0,253	0,466	0,150	0,013	-0,072	-0,043	0,099
	Adjusted p-value	0,512	0,206	0,700	0,974	0,854	0,914	0,801
hsa_let_7e	Pearson correlation coefficient (r)	**0,830**	**-0,927**	**-0,837**	-0,628	-0,546	-0,576	**-0,842**
	Adjusted p-value	**0,006**	**0,000**	**0,005**	0,070	0,128	0,105	**0,004**
hsa_let_7f	Pearson correlation coefficient (r)	**0,696**	**-0,793**	-0,590	-0,515	-0,390	-0,426	**-0,675**
	Adjusted p-value	**0,037**	**0,011**	0,094	0,156	0,299	0,252	**0,046**
hsa_let_7f_1#	Pearson correlation coefficient (r)	**0,871**	**-0,814**	**-0,875**	-0,484	-0,522	-0,490	**-0,727**
	Adjusted p-value	**0,002**	**0,008**	**0,002**	0,186	0,150	0,181	**0,026**
hsa_let_7f_2#	Pearson correlation coefficient (r)	**0,749**	**-0,783**	**-0,690**	-0,271	-0,236	-0,226	-0,520
	Adjusted p-value	**0,020**	**0,013**	**0,040**	0,481	0,541	0,559	0,151
hsa_let_7g	Pearson correlation coefficient (r)	**0,855**	**-0,951**	**-0,834**	-0,602	-0,527	-0,548	**-0,827**
	Adjusted p-value	**0,003**	**0,000**	**0,005**	0,087	0,145	0,127	**0,006**
hsa_let_7g#	Pearson correlation coefficient (r)	**0,798**	**-0,883**	**-0,693**	-0,493	-0,432	-0,422	**-0,736**
	Adjusted p-value	**0,010**	**0,002**	**0,038**	0,177	0,246	0,258	**0,024**
hsa_let_7i	Pearson correlation coefficient (r)	**0,701**	**-0,922**	**-0,842**	-0,497	-0,432	-0,452	**-0,749**

	Adjusted p-value	**0,035**	**0,000**	**0,004**	0,173	0,246	0,222	**0,020**
hsa_let_7i#	Pearson correlation coefficient (r)	0,429	**-0,709**	-0,667	-0,656	-0,598	-0,619	**-0,744**
	Adjusted p-value	0,249	**0,033**	0,050	0,055	0,089	0,075	**0,022**
hsa_miR_1	Pearson correlation coefficient (r)	-0,210	0,208	0,071	-0,390	-0,362	-0,371	-0,203
	Adjusted p-value	0,587	0,592	0,855	0,300	0,338	0,326	0,601
hsa_miR_100	Pearson correlation coefficient (r)	0,659	**-0,879**	**-0,885**	-0,648	-0,595	-0,611	**-0,851**
	Adjusted p-value	0,054	**0,002**	**0,002**	0,059	0,091	0,081	**0,004**
hsa_miR_101	Pearson correlation coefficient (r)	0,428	-0,666	-0,510	-0,454	-0,336	-0,375	-0,579
	Adjusted p-value	0,250	0,050	0,160	0,220	0,377	0,320	0,103
hsa_miR_101#	Pearson correlation coefficient (r)	0,292	-0,526	-0,567	-0,353	-0,299	-0,342	-0,471
	Adjusted p-value	0,445	0,146	0,111	0,351	0,434	0,368	0,201
hsa_miR_103	Pearson correlation coefficient (r)	**0,848**	**-0,914**	**-0,794**	-0,578	-0,483	-0,508	**-0,801**
	Adjusted p-value	**0,004**	**0,001**	**0,011**	0,103	0,187	0,162	**0,009**
hsa_miR_103_dup	Pearson correlation coefficient (r)	**0,783**	**-0,907**	**-0,796**	-0,527	-0,420	-0,451	**-0,770**
	Adjusted p-value	**0,013**	**0,001**	**0,010**	0,145	0,261	0,223	**0,015**
hsa_miR_103_2#	Pearson correlation coefficient (r)	0,642	**-0,931**	**-0,906**	-0,430	-0,387	-0,395	**-0,735**
	Adjusted p-value	0,086	**0,001**	**0,002**	0,287	0,343	0,332	**0,038**
hsa_miR_103_as	Pearson correlation coefficient (r)	**0,880**	**-0,783**	**-0,688**	-0,318	-0,249	-0,269	-0,564
	Adjusted p-value	**0,002**	**0,013**	**0,040**	0,404	0,519	0,484	0,114
hsa_miR_105	Pearson correlation coefficient (r)	**-0,755**	**0,886**	**0,820**	0,644	0,597	0,603	**0,827**
	Adjusted p-value	**0,019**	**0,001**	**0,007**	0,061	0,089	0,086	**0,006**
hsa_miR_106a	Pearson correlation coefficient (r)	**0,712**	**-0,747**	-0,480	-0,301	-0,190	-0,210	-0,529
	Adjusted p-value	**0,032**	**0,021**	0,191	0,431	0,624	0,588	0,143
hsa_miR_106b	Pearson correlation coefficient (r)	0,262	-0,224	0,052	0,067	0,174	0,159	0,005
	Adjusted p-value	0,496	0,563	0,895	0,864	0,654	0,683	0,990
hsa_miR_106b#	Pearson correlation coefficient (r)	-0,600	0,660	**0,851**	**0,721**	**0,692**	**0,718**	**0,829**
	Adjusted p-value	0,087	0,053	**0,004**	**0,028**	**0,039**	**0,029**	**0,006**
hsa_miR_107	Pearson correlation coefficient (r)	**0,785**	**-0,899**	**-0,861**	-0,548	-0,479	-0,491	**-0,810**
	Adjusted p-value	**0,012**	**0,001**	**0,003**	0,127	0,192	0,180	**0,008**
hsa_miR_10a	Pearson correlation coefficient (r)	-0,319	0,620	**0,872**	0,201	0,268	0,252	0,458
	Adjusted p-value	0,403	0,075	**0,002**	0,604	0,485	0,513	0,215
hsa_miR_10b	Pearson correlation coefficient (r)	0,542	-0,586	**-0,680**	-0,616	-0,535	-0,574	**-0,723**
	Adjusted p-value	0,132	0,097	**0,044**	0,078	0,138	0,106	**0,028**

hsa_miR_10b#	Pearson correlation coefficient (r)	**0,723**	-0,639	**-0,677**	**-0,912**	**-0,879**	**-0,884**	**-0,944**
	Adjusted p-value	**0,028**	0,064	**0,045**	**0,001**	**0,002**	**0,002**	**0,000**
hsa_miR_1185	Pearson correlation coefficient (r)	0,576	**-0,841**	-0,589	-0,358	-0,253	-0,275	-0,595
	Adjusted p-value	0,104	**0,004**	0,095	0,343	0,511	0,474	0,091
hsa_miR_1201	Pearson correlation coefficient (r)	-0,110	0,276	0,495	0,389	0,455	0,422	0,420
	Adjusted p-value	0,777	0,472	0,176	0,300	0,219	0,258	0,261
hsa_miR_122	Pearson correlation coefficient (r)	-0,546	0,370	0,585	**0,942**	**0,975**	**0,948**	**0,905**
	Adjusted p-value	0,129	0,327	0,098	**0,000**	**0,000**	**0,000**	**0,001**
hsa_miR_124	Pearson correlation coefficient (r)	-0,373	0,504	**0,777**	**0,888**	**0,880**	**0,904**	**0,910**
	Adjusted p-value	0,362	0,203	**0,023**	**0,003**	**0,004**	**0,002**	**0,002**
hsa_miR_1247	Pearson correlation coefficient (r)	0,495	**-0,787**	**-0,815**	-0,426	-0,385	-0,404	-0,664
	Adjusted p-value	0,175	**0,012**	**0,007**	0,252	0,306	0,281	0,051
hsa_miR_1248	Pearson correlation coefficient (r)	-0,004	0,489	0,211	-0,184	-0,274	-0,222	-0,006
	Adjusted p-value	0,991	0,182	0,587	0,635	0,476	0,565	0,988
hsa_miR_125a_3p	Pearson correlation coefficient (r)	0,412	-0,542	-0,614	-0,492	-0,485	-0,485	-0,568
	Adjusted p-value	0,270	0,132	0,079	0,178	0,186	0,186	0,111
hsa_miR_125a_5p	Pearson correlation coefficient (r)	**0,731**	**-0,910**	**-0,875**	-0,662	-0,601	-0,624	**-0,870**
	Adjusted p-value	**0,025**	**0,001**	**0,002**	0,052	0,087	0,072	**0,002**
hsa_miR_125b	Pearson correlation coefficient (r)	**0,741**	**-0,920**	**-0,927**	-0,652	-0,601	-0,613	**-0,885**
	Adjusted p-value	**0,022**	**0,000**	**0,000**	0,057	0,087	0,079	**0,002**
hsa_miR_125b_1#	Pearson correlation coefficient (r)	0,658	**-0,838**	**-0,790**	-0,605	-0,534	-0,565	**-0,802**
	Adjusted p-value	0,054	**0,005**	**0,011**	0,084	0,139	0,113	**0,009**
hsa_miR_125b_2#	Pearson correlation coefficient (r)	0,641	**-0,833**	**-0,914**	-0,564	-0,538	-0,556	**-0,766**
	Adjusted p-value	0,063	**0,005**	**0,001**	0,114	0,135	0,120	**0,016**
hsa_miR_126	Pearson correlation coefficient (r)	-0,277	-0,130	-0,056	-0,070	0,050	-0,031	-0,066
	Adjusted p-value	0,470	0,738	0,886	0,858	0,898	0,937	0,867
hsa_miR_126#	Pearson correlation coefficient (r)	-0,221	-0,200	-0,040	-0,053	0,080	-0,002	-0,055
	Adjusted p-value	0,567	0,606	0,919	0,891	0,837	0,996	0,888
hsa_miR_1260	Pearson correlation coefficient (r)	-0,347	0,499	0,542	0,506	0,397	0,484	0,557
	Adjusted p-value	0,360	0,171	0,132	0,165	0,290	0,187	0,119
hsa_miR_1270	Pearson correlation coefficient (r)	**0,927**	**-0,840**	**-0,808**	-0,489	-0,456	-0,460	**-0,717**
	Adjusted p-value	**0,000**	**0,005**	**0,008**	0,182	0,217	0,213	**0,030**
hsa_miR_1271	Pearson correlation coefficient (r)	0,110	-0,449	**-0,848**	-0,709	-0,708	-0,712	**-0,834**

	Adjusted p-value	0,814	0,312	**0,016**	0,075	0,075	0,072	**0,020**
hsa_miR_127_3p	Pearson correlation coefficient (r)	0,613	**-0,818**	**-0,760**	-0,453	-0,369	-0,402	**-0,679**
	Adjusted p-value	0,079	**0,007**	**0,018**	0,221	0,329	0,284	**0,044**
hsa_miR_127_5p	Pearson correlation coefficient (r)	0,450	**-0,740**	-0,621	-0,120	-0,042	-0,063	-0,400
	Adjusted p-value	0,225	**0,023**	0,074	0,759	0,915	0,871	0,286
hsa_miR_129#	Pearson correlation coefficient (r)	**0,767**	**-0,762**	**-0,736**	-0,413	-0,386	-0,388	-0,629
	Adjusted p-value	**0,016**	**0,017**	**0,024**	0,269	0,305	0,302	0,070
hsa_miR_129_3p	Pearson correlation coefficient (r)	0,574	-0,613	-0,640	-0,469	-0,456	-0,441	-0,635
	Adjusted p-value	0,106	0,079	0,063	0,203	0,218	0,235	0,066
hsa_miR_129_5p	Pearson correlation coefficient (r)	**0,692**	**-0,728**	**-0,705**	-0,372	-0,347	-0,347	-0,589
	Adjusted p-value	**0,039**	**0,026**	**0,034**	0,324	0,361	0,361	0,095
hsa_miR_1296	Pearson correlation coefficient (r)	-0,639	**0,891**	**0,856**	0,336	0,301	0,294	0,660
	Adjusted p-value	0,064	**0,001**	**0,003**	0,377	0,431	0,442	0,053
hsa_miR_130a	Pearson correlation coefficient (r)	0,446	**-0,710**	**-0,697**	-0,546	-0,464	-0,494	**-0,703**
	Adjusted p-value	0,229	**0,032**	**0,037**	0,128	0,209	0,177	**0,035**
hsa_miR_130b	Pearson correlation coefficient (r)	-0,206	0,029	0,073	-0,209	-0,050	-0,136	-0,114
	Adjusted p-value	0,595	0,941	0,853	0,589	0,898	0,728	0,770
hsa_miR_130b#	Pearson correlation coefficient (r)	-0,695	0,585	0,363	0,352	0,266	0,283	0,508
	Adjusted p-value	0,055	0,128	0,377	0,392	0,525	0,498	0,199
hsa_miR_132	Pearson correlation coefficient (r)	-0,139	-0,331	-0,167	-0,016	0,068	0,037	-0,110
	Adjusted p-value	0,722	0,384	0,667	0,968	0,861	0,925	0,779
hsa_miR_132#	Pearson correlation coefficient (r)	-0,391	-0,019	0,055	0,623	0,615	0,608	0,460
	Adjusted p-value	0,299	0,960	0,889	0,073	0,078	0,082	0,213
hsa_miR_133a	Pearson correlation coefficient (r)	-0,281	0,229	-0,222	-0,448	-0,497	-0,495	-0,329
	Adjusted p-value	0,464	0,553	0,566	0,227	0,173	0,175	0,388
hsa_miR_133b	Pearson correlation coefficient (r)	-0,404	0,328	-0,055	-0,485	-0,500	-0,519	-0,272
	Adjusted p-value	0,281	0,389	0,889	0,186	0,171	0,152	0,479
hsa_miR_134	Pearson correlation coefficient (r)	0,452	**-0,683**	**-0,692**	-0,596	-0,497	-0,547	**-0,725**
	Adjusted p-value	0,222	**0,043**	**0,039**	0,091	0,174	0,128	**0,027**
hsa_miR_135a	Pearson correlation coefficient (r)	**0,945**	**-0,803**	**-0,711**	-0,463	-0,412	-0,408	**-0,691**
	Adjusted p-value	**0,000**	**0,009**	**0,032**	0,210	0,271	0,276	**0,039**
hsa_miR_135a#	Pearson correlation coefficient (r)	0,061	-0,141	-0,004	0,119	0,142	0,164	-0,022
	Adjusted p-value	0,886	0,739	0,993	0,779	0,738	0,697	0,959

hsa_miR_135b	Pearson correlation coefficient (r)	**0,814**	**-0,917**	**-0,893**	-0,491	-0,463	-0,462	-0,764
	Adjusted p-value	**0,008**	**0,001**	**0,001**	0,179	0,209	0,210	0,017
hsa_miR_136	Pearson correlation coefficient (r)	0,558	**-0,748**	-0,648	-0,350	-0,278	-0,298	-0,569
	Adjusted p-value	0,119	**0,020**	0,059	0,355	0,469	0,436	0,110
hsa_miR_138	Pearson correlation coefficient (r)	-0,658	**0,772**	**0,673**	0,413	0,410	0,388	0,599
	Adjusted p-value	0,054	**0,015**	**0,047**	0,270	0,273	0,302	0,088
hsa_miR_139_5p	Pearson correlation coefficient (r)	0,142	-0,606	-0,453	-0,010	0,105	0,038	-0,225
	Adjusted p-value	0,716	0,084	0,221	0,980	0,789	0,923	0,561
hsa_miR_140_3p	Pearson correlation coefficient (r)	0,607	**-0,857**	**-0,881**	-0,645	-0,587	-0,599	**-0,849**
	Adjusted p-value	0,083	**0,003**	**0,002**	0,061	0,097	0,088	**0,004**
hsa_miR_140_5p	Pearson correlation coefficient (r)	0,613	**-0,821**	-0,638	-0,521	-0,460	-0,453	**-0,722**
	Adjusted p-value	0,106	**0,013**	0,089	0,186	0,251	0,260	**0,043**
hsa_miR_141	Pearson correlation coefficient (r)	0,407	-0,152	0,381	0,341	0,450	0,437	0,274
	Adjusted p-value	0,365	0,745	0,398	0,455	0,311	0,326	0,551
hsa_miR_142_3p	Pearson correlation coefficient (r)	0,499	-0,540	-0,477	-0,551	-0,502	-0,515	-0,605
	Adjusted p-value	0,171	0,133	0,194	0,125	0,168	0,156	0,085
hsa_miR_142_5p	Pearson correlation coefficient (r)	0,430	-0,476	-0,487	-0,647	-0,626	-0,615	**-0,682**
	Adjusted p-value	0,248	0,195	0,184	0,060	0,071	0,078	**0,043**
hsa_miR_143	Pearson correlation coefficient (r)	0,134	-0,345	-0,398	-0,599	-0,549	-0,566	-0,565
	Adjusted p-value	0,731	0,363	0,289	0,088	0,126	0,112	0,113
hsa_miR_143#	Pearson correlation coefficient (r)	0,004	-0,167	-0,177	-0,535	-0,470	-0,493	-0,432
	Adjusted p-value	0,993	0,667	0,649	0,138	0,202	0,178	0,246
hsa_miR_144	Pearson correlation coefficient (r)	-0,509	0,395	0,422	0,265	0,285	0,270	0,418
	Adjusted p-value	0,161	0,293	0,258	0,491	0,457	0,482	0,263
hsa_miR_144#	Pearson correlation coefficient (r)	-0,479	0,310	0,479	0,425	0,533	0,468	0,526
	Adjusted p-value	0,192	0,417	0,192	0,255	0,140	0,204	0,146
hsa_miR_145	Pearson correlation coefficient (r)	0,199	-0,554	-0,647	**-0,701**	-0,651	**-0,674**	**-0,749**
	Adjusted p-value	0,607	0,122	0,060	**0,035**	0,058	**0,046**	**0,020**
hsa_miR_145#	Pearson correlation coefficient (r)	0,418	-0,651	-0,608	-0,580	-0,513	-0,534	**-0,670**
	Adjusted p-value	0,263	0,058	0,082	0,102	0,157	0,138	**0,048**
hsa_miR_1468	Pearson correlation coefficient (r)	0,339	**-0,639**	-0,363	-0,253	-0,084	-0,159	-0,370
	Adjusted p-value	0,411	**0,088**	0,377	0,546	0,843	0,707	0,368
hsa_miR_146a	Pearson correlation coefficient (r)	**0,724**	**-0,805**	**-0,767**	-0,570	-0,536	-0,537	**-0,769**

	Adjusted p-value	**0,027**	**0,009**	**0,016**	0,109	0,137	0,136	**0,015**
hsa_miR_146b_3p	Pearson correlation coefficient (r)	0,009	-0,068	0,131	0,392	0,427	0,378	0,370
	Adjusted p-value	0,984	0,872	0,757	0,337	0,291	0,355	0,367
hsa_miR_146b_5p	Pearson correlation coefficient (r)	**0,817**	**-0,761**	-0,585	-0,450	-0,366	-0,389	-0,646
	Adjusted p-value	**0,007**	**0,017**	0,098	0,224	0,332	0,300	0,060
hsa_miR_147b	Pearson correlation coefficient (r)	-0,517	0,762	**0,946**	0,718	0,717	0,711	**0,887**
	Adjusted p-value	0,294	0,078	**0,004**	0,108	0,109	0,113	**0,018**
hsa_miR_148a	Pearson correlation coefficient (r)	**0,877**	**-0,858**	**-0,834**	-0,637	-0,605	-0,584	**-0,841**
	Adjusted p-value	**0,004**	**0,006**	**0,010**	0,089	0,112	0,129	**0,009**
hsa_miR_148a#	Pearson correlation coefficient (r)	0,101	-0,054	-0,386	**-0,822**	**-0,842**	**-0,845**	-0,685
	Adjusted p-value	0,813	0,900	0,345	**0,012**	**0,009**	**0,008**	0,061
hsa_miR_148b	Pearson correlation coefficient (r)	**0,673**	**-0,863**	**-0,760**	-0,479	-0,400	-0,421	**-0,702**
	Adjusted p-value	**0,047**	**0,003**	**0,017**	0,192	0,285	0,260	**0,035**
hsa_miR_149	Pearson correlation coefficient (r)	0,620	-0,571	-0,697	-0,630	-0,619	-0,627	**-0,758**
	Adjusted p-value	0,075	0,108	0,037	0,069	0,075	0,071	**0,018**
hsa_miR_149#	Pearson correlation coefficient (r)	-0,435	0,591	0,620	0,414	0,398	0,376	0,568
	Adjusted p-value	0,242	0,094	0,075	0,268	0,289	0,319	0,110
hsa_miR_150	Pearson correlation coefficient (r)	-0,265	0,052	-0,168	-0,435	-0,452	-0,476	-0,324
	Adjusted p-value	0,491	0,895	0,665	0,242	0,222	0,195	0,395
hsa_miR_151_3p	Pearson correlation coefficient (r)	**0,836**	**-0,869**	**-0,796**	-0,484	-0,477	-0,450	**-0,744**
	Adjusted p-value	**0,005**	**0,002**	**0,010**	0,187	0,195	0,225	**0,022**
hsa_miR_151_5p	Pearson correlation coefficient (r)	**0,743**	**-0,868**	**-0,812**	-0,525	-0,495	-0,491	**-0,764**
	Adjusted p-value	**0,022**	**0,002**	**0,008**	0,147	0,176	0,179	**0,017**
hsa_miR_152	Pearson correlation coefficient (r)	0,288	-0,655	-0,320	-0,068	0,093	0,029	-0,247
	Adjusted p-value	0,452	0,055	0,402	0,863	0,812	0,941	0,522
hsa_miR_153	Pearson correlation coefficient (r)	-0,341	0,203	0,609	0,514	0,665	0,588	0,567
	Adjusted p-value	0,369	0,600	0,082	0,157	0,051	0,096	0,111
hsa_miR_1537	Pearson correlation coefficient (r)	0,104	-0,491	-0,147	0,461	0,575	0,544	0,174
	Adjusted p-value	0,789	0,179	0,705	0,211	0,105	0,130	0,655
hsa_miR_154	Pearson correlation coefficient (r)	0,644	**-0,845**	**-0,788**	-0,491	-0,412	-0,438	**-0,718**
	Adjusted p-value	0,061	**0,004**	**0,012**	0,180	0,271	0,239	**0,029**
hsa_miR_154#	Pearson correlation coefficient (r)	0,583	-0,471	-0,424	-0,204	-0,128	-0,171	-0,330
	Adjusted p-value	0,100	0,201	0,255	0,598	0,742	0,660	0,385

hsa_miR_155	Pearson correlation coefficient (r)	0,192	-0,394	-0,130	-0,149	-0,122	-0,120	-0,199
	Adjusted p-value	0,620	0,294	0,739	0,703	0,754	0,758	0,609
hsa_miR_15a	Pearson correlation coefficient (r)	0,378	-0,655	-0,344	-0,212	-0,036	-0,100	-0,359
	Adjusted p-value	0,315	0,055	0,364	0,583	0,928	0,797	0,343
hsa_miR_15a#	Pearson correlation coefficient (r)	0,232	-0,579	-0,634	0,331	0,338	0,343	-0,031
	Adjusted p-value	0,658	0,228	0,177	0,521	0,512	0,505	0,953
hsa_miR_15b	Pearson correlation coefficient (r)	-0,287	0,312	0,631	**0,779**	**0,833**	**0,805**	**0,766**
	Adjusted p-value	0,453	0,414	0,068	**0,013**	**0,005**	**0,009**	**0,016**
hsa_miR_15b#	Pearson correlation coefficient (r)	0,506	0,059	0,261	-0,062	-0,026	0,003	-0,050
	Adjusted p-value	0,201	0,889	0,533	0,883	0,951	0,995	0,907
hsa_miR_16	Pearson correlation coefficient (r)	-0,441	0,420	0,703	0,483	0,614	0,559	0,602
	Adjusted p-value	0,235	0,260	0,035	0,187	0,079	0,118	0,086
hsa_miR_16_2#	Pearson correlation coefficient (r)	-0,651	0,590	**0,825**	**0,856**	**0,885**	**0,879**	**0,924**
	Adjusted p-value	0,057	0,094	**0,006**	**0,003**	**0,002**	**0,002**	**0,000**
hsa_miR_17	Pearson correlation coefficient (r)	**0,703**	**-0,786**	-0,570	-0,119	-0,066	-0,061	-0,415
	Adjusted p-value	**0,035**	**0,012**	0,109	0,761	0,866	0,875	0,267
hsa_miR_17#	Pearson correlation coefficient (r)	**0,832**	**-0,850**	**-0,742**	-0,504	-0,488	-0,455	**-0,752**
	Adjusted p-value	**0,005**	**0,004**	**0,022**	0,167	0,183	0,218	**0,019**
hsa_miR_181a	Pearson correlation coefficient (r)	**0,874**	**-0,902**	**-0,887**	-0,595	-0,550	-0,558	**-0,834**
	Adjusted p-value	**0,002**	**0,001**	**0,001**	0,091	0,125	0,118	**0,005**
hsa_miR_181a#	Pearson correlation coefficient (r)	0,471	-0,408	-0,623	-0,460	-0,436	-0,461	-0,575
	Adjusted p-value	0,200	0,275	0,073	0,213	0,241	0,211	0,105
hsa_miR_181a_2#	Pearson correlation coefficient (r)	0,651	-0,643	-0,685	-0,434	-0,416	-0,435	-0,567
	Adjusted p-value	0,080	0,086	0,061	0,282	0,305	0,281	0,142
hsa_miR_181b	Pearson correlation coefficient (r)	**0,804**	**-0,884**	**-0,916**	-0,542	-0,505	-0,513	**-0,803**
	Adjusted p-value	**0,009**	**0,002**	**0,001**	0,132	0,166	0,158	**0,009**
hsa_miR_181c	Pearson correlation coefficient (r)	-0,103	0,323	0,019	-0,456	-0,483	-0,454	-0,307
	Adjusted p-value	0,791	0,397	0,962	0,218	0,188	0,220	0,421
hsa_miR_181c#	Pearson correlation coefficient (r)	-0,652	**0,760**	0,492	0,075	-0,017	-0,018	0,389
	Adjusted p-value	0,057	**0,017**	0,179	0,847	0,966	0,963	0,300
hsa_miR_181d	Pearson correlation coefficient (r)	**0,834**	**-0,707**	**-0,746**	-0,551	-0,531	-0,524	**-0,720**
	Adjusted p-value	**0,005**	**0,033**	**0,021**	0,124	0,142	0,147	**0,029**
hsa_miR_182	Pearson correlation coefficient (r)	-0,490	0,408	0,714	**0,919**	**0,970**	**0,951**	**0,908**

	Adjusted p-value	0,180	0,276	0,031	**0,000**	**0,000**	**0,000**	**0,001**
hsa_miR_182#	Pearson correlation coefficient (r)	-0,569	0,468	0,538	**0,884**	0,861	0,835	**0,955**
	Adjusted p-value	0,317	0,426	0,349	**0,047**	0,061	0,078	**0,011**
hsa_miR_185	Pearson correlation coefficient (r)	-0,194	0,091	0,198	0,103	0,144	0,143	0,159
	Adjusted p-value	0,617	0,817	0,610	0,792	0,711	0,714	0,682
hsa_miR_186	Pearson correlation coefficient (r)	**0,684**	**-0,921**	**-0,932**	-0,598	-0,560	-0,564	**-0,838**
	Adjusted p-value	**0,042**	**0,000**	**0,000**	0,089	0,117	0,114	**0,005**
hsa_miR_187	Pearson correlation coefficient (r)	**0,744**	**-0,804**	**-0,803**	-0,441	-0,415	-0,411	**-0,694**
	Adjusted p-value	**0,022**	**0,009**	**0,009**	0,235	0,266	0,272	**0,038**
hsa_miR_187#	Pearson correlation coefficient (r)	0,140	-0,277	-0,201	-0,213	-0,195	-0,201	-0,238
	Adjusted p-value	0,765	0,548	0,665	0,647	0,675	0,665	0,607
hsa_miR_188_5p	Pearson correlation coefficient (r)	0,177	-0,221	-0,607	-0,427	-0,457	-0,473	-0,480
	Adjusted p-value	0,648	0,567	0,083	0,252	0,217	0,198	0,191
hsa_miR_18a	Pearson correlation coefficient (r)	-0,434	**0,718**	**0,716**	0,458	0,413	0,393	0,691
	Adjusted p-value	0,283	**0,045**	**0,046**	0,254	0,309	0,336	0,058
hsa_miR_18a#	Pearson correlation coefficient (r)	0,368	0,170	0,106	0,087	0,010	0,060	0,071
	Adjusted p-value	0,330	0,662	0,786	0,824	0,980	0,879	0,856
hsa_miR_18b	Pearson correlation coefficient (r)	-0,294	0,314	0,666	0,427	0,553	0,479	0,543
	Adjusted p-value	0,442	0,410	0,050	0,252	0,123	0,192	0,131
hsa_miR_190	Pearson correlation coefficient (r)	**0,740**	**-0,965**	**-0,768**	-0,258	-0,206	-0,218	-0,555
	Adjusted p-value	**0,023**	**0,000**	**0,016**	0,503	0,594	0,574	0,121
hsa_miR_1908	Pearson correlation coefficient (r)	0,507	**-0,720**	-0,687	-0,399	-0,403	-0,379	-0,584
	Adjusted p-value	0,199	**0,044**	0,060	0,327	0,322	0,355	0,128
hsa_miR_191	Pearson correlation coefficient (r)	**0,852**	**-0,850**	-0,551	-0,153	-0,059	-0,074	-0,435
	Adjusted p-value	**0,004**	**0,004**	0,124	0,695	0,880	0,849	0,242
hsa_miR_191_dup	Pearson correlation coefficient (r)	**0,799**	**-0,854**	-0,537	-0,348	-0,243	-0,261	-0,580
	Adjusted p-value	**0,010**	**0,003**	0,136	0,359	0,528	0,498	0,102
hsa_miR_191#	Pearson correlation coefficient (r)	-0,470	**0,754**	**0,826**	0,049	0,107	0,080	0,380
	Adjusted p-value	0,202	**0,019**	**0,006**	0,900	0,783	0,838	0,313
hsa_miR_1911#	Pearson correlation coefficient (r)	**0,890**	**-0,719**	**-0,710**	-0,409	-0,382	-0,387	-0,610
	Adjusted p-value	**0,001**	**0,029**	**0,032**	0,274	0,310	0,304	0,081
hsa_miR_192	Pearson correlation coefficient (r)	-0,606	**0,672**	**0,764**	0,343	0,438	0,380	0,577
	Adjusted p-value	0,084	**0,047**	**0,017**	0,366	0,238	0,313	0,104

hsa_miR_193a_3p	Pearson correlation coefficient (r)	0,489	**-0,696**	**-0,742**	-0,469	-0,429	-0,433	-0,667
	Adjusted p-value	0,182	**0,037**	**0,022**	0,202	0,249	0,244	0,050
hsa_miR_193a_5p	Pearson correlation coefficient (r)	0,506	**-0,769**	**-0,713**	-0,512	-0,419	-0,456	**-0,685**
	Adjusted p-value	0,165	**0,016**	**0,031**	0,158	0,262	0,218	**0,042**
hsa_miR_193b	Pearson correlation coefficient (r)	**0,895**	**-0,930**	**-0,885**	-0,557	-0,503	-0,513	**-0,812**
	Adjusted p-value	**0,001**	**0,000**	**0,002**	0,119	0,167	0,158	**0,008**
hsa_miR_193b#	Pearson correlation coefficient (r)	**0,709**	**-0,870**	**-0,899**	-0,381	-0,357	-0,340	**-0,722**
	Adjusted p-value	**0,049**	**0,005**	**0,002**	0,352	0,386	0,409	**0,043**
hsa_miR_194	Pearson correlation coefficient (r)	-0,407	0,398	0,599	**0,781**	**0,839**	**0,828**	**0,750**
	Adjusted p-value	0,278	0,289	0,088	**0,013**	**0,005**	**0,006**	**0,020**
hsa_miR_195	Pearson correlation coefficient (r)	**0,709**	**-0,842**	**-0,685**	-0,430	-0,315	-0,354	-0,645
	Adjusted p-value	**0,032**	**0,004**	**0,042**	0,248	0,409	0,350	0,061
hsa_miR_195#	Pearson correlation coefficient (r)	0,285	-0,578	-0,698	-0,549	-0,457	-0,507	-0,658
	Adjusted p-value	0,495	0,133	0,054	0,159	0,255	0,200	0,076
hsa_miR_196a	Pearson correlation coefficient (r)	-0,255	0,295	0,215	-0,314	-0,291	-0,292	-0,100
	Adjusted p-value	0,507	0,441	0,579	0,411	0,448	0,446	0,797
hsa_miR_196b	Pearson correlation coefficient (r)	-0,273	0,325	0,142	-0,365	-0,352	-0,354	-0,164
	Adjusted p-value	0,554	0,477	0,761	0,420	0,438	0,436	0,725
hsa_miR_197	Pearson correlation coefficient (r)	**0,831**	**-0,885**	**-0,836**	-0,620	-0,584	-0,592	**-0,828**
	Adjusted p-value	**0,006**	**0,002**	**0,005**	0,075	0,099	0,093	**0,006**
hsa_miR_1974	Pearson correlation coefficient (r)	0,139	-0,072	0,073	0,273	0,288	0,330	0,099
	Adjusted p-value	0,721	0,853	0,852	0,477	0,453	0,385	0,801
hsa_miR_1979	Pearson correlation coefficient (r)	0,593	**-0,844**	**-0,701**	-0,450	-0,365	-0,385	**-0,691**
	Adjusted p-value	0,092	**0,004**	**0,035**	0,224	0,335	0,306	**0,039**
hsa_miR_199a_3p	Pearson correlation coefficient (r)	0,555	**-0,835**	**-0,796**	-0,477	-0,415	-0,436	**-0,699**
	Adjusted p-value	0,121	**0,005**	**0,010**	0,195	0,266	0,240	**0,036**
hsa_miR_199a_5p	Pearson correlation coefficient (r)	0,517	**-0,791**	**-0,818**	-0,595	-0,540	-0,558	**-0,785**
	Adjusted p-value	0,154	**0,011**	**0,007**	0,091	0,134	0,118	**0,012**
hsa_miR_199b_5p	Pearson correlation coefficient (r)	0,356	**-0,710**	-0,484	-0,296	-0,174	-0,216	-0,481
	Adjusted p-value	0,347	**0,032**	0,187	0,440	0,654	0,577	0,189
hsa_miR_19a	Pearson correlation coefficient (r)	**0,830**	**-0,723**	-0,522	-0,327	-0,276	-0,252	-0,566
	Adjusted p-value	**0,006**	**0,028**	0,149	0,391	0,472	0,514	0,113
hsa_miR_19b	Pearson correlation coefficient (r)	**0,720**	**-0,911**	**-0,686**	-0,441	-0,329	-0,350	**-0,693**

	Adjusted p-value	**0,029**	**0,001**	**0,041**	0,235	0,387	0,356	**0,038**
hsa_miR_19b_1#	Pearson correlation coefficient (r)	0,094	0,015	-0,025	0,309	0,291	0,308	0,152
	Adjusted p-value	0,825	0,972	0,953	0,456	0,485	0,458	0,719
hsa_miR_200a	Pearson correlation coefficient (r)	-0,300	0,119	0,251	0,312	0,432	0,343	0,338
	Adjusted p-value	0,433	0,760	0,514	0,413	0,246	0,367	0,374
hsa_miR_200b	Pearson correlation coefficient (r)	-0,442	0,073	0,213	0,406	0,536	0,487	0,331
	Adjusted p-value	0,380	0,890	0,686	0,424	0,273	0,328	0,521
hsa_miR_202	Pearson correlation coefficient (r)	**0,748**	**-0,852**	**-0,844**	-0,547	-0,526	-0,520	**-0,800**
	Adjusted p-value	**0,033**	**0,007**	**0,008**	0,161	0,180	0,186	**0,017**
hsa_miR_202#	Pearson correlation coefficient (r)	**0,813**	**-0,689**	**-0,729**	**-0,861**	**-0,800**	**-0,814**	**-0,945**
	Adjusted p-value	**0,008**	**0,040**	**0,026**	**0,003**	**0,010**	**0,008**	**0,000**
hsa_miR_204	Pearson correlation coefficient (r)	**0,774**	**-0,824**	**-0,857**	-0,567	-0,546	-0,539	**-0,803**
	Adjusted p-value	**0,014**	**0,006**	**0,003**	0,112	0,128	0,135	**0,009**
hsa_miR_205	Pearson correlation coefficient (r)	-0,455	**0,828**	**0,956**	0,301	0,293	0,297	0,584
	Adjusted p-value	0,305	**0,021**	**0,001**	0,512	0,524	0,518	0,169
hsa_miR_20a	Pearson correlation coefficient (r)	0,378	-0,371	0,025	-0,031	0,138	0,087	-0,127
	Adjusted p-value	0,316	0,325	0,949	0,937	0,722	0,823	0,745
hsa_miR_20a#	Pearson correlation coefficient (r)	**0,831**	**-0,785**	**-0,699**	-0,664	-0,576	-0,602	**-0,819**
	Adjusted p-value	**0,006**	**0,012**	**0,036**	0,051	0,104	0,086	**0,007**
hsa_miR_20b	Pearson correlation coefficient (r)	-0,502	**0,861**	**0,910**	0,378	0,362	0,379	0,598
	Adjusted p-value	0,204	**0,006**	**0,002**	0,356	0,379	0,355	0,118
hsa_miR_21	Pearson correlation coefficient (r)	**0,718**	**-0,830**	**-0,738**	-0,626	-0,536	-0,551	**-0,805**
	Adjusted p-value	**0,029**	**0,006**	**0,023**	0,071	0,137	0,124	**0,009**
hsa_miR_21#	Pearson correlation coefficient (r)	0,448	**-0,798**	**-0,879**	-0,542	-0,555	-0,549	**-0,745**
	Adjusted p-value	0,226	**0,010**	**0,002**	0,131	0,121	0,126	**0,021**
hsa_miR_210	Pearson correlation coefficient (r)	**0,710**	**-0,710**	**-0,806**	**-0,811**	**-0,788**	**-0,793**	**-0,928**
	Adjusted p-value	**0,032**	**0,032**	**0,009**	**0,008**	**0,012**	**0,011**	**0,000**
hsa_miR_212	Pearson correlation coefficient (r)	-0,170	-0,356	-0,305	0,057	0,059	0,063	-0,088
	Adjusted p-value	0,688	0,387	0,463	0,894	0,889	0,883	0,837
hsa_miR_214	Pearson correlation coefficient (r)	0,582	**-0,842**	**-0,880**	-0,554	-0,504	-0,526	**-0,775**
	Adjusted p-value	0,100	**0,004**	**0,002**	0,122	0,167	0,145	**0,014**
hsa_miR_214#	Pearson correlation coefficient (r)	0,459	**-0,761**	**-0,810**	-0,521	-0,482	-0,497	**-0,714**
	Adjusted p-value	0,214	**0,017**	**0,008**	0,150	0,189	0,173	**0,031**

hsa_miR_215	Pearson correlation coefficient (r)	**-0,719**	**0,779**	**0,871**	**0,733**	**0,738**	**0,744**	**0,866**
	Adjusted p-value	**0,029**	**0,013**	**0,002**	**0,025**	**0,023**	**0,022**	**0,003**
hsa_miR_216a	Pearson correlation coefficient (r)	-0,645	**0,874**	**0,890**	0,699	0,673	0,678	**0,884**
	Adjusted p-value	0,084	**0,004**	**0,003**	0,054	0,067	0,065	**0,004**
hsa_miR_218	Pearson correlation coefficient (r)	0,344	-0,466	-0,121	-0,440	-0,242	-0,321	-0,434
	Adjusted p-value	0,403	0,244	0,775	0,275	0,563	0,438	0,282
hsa_miR_219_5p	Pearson correlation coefficient (r)	0,520	**-0,906**	**-0,754**	-0,100	-0,053	-0,061	-0,436
	Adjusted p-value	0,151	**0,001**	**0,019**	0,798	0,892	0,875	0,241
hsa_miR_22	Pearson correlation coefficient (r)	**0,753**	**-0,915**	**-0,866**	-0,548	-0,485	-0,496	**-0,794**
	Adjusted p-value	**0,019**	**0,001**	**0,003**	0,127	0,186	0,174	**0,011**
hsa_miR_22#	Pearson correlation coefficient (r)	**0,675**	**-0,914**	**-0,770**	-0,441	-0,329	-0,369	**-0,677**
	Adjusted p-value	**0,046**	**0,001**	**0,015**	0,235	0,387	0,329	**0,045**
hsa_miR_221	Pearson correlation coefficient (r)	0,298	-0,486	-0,620	-0,616	-0,607	-0,606	**-0,668**
	Adjusted p-value	0,435	0,185	0,075	0,077	0,083	0,084	**0,049**
hsa_miR_222	Pearson correlation coefficient (r)	-0,165	0,073	-0,095	-0,306	-0,352	-0,341	-0,173
	Adjusted p-value	0,671	0,851	0,808	0,424	0,352	0,369	0,655
hsa_miR_223	Pearson correlation coefficient (r)	**0,690**	**-0,669**	**-0,702**	-0,631	-0,597	-0,601	**-0,750**
	Adjusted p-value	**0,040**	**0,049**	**0,035**	0,068	0,089	0,087	**0,020**
hsa_miR_23a	Pearson correlation coefficient (r)	0,504	**-0,845**	**-0,851**	-0,595	-0,532	-0,558	**-0,788**
	Adjusted p-value	0,167	**0,004**	**0,004**	0,091	0,140	0,118	**0,012**
hsa_miR_23a#	Pearson correlation coefficient (r)	-0,504	0,307	0,404	**0,917**	**0,815**	**0,854**	**0,844**
	Adjusted p-value	0,203	0,459	0,321	**0,001**	**0,014**	**0,007**	**0,008**
hsa_miR_23b	Pearson correlation coefficient (r)	0,299	**-0,713**	-0,644	-0,563	-0,464	-0,511	**-0,677**
	Adjusted p-value	0,434	**0,031**	0,061	0,115	0,208	0,160	**0,045**
hsa_miR_24	Pearson correlation coefficient (r)	0,450	**-0,771**	**-0,763**	-0,647	-0,562	-0,597	**-0,788**
	Adjusted p-value	0,225	**0,015**	**0,017**	0,060	0,115	0,090	**0,012**
hsa_miR_24_1#	Pearson correlation coefficient (r)	-0,137	-0,091	-0,331	-0,242	-0,177	-0,258	-0,203
	Adjusted p-value	0,747	0,830	0,424	0,564	0,675	0,537	0,630
hsa_miR_24_2#	Pearson correlation coefficient (r)	0,123	-0,323	-0,470	-0,612	-0,639	-0,629	-0,593
	Adjusted p-value	0,771	0,435	0,240	0,107	0,088	0,095	0,121
hsa_miR_25	Pearson correlation coefficient (r)	-0,226	-0,102	0,343	0,463	0,619	0,550	0,416
	Adjusted p-value	0,558	0,795	0,367	0,209	0,076	0,125	0,265
hsa_miR_26a	Pearson correlation coefficient (r)	**0,758**	**-0,801**	**-0,855**	**-0,738**	**-0,692**	**-0,697**	**-0,922**

	Adjusted p-value	**0,018**	**0,009**	**0,003**	**0,023**	**0,039**	**0,037**	**0,000**
hsa_miR_26a_1#	Pearson correlation coefficient (r)	-0,202	0,194	0,477	0,575	0,669	0,633	0,522
	Adjusted p-value	0,631	0,645	0,232	0,136	0,070	0,092	0,184
hsa_miR_26a_2#	Pearson correlation coefficient (r)	0,401	-0,487	-0,578	**-0,751**	**-0,715**	**-0,721**	**-0,768**
	Adjusted p-value	0,285	0,184	0,103	**0,020**	**0,030**	**0,028**	**0,016**
hsa_miR_26b	Pearson correlation coefficient (r)	0,622	**-0,777**	-0,585	-0,327	-0,208	-0,242	-0,544
	Adjusted p-value	0,074	**0,014**	0,098	0,390	0,591	0,530	0,130
hsa_miR_26b#	Pearson correlation coefficient (r)	0,080	-0,667	-0,736	-0,063	-0,048	-0,047	-0,350
	Adjusted p-value	0,865	0,101	0,059	0,893	0,919	0,920	0,441
hsa_miR_27a	Pearson correlation coefficient (r)	0,552	**-0,804**	**-0,703**	-0,582	-0,483	-0,514	**-0,744**
	Adjusted p-value	0,123	**0,009**	**0,035**	0,100	0,188	0,157	**0,022**
hsa_miR_27b	Pearson correlation coefficient (r)	0,444	**-0,744**	-0,635	-0,571	-0,470	-0,505	**-0,702**
	Adjusted p-value	0,231	**0,021**	0,066	0,108	0,201	0,166	**0,035**
hsa_miR_27b#	Pearson correlation coefficient (r)	0,544	-0,207	-0,154	**-0,805**	**-0,759**	**-0,786**	-0,628
	Adjusted p-value	0,207	0,657	0,742	**0,029**	**0,048**	**0,036**	0,131
hsa_miR_28_3p	Pearson correlation coefficient (r)	0,609	**-0,891**	**-0,775**	-0,518	-0,449	-0,482	**-0,708**
	Adjusted p-value	0,082	**0,001**	**0,014**	0,153	0,225	0,188	**0,033**
hsa_miR_28_5p	Pearson correlation coefficient (r)	**0,719**	**-0,824**	**-0,724**	-0,463	-0,354	-0,395	-0,661
	Adjusted p-value	**0,029**	**0,006**	**0,027**	0,209	0,350	0,293	0,053
hsa_miR_296_3p	Pearson correlation coefficient (r)	-0,442	0,401	**0,689**	**0,809**	**0,868**	**0,834**	**0,820**
	Adjusted p-value	0,233	0,285	**0,040**	**0,008**	**0,002**	**0,005**	**0,007**
hsa_miR_296_5p	Pearson correlation coefficient (r)	-0,539	**0,683**	**0,852**	**0,754**	**0,733**	**0,733**	**0,907**
	Adjusted p-value	0,134	**0,043**	**0,004**	**0,019**	**0,025**	**0,025**	**0,001**
hsa_miR_299_3p	Pearson correlation coefficient (r)	0,440	-0,670	**-0,756**	-0,424	-0,422	-0,438	-0,565
	Adjusted p-value	0,323	0,099	**0,049**	0,343	0,345	0,326	0,187
hsa_miR_299_5p	Pearson correlation coefficient (r)	0,524	**-0,767**	**-0,808**	-0,588	-0,524	-0,547	**-0,776**
	Adjusted p-value	0,147	**0,016**	**0,008**	0,096	0,148	0,127	**0,014**
hsa_miR_29a	Pearson correlation coefficient (r)	**0,717**	**-0,904**	**-0,802**	-0,485	-0,397	-0,425	**-0,727**
	Adjusted p-value	**0,030**	**0,001**	**0,009**	0,186	0,290	0,254	**0,026**
hsa_miR_29a#	Pearson correlation coefficient (r)	0,528	**-0,834**	**-0,737**	-0,460	-0,387	-0,398	**-0,673**
	Adjusted p-value	0,144	**0,005**	**0,023**	0,213	0,304	0,288	**0,047**
hsa_miR_29b	Pearson correlation coefficient (r)	0,634	**-0,774**	**-0,733**	-0,596	-0,515	-0,536	**-0,765**
	Adjusted p-value	0,067	**0,014**	**0,025**	0,090	0,156	0,137	**0,016**

hsa_miR_29b_1#	Pearson correlation coefficient (r)	0,623	**-0,714**	**-0,709**	-0,453	-0,421	-0,460	**-0,593**
	Adjusted p-value	0,073	**0,031**	**0,032**	0,220	0,260	0,213	**0,093**
hsa_miR_29b_2#	Pearson correlation coefficient (r)	0,611	**-0,807**	**-0,845**	-0,361	-0,360	-0,360	-0,615
	Adjusted p-value	0,081	**0,009**	**0,004**	0,340	0,342	0,342	0,078
hsa_miR_29c	Pearson correlation coefficient (r)	**0,733**	**-0,887**	**-0,837**	-0,589	-0,527	-0,539	**-0,810**
	Adjusted p-value	**0,025**	**0,001**	**0,005**	0,095	0,145	0,134	**0,008**
hsa_miR_29c#	Pearson correlation coefficient (r)	**0,703**	**-0,907**	**-0,984**	-0,573	-0,564	-0,562	**-0,827**
	Adjusted p-value	**0,035**	**0,001**	**0,000**	0,107	0,114	0,115	**0,006**
hsa_miR_301a	Pearson correlation coefficient (r)	0,066	-0,401	-0,082	0,355	0,396	0,419	0,107
	Adjusted p-value	0,867	0,285	0,835	0,349	0,291	0,262	0,785
hsa_miR_30a#	Pearson correlation coefficient (r)	-0,100	0,239	0,648	0,600	0,658	0,641	0,632
	Adjusted p-value	0,799	0,535	0,059	0,088	0,054	0,063	0,068
hsa_miR_30b	Pearson correlation coefficient (r)	0,487	**-0,700**	-0,592	-0,474	-0,376	-0,400	-0,635
	Adjusted p-value	0,184	**0,036**	0,093	0,198	0,318	0,286	0,066
hsa_miR_30c	Pearson correlation coefficient (r)	0,584	**-0,878**	**-0,832**	-0,564	-0,471	-0,506	**-0,789**
	Adjusted p-value	0,099	**0,002**	**0,005**	0,114	0,201	0,165	**0,012**
hsa_miR_30d	Pearson correlation coefficient (r)	0,537	**-0,779**	**-0,769**	-0,500	-0,468	-0,452	**-0,739**
	Adjusted p-value	0,136	**0,013**	**0,015**	0,170	0,204	0,222	**0,023**
hsa_miR_30d#	Pearson correlation coefficient (r)	-0,468	0,374	0,544	0,497	0,456	0,472	0,568
	Adjusted p-value	0,204	0,322	0,130	0,173	0,217	0,200	0,111
hsa_miR_30e	Pearson correlation coefficient (r)	0,196	-0,312	-0,134	-0,484	-0,369	-0,439	-0,426
	Adjusted p-value	0,613	0,413	0,732	0,187	0,328	0,238	0,253
hsa_miR_30e#	Pearson correlation coefficient (r)	-0,342	0,159	0,518	**0,696**	**0,804**	**0,761**	0,637
	Adjusted p-value	0,368	0,683	0,154	**0,037**	**0,009**	**0,017**	0,065
hsa_miR_31	Pearson correlation coefficient (r)	**-0,786**	**0,864**	**0,882**	**0,680**	**0,674**	0,658	**0,877**
	Adjusted p-value	**0,012**	**0,003**	**0,002**	**0,044**	**0,047**	0,054	**0,002**
hsa_miR_31#	Pearson correlation coefficient (r)	-0,458	0,677	**0,816**	**0,755**	**0,711**	**0,755**	**0,835**
	Adjusted p-value	0,253	0,065	**0,013**	**0,030**	**0,048**	**0,030**	**0,010**
hsa_miR_32	Pearson correlation coefficient (r)	**0,678**	**-0,769**	-0,537	-0,431	-0,309	-0,343	-0,603
	Adjusted p-value	**0,045**	**0,015**	0,136	0,247	0,419	0,367	0,086
hsa_miR_320a	Pearson correlation coefficient (r)	**0,716**	**-0,903**	**-0,952**	-0,606	-0,576	-0,586	**-0,847**
	Adjusted p-value	**0,030**	**0,001**	**0,000**	0,084	0,105	0,097	**0,004**
hsa_miR_320b	Pearson correlation coefficient (r)	**0,741**	**-0,867**	**-0,920**	**-0,678**	-0,643	-0,651	**-0,894**

	Adjusted p-value	**0,022**	**0,002**	**0,000**	**0,045**	0,062	0,058	**0,001**
hsa_miR_323_3p	Pearson correlation coefficient (r)	0,549	**-0,727**	-0,497	-0,391	-0,227	-0,291	-0,542
	Adjusted p-value	0,126	**0,027**	0,173	0,298	0,557	0,447	0,131
hsa_miR_324_3p	Pearson correlation coefficient (r)	**0,791**	**-0,901**	**-0,876**	-0,566	-0,505	-0,526	**-0,810**
	Adjusted p-value	**0,011**	**0,001**	**0,002**	0,112	0,166	0,145	**0,008**
hsa_miR_324_5p	Pearson correlation coefficient (r)	**0,755**	**-0,847**	**-0,849**	-0,605	-0,546	-0,564	**-0,830**
	Adjusted p-value	**0,019**	**0,004**	**0,004**	0,085	0,129	0,113	**0,006**
hsa_miR_326	Pearson correlation coefficient (r)	0,162	-0,445	-0,501	-0,228	-0,202	-0,229	-0,363
	Adjusted p-value	0,676	0,231	0,169	0,556	0,602	0,553	0,337
hsa_miR_328	Pearson correlation coefficient (r)	**0,726**	**-0,832**	**-0,848**	-0,648	-0,626	-0,631	**-0,848**
	Adjusted p-value	**0,027**	**0,005**	**0,004**	0,059	0,071	0,068	**0,004**
hsa_miR_329	Pearson correlation coefficient (r)	0,543	**-0,739**	**-0,860**	**-0,726**	-0,662	**-0,700**	**-0,867**
	Adjusted p-value	0,130	**0,023**	**0,003**	**0,027**	0,052	**0,036**	**0,002**
hsa_miR_330_5p	Pearson correlation coefficient (r)	0,291	-0,233	-0,630	**-0,747**	**-0,756**	**-0,761**	**-0,745**
	Adjusted p-value	0,485	0,579	0,094	**0,033**	**0,030**	**0,028**	**0,034**
hsa_miR_331_3p	Pearson correlation coefficient (r)	0,661	**-0,827**	**-0,794**	-0,549	-0,496	-0,508	**-0,766**
	Adjusted p-value	0,053	**0,006**	**0,011**	0,126	0,175	0,163	**0,016**
hsa_miR_335	Pearson correlation coefficient (r)	0,537	-0,433	-0,116	-0,243	-0,060	-0,126	-0,302
	Adjusted p-value	0,136	0,245	0,767	0,528	0,879	0,746	0,429
hsa_miR_337_3p	Pearson correlation coefficient (r)	**0,904**	**-0,888**	**-0,863**	-0,495	-0,467	-0,472	**-0,737**
	Adjusted p-value	**0,001**	**0,001**	**0,003**	0,176	0,205	0,199	**0,023**
hsa_miR_337_5p	Pearson correlation coefficient (r)	0,596	**-0,696**	**-0,793**	-0,576	-0,550	-0,558	**-0,747**
	Adjusted p-value	0,090	**0,037**	**0,011**	0,105	0,125	0,118	**0,021**
hsa_miR_338_3p	Pearson correlation coefficient (r)	0,124	-0,602	-0,338	-0,052	0,082	0,026	-0,234
	Adjusted p-value	0,751	0,086	0,373	0,895	0,834	0,947	0,544
hsa_miR_339_3p	Pearson correlation coefficient (r)	0,662	**-0,893**	**-0,873**	-0,610	-0,541	-0,556	**-0,846**
	Adjusted p-value	0,052	**0,001**	**0,002**	0,081	0,133	0,120	**0,004**
hsa_miR_339_5p	Pearson correlation coefficient (r)	0,617	**-0,794**	**-0,796**	-0,653	-0,597	-0,613	**-0,828**
	Adjusted p-value	0,077	**0,011**	**0,010**	0,056	0,089	0,079	**0,006**
hsa_miR_33a	Pearson correlation coefficient (r)	**0,679**	**-0,847**	**-0,726**	-0,469	-0,394	-0,413	**-0,690**
	Adjusted p-value	**0,044**	**0,004**	**0,027**	0,203	0,294	0,270	**0,039**
hsa_miR_33a#	Pearson correlation coefficient (r)	0,428	-0,575	-0,592	-0,617	-0,602	-0,604	**-0,708**
	Adjusted p-value	0,250	0,106	0,093	0,077	0,086	0,085	**0,033**

hsa_miR_33b	Pearson correlation coefficient (r)	**0,767**	**-0,977**	**-0,916**	-0,515	-0,464	-0,481	**-0,781**
	Adjusted p-value	**0,026**	**0,000**	**0,001**	0,191	0,246	0,228	**0,022**
hsa_miR_340	Pearson correlation coefficient (r)	-0,323	0,516	0,374	0,089	0,003	0,062	0,255
	Adjusted p-value	0,397	0,155	0,321	0,819	0,994	0,875	0,508
hsa_miR_340#	Pearson correlation coefficient (r)	-0,527	0,584	0,467	**0,762**	**0,740**	**0,749**	**0,767**
	Adjusted p-value	0,179	0,128	0,244	**0,028**	**0,036**	**0,032**	**0,026**
hsa_miR_342_3p	Pearson correlation coefficient (r)	0,202	-0,181	-0,383	**-0,730**	-0,667	**-0,703**	-0,643
	Adjusted p-value	0,602	0,641	0,309	**0,026**	0,050	**0,035**	0,062
hsa_miR_34a	Pearson correlation coefficient (r)	**0,682**	**-0,917**	**-0,887**	-0,480	-0,425	-0,442	**-0,746**
	Adjusted p-value	**0,043**	**0,000**	**0,001**	0,191	0,255	0,234	**0,021**
hsa_miR_34a#	Pearson correlation coefficient (r)	0,637	**-0,794**	**-0,742**	-0,501	-0,444	-0,445	**-0,723**
	Adjusted p-value	0,065	**0,011**	**0,022**	0,169	0,231	0,230	**0,028**
hsa_miR_34b	Pearson correlation coefficient (r)	-0,526	0,520	**0,784**	**0,907**	**0,931**	**0,921**	**0,933**
	Adjusted p-value	0,146	0,151	**0,012**	**0,001**	**0,000**	**0,000**	**0,000**
hsa_miR_34b#	Pearson correlation coefficient (r)	-0,507	0,503	**0,795**	**0,867**	**0,899**	**0,882**	**0,913**
	Adjusted p-value	0,164	0,167	**0,010**	**0,002**	**0,001**	**0,002**	**0,001**
hsa_miR_34c_5p	Pearson correlation coefficient (r)	-0,513	0,522	**0,816**	**0,842**	**0,883**	**0,862**	**0,902**
	Adjusted p-value	0,158	0,149	**0,007**	**0,004**	**0,002**	**0,003**	**0,001**
hsa_miR_361_3p	Pearson correlation coefficient (r)	0,585	**-0,725**	**-0,809**	-0,525	-0,505	-0,515	**-0,725**
	Adjusted p-value	0,098	**0,027**	**0,008**	0,147	0,166	0,156	**0,027**
hsa_miR_362_3p	Pearson correlation coefficient (r)	0,343	-0,577	**-0,688**	-0,343	-0,298	-0,331	-0,491
	Adjusted p-value	0,366	0,104	**0,040**	0,367	0,436	0,385	0,180
hsa_miR_362_5p	Pearson correlation coefficient (r)	**0,798**	**-0,741**	**-0,850**	-0,510	-0,516	-0,508	**-0,715**
	Adjusted p-value	**0,010**	**0,022**	**0,004**	0,161	0,155	0,163	**0,030**
hsa_miR_363	Pearson correlation coefficient (r)	-0,336	0,253	0,396	0,014	0,081	0,052	0,192
	Adjusted p-value	0,461	0,585	0,380	0,976	0,863	0,912	0,680
hsa_miR_365	Pearson correlation coefficient (r)	**0,762**	**-0,884**	**-0,761**	-0,548	-0,468	-0,486	**-0,749**
	Adjusted p-value	**0,017**	**0,002**	**0,017**	0,127	0,204	0,185	**0,020**
hsa_miR_369_5p	Pearson correlation coefficient (r)	0,139	-0,478	-0,441	-0,467	-0,348	-0,402	-0,527
	Adjusted p-value	0,722	0,193	0,235	0,205	0,359	0,283	0,145
hsa_miR_370	Pearson correlation coefficient (r)	0,600	**-0,819**	**-0,706**	-0,330	-0,235	-0,280	-0,569
	Adjusted p-value	0,088	**0,007**	**0,033**	0,385	0,543	0,466	0,110
hsa_miR_371_3p	Pearson correlation coefficient (r)	-0,543	0,423	0,530	0,496	0,597	0,521	0,581

	Adjusted p-value	0,131	0,256	0,142	0,175	0,090	0,150	0,101
hsa_miR_372	Pearson correlation coefficient (r)	-0,097	0,323	0,619	0,545	0,506	0,507	0,671
	Adjusted p-value	0,836	0,479	0,138	0,206	0,247	0,245	0,099
hsa_miR_373	Pearson correlation coefficient (r)	**-0,759**	**0,829**	**0,784**	**0,717**	0,629	0,640	**0,879**
	Adjusted p-value	**0,029**	**0,011**	**0,021**	**0,045**	0,095	0,087	**0,004**
hsa_miR_373#	Pearson correlation coefficient (r)	-0,563	0,278	0,481	0,719	**0,820**	0,747	0,719
	Adjusted p-value	0,189	0,546	0,274	0,068	**0,024**	0,053	0,068
hsa_miR_374a	Pearson correlation coefficient (r)	**0,705**	**-0,925**	**-0,898**	-0,568	-0,522	-0,525	**-0,819**
	Adjusted p-value	**0,034**	**0,000**	**0,001**	0,110	0,150	0,147	**0,007**
hsa_miR_374b	Pearson correlation coefficient (r)	**0,793**	**-0,701**	**-0,679**	**-0,754**	-0,664	-0,690	**-0,858**
	Adjusted p-value	**0,011**	**0,035**	**0,044**	**0,019**	0,051	0,040	**0,003**
hsa_miR_374b#	Pearson correlation coefficient (r)	**0,754**	**-0,947**	**-0,926**	-0,528	-0,493	-0,497	**-0,794**
	Adjusted p-value	**0,019**	**0,000**	**0,000**	0,144	0,178	0,173	**0,011**
hsa_miR_375	Pearson correlation coefficient (r)	-0,507	0,536	**0,829**	**0,774**	**0,815**	**0,787**	**0,870**
	Adjusted p-value	0,164	0,137	**0,006**	**0,014**	**0,007**	**0,012**	**0,002**
hsa_miR_376a	Pearson correlation coefficient (r)	**0,761**	**-0,836**	**-0,738**	-0,426	-0,354	-0,371	-0,661
	Adjusted p-value	**0,017**	**0,005**	**0,023**	0,253	0,350	0,325	0,053
hsa_miR_376b	Pearson correlation coefficient (r)	0,655	**-0,870**	**-0,851**	-0,456	-0,420	-0,421	**-0,722**
	Adjusted p-value	0,055	**0,002**	**0,004**	0,218	0,261	0,260	**0,028**
hsa_miR_376c	Pearson correlation coefficient (r)	0,599	**-0,796**	**-0,768**	-0,427	-0,374	-0,388	-0,656
	Adjusted p-value	0,089	**0,010**	**0,016**	0,252	0,321	0,302	0,055
hsa_miR_377	Pearson correlation coefficient (r)	0,601	**-0,797**	**-0,757**	-0,432	-0,358	-0,385	-0,655
	Adjusted p-value	0,087	**0,010**	**0,018**	0,245	0,344	0,306	0,056
hsa_miR_378	Pearson correlation coefficient (r)	-0,258	-0,043	-0,099	0,354	0,271	0,316	0,223
	Adjusted p-value	0,502	0,912	0,800	0,350	0,481	0,407	0,564
hsa_miR_379	Pearson correlation coefficient (r)	**0,742**	**-0,778**	**-0,709**	-0,407	-0,357	-0,366	-0,633
	Adjusted p-value	**0,022**	**0,014**	**0,033**	0,277	0,345	0,332	0,067
hsa_miR_381	Pearson correlation coefficient (r)	**0,735**	**-0,876**	**-0,834**	-0,529	-0,472	-0,484	**-0,765**
	Adjusted p-value	**0,024**	**0,002**	**0,005**	0,143	0,200	0,187	**0,016**
hsa_miR_382	Pearson correlation coefficient (r)	0,638	**-0,764**	**-0,761**	-0,431	-0,381	-0,403	-0,644
	Adjusted p-value	0,064	**0,017**	**0,017**	0,247	0,311	0,282	0,061
hsa_miR_383	Pearson correlation coefficient (r)	-0,169	-0,047	-0,430	-0,539	-0,610	-0,632	-0,418
	Adjusted p-value	0,717	0,920	0,335	0,212	0,146	0,128	0,350

hsa_miR_409_3p	Pearson correlation coefficient (r)	0,522	**-0,804**	**-0,736**	-0,516	-0,401	-0,449	**-0,708**
	Adjusted p-value	0,149	**0,009**	**0,024**	0,155	0,285	0,225	**0,033**
hsa_miR_409_5p	Pearson correlation coefficient (r)	-0,226	0,056	-0,249	-0,036	-0,087	-0,065	-0,109
	Adjusted p-value	0,558	0,887	0,518	0,927	0,824	0,868	0,781
hsa_miR_410	Pearson correlation coefficient (r)	0,262	-0,537	-0,495	-0,362	-0,282	-0,325	-0,481
	Adjusted p-value	0,496	0,136	0,175	0,338	0,462	0,394	0,190
hsa_miR_411	Pearson correlation coefficient (r)	0,526	**-0,778**	**-0,689**	-0,354	-0,263	-0,301	-0,573
	Adjusted p-value	0,145	**0,013**	**0,040**	0,350	0,494	0,432	0,107
hsa_miR_421	Pearson correlation coefficient (r)	**0,736**	**-0,797**	**-0,703**	-0,438	-0,390	-0,394	-0,655
	Adjusted p-value	**0,024**	**0,010**	**0,035**	0,238	0,300	0,294	0,056
hsa_miR_423_3p	Pearson correlation coefficient (r)	**0,766**	**-0,903**	**-0,857**	-0,522	-0,457	-0,488	**-0,759**
	Adjusted p-value	**0,016**	**0,001**	**0,003**	0,150	0,217	0,182	**0,018**
hsa_miR_423_5p	Pearson correlation coefficient (r)	**0,688**	**-0,881**	**-0,929**	-0,545	-0,536	-0,531	**-0,804**
	Adjusted p-value	**0,041**	**0,002**	**0,000**	0,129	0,137	0,141	**0,009**
hsa_miR_423_5p_dup	Pearson correlation coefficient (r)	**0,742**	**-0,851**	**-0,861**	**-0,677**	-0,663	-0,654	**-0,881**
	Adjusted p-value	**0,022**	**0,004**	**0,003**	**0,045**	0,051	0,056	**0,002**
hsa_miR_424	Pearson correlation coefficient (r)	**0,799**	**-0,928**	**-0,823**	-0,328	-0,290	-0,292	-0,641
	Adjusted p-value	**0,017**	**0,001**	**0,012**	0,428	0,485	0,483	0,087
hsa_miR_424#	Pearson correlation coefficient (r)	**0,810**	**-0,934**	**-0,837**	-0,431	-0,397	-0,394	**-0,710**
	Adjusted p-value	**0,008**	**0,000**	**0,005**	0,247	0,290	0,295	**0,032**
hsa_miR_425	Pearson correlation coefficient (r)	0,496	-0,429	-0,166	-0,230	-0,130	-0,134	-0,308
	Adjusted p-value	0,174	0,250	0,670	0,552	0,740	0,732	0,420
hsa_miR_425#	Pearson correlation coefficient (r)	**0,732**	-0,424	-0,170	-0,032	0,050	0,059	-0,207
	Adjusted p-value	**0,025**	0,255	0,661	0,935	0,898	0,881	0,593
hsa_miR_431	Pearson correlation coefficient (r)	0,242	-0,068	-0,031	0,217	0,184	0,188	0,102
	Adjusted p-value	0,564	0,873	0,941	0,607	0,663	0,656	0,809
hsa_miR_431#	Pearson correlation coefficient (r)	**0,850**	**-0,886**	**-0,816**	-0,460	-0,407	-0,436	-0,684
	Adjusted p-value	**0,007**	**0,003**	**0,013**	0,251	0,317	0,281	0,061
hsa_miR_432	Pearson correlation coefficient (r)	0,410	-0,654	-0,459	-0,209	-0,091	-0,140	-0,393
	Adjusted p-value	0,273	0,056	0,214	0,590	0,815	0,720	0,296
hsa_miR_433	Pearson correlation coefficient (r)	0,273	-0,610	-0,679	-0,099	-0,102	-0,132	-0,278
	Adjusted p-value	0,553	0,145	0,093	0,834	0,829	0,778	0,547
hsa_miR_449a	Pearson correlation coefficient (r)	-0,510	0,477	**0,765**	**0,873**	**0,915**	**0,890**	**0,907**

	Adjusted p-value	0,161	0,194	**0,016**	**0,002**	**0,001**	**0,001**	**0,001**
hsa_miR_449b	Pearson correlation coefficient (r)	-0,496	0,557	**0,828**	**0,826**	**0,845**	**0,836**	**0,894**
	Adjusted p-value	0,175	0,119	**0,006**	**0,006**	**0,004**	**0,005**	**0,001**
hsa_miR_449b#	Pearson correlation coefficient (r)	-0,753	**0,969**	**0,926**	**0,828**	**0,779**	**0,807**	**0,939**
	Adjusted p-value	0,051	**0,000**	**0,003**	**0,021**	**0,039**	**0,028**	**0,002**
hsa_miR_450a	Pearson correlation coefficient (r)	**0,833**	**-0,929**	**-0,787**	-0,451	-0,357	-0,397	**-0,690**
	Adjusted p-value	**0,005**	**0,000**	**0,012**	0,224	0,346	0,290	**0,040**
hsa_miR_450b_3p	Pearson correlation coefficient (r)	0,480	-0,351	0,141	-0,081	0,083	0,041	-0,117
	Adjusted p-value	0,228	0,393	0,740	0,849	0,845	0,924	0,783
hsa_miR_450b_5p	Pearson correlation coefficient (r)	**0,669**	**-0,795**	**-0,682**	-0,170	-0,127	-0,127	-0,456
	Adjusted p-value	**0,049**	**0,010**	**0,043**	0,662	0,745	0,744	0,217
hsa_miR_451	Pearson correlation coefficient (r)	-0,453	0,396	0,368	0,085	0,102	0,092	0,269
	Adjusted p-value	0,221	0,291	0,330	0,827	0,794	0,814	0,485
hsa_miR_452	Pearson correlation coefficient (r)	-0,584	0,343	0,110	0,081	0,118	0,041	0,211
	Adjusted p-value	0,169	0,451	0,814	0,863	0,802	0,930	0,650
hsa_miR_454	Pearson correlation coefficient (r)	0,322	-0,225	-0,225	-0,479	-0,450	-0,435	-0,443
	Adjusted p-value	0,398	0,561	0,560	0,192	0,224	0,241	0,232
hsa_miR_455_3p	Pearson correlation coefficient (r)	**0,768**	**-0,920**	**-0,941**	-0,566	-0,550	-0,546	**-0,824**
	Adjusted p-value	**0,016**	**0,000**	**0,000**	0,112	0,125	0,128	**0,006**
hsa_miR_455_5p	Pearson correlation coefficient (r)	**0,681**	**-0,926**	**-0,914**	-0,564	-0,519	-0,526	**-0,817**
	Adjusted p-value	**0,043**	**0,000**	**0,001**	0,114	0,152	0,146	**0,007**
hsa_miR_483_3p	Pearson correlation coefficient (r)	0,107	-0,183	-0,461	-0,275	-0,280	-0,300	-0,350
	Adjusted p-value	0,784	0,637	0,211	0,473	0,465	0,432	0,356
hsa_miR_484	Pearson correlation coefficient (r)	-0,124	-0,293	-0,219	-0,164	-0,039	-0,115	-0,198
	Adjusted p-value	0,751	0,443	0,571	0,673	0,921	0,768	0,610
hsa_miR_485_3p	Pearson correlation coefficient (r)	**0,732**	-0,656	**-0,736**	-0,654	-0,627	-0,646	**-0,765**
	Adjusted p-value	**0,025**	0,055	**0,024**	0,056	0,071	0,060	**0,016**
hsa_miR_486_5p	Pearson correlation coefficient (r)	-0,564	0,469	0,476	0,286	0,289	0,294	0,459
	Adjusted p-value	0,146	0,241	0,233	0,492	0,488	0,480	0,252
hsa_miR_487b	Pearson correlation coefficient (r)	0,411	**-0,669**	-0,497	-0,129	0,006	-0,061	-0,337
	Adjusted p-value	0,271	**0,049**	0,174	0,741	0,987	0,875	0,376
hsa_miR_488	Pearson correlation coefficient (r)	**-0,736**	**0,714**	**0,797**	**0,819**	**0,784**	**0,776**	**0,958**
	Adjusted p-value	**0,024**	**0,031**	**0,010**	**0,007**	**0,012**	**0,014**	**0,000**

hsa_miR_489	Pearson correlation coefficient (r)	**0,857**	**-0,910**	**-0,788**	-0,584	-0,501	-0,532	**-0,801**
	Adjusted p-value	**0,014**	**0,004**	**0,035**	0,169	0,252	0,219	**0,030**
hsa_miR_491_5p	Pearson correlation coefficient (r)	0,616	**-0,793**	**-0,782**	-0,617	-0,602	-0,597	**-0,795**
	Adjusted p-value	0,104	**0,019**	**0,022**	0,104	0,114	0,118	**0,018**
hsa_miR_493#	Pearson correlation coefficient (r)	0,517	**-0,685**	-0,552	-0,322	-0,213	-0,258	-0,508
	Adjusted p-value	0,154	**0,042**	0,123	0,398	0,582	0,502	0,163
hsa_miR_494	Pearson correlation coefficient (r)	0,518	**-0,810**	**-0,748**	-0,499	-0,382	-0,445	**-0,680**
	Adjusted p-value	0,153	**0,008**	**0,020**	0,171	0,310	0,230	**0,044**
hsa_miR_495	Pearson correlation coefficient (r)	0,547	-0,651	-0,616	-0,518	-0,415	-0,465	-0,648
	Adjusted p-value	0,128	0,058	0,078	0,153	0,267	0,207	0,059
hsa_miR_497	Pearson correlation coefficient (r)	0,603	**-0,927**	**-0,829**	-0,446	-0,371	-0,393	**-0,707**
	Adjusted p-value	0,086	**0,000**	**0,006**	0,229	0,326	0,295	**0,033**
hsa_miR_498	Pearson correlation coefficient (r)	-0,534	**0,758**	**0,687**	0,399	0,356	0,360	0,578
	Adjusted p-value	0,139	**0,018**	**0,041**	0,288	0,347	0,341	0,103
hsa_miR_499_5p	Pearson correlation coefficient (r)	-0,534	0,297	0,400	**0,807**	**0,728**	**0,766**	**0,769**
	Adjusted p-value	0,173	0,476	0,326	**0,015**	**0,041**	**0,027**	**0,026**
hsa_miR_500	Pearson correlation coefficient (r)	**0,759**	**-0,829**	**-0,869**	-0,376	-0,348	-0,348	**-0,672**
	Adjusted p-value	**0,018**	**0,006**	**0,002**	0,319	0,358	0,358	**0,047**
hsa_miR_501_3p	Pearson correlation coefficient (r)	**0,742**	-0,536	**-0,697**	-0,470	-0,507	-0,485	-0,626
	Adjusted p-value	**0,022**	0,137	**0,037**	0,202	0,164	0,186	0,071
hsa_miR_501_5p	Pearson correlation coefficient (r)	0,488	-0,482	-0,656	-0,439	-0,418	-0,424	-0,591
	Adjusted p-value	0,183	0,189	0,055	0,237	0,263	0,255	0,094
hsa_miR_502_3p	Pearson correlation coefficient (r)	**0,835**	**-0,767**	**-0,864**	**-0,720**	**-0,726**	**-0,703**	**-0,907**
	Adjusted p-value	**0,005**	**0,016**	**0,003**	**0,029**	**0,027**	**0,035**	**0,001**
hsa_miR_503	Pearson correlation coefficient (r)	**0,899**	**-0,909**	**-0,823**	-0,515	-0,480	-0,470	**-0,782**
	Adjusted p-value	**0,001**	**0,001**	**0,006**	0,155	0,191	0,202	**0,013**
hsa_miR_504	Pearson correlation coefficient (r)	0,640	-0,374	-0,594	**-0,965**	**-0,950**	**-0,955**	**-0,921**
	Adjusted p-value	0,122	0,408	0,160	**0,000**	**0,001**	**0,001**	**0,003**
hsa_miR_505	Pearson correlation coefficient (r)	0,346	-0,574	**-0,720**	-0,567	-0,542	-0,570	**-0,679**
	Adjusted p-value	0,361	0,106	**0,029**	0,112	0,132	0,109	**0,044**
hsa_miR_506	Pearson correlation coefficient (r)	**0,898**	**-0,857**	**-0,816**	-0,534	-0,480	-0,487	**-0,773**
	Adjusted p-value	**0,001**	**0,003**	**0,007**	0,139	0,191	0,183	**0,015**
hsa_miR_508_3p	Pearson correlation coefficient (r)	**0,869**	**-0,895**	**-0,904**	-0,468	-0,429	-0,438	**-0,764**

	Adjusted p-value	**0,005**	**0,003**	**0,002**	0,243	0,289	0,278	**0,027**
hsa_miR_508_5p	Pearson correlation coefficient (r)	**0,818**	**-0,782**	**-0,807**	-0,435	-0,428	-0,413	**-0,692**
	Adjusted p-value	**0,007**	**0,013**	**0,009**	0,242	0,250	0,270	**0,039**
hsa_miR_509_3_5p	Pearson correlation coefficient (r)	0,645	**-0,915**	**-0,744**	-0,424	-0,311	-0,348	**-0,685**
	Adjusted p-value	0,061	**0,001**	**0,022**	0,255	0,415	0,359	**0,042**
hsa_miR_509_3p	Pearson correlation coefficient (r)	**0,839**	**-0,848**	**-0,856**	-0,479	-0,463	-0,457	**-0,737**
	Adjusted p-value	**0,005**	**0,004**	**0,003**	0,192	0,210	0,217	**0,023**
hsa_miR_510	Pearson correlation coefficient (r)	**0,813**	**-0,789**	**-0,755**	-0,527	-0,514	-0,496	**-0,747**
	Adjusted p-value	**0,008**	**0,012**	**0,019**	0,145	0,157	0,174	**0,021**
hsa_miR_511	Pearson correlation coefficient (r)	-0,518	0,279	0,490	**0,995**	**0,969**	**0,984**	**0,900**
	Adjusted p-value	0,188	0,503	0,217	**0,000**	**0,000**	**0,000**	**0,002**
hsa_miR_512_5p	Pearson correlation coefficient (r)	**-0,785**	**0,882**	**0,907**	**0,754**	**0,713**	**0,719**	**0,941**
	Adjusted p-value	**0,012**	**0,002**	**0,001**	**0,019**	**0,031**	**0,029**	**0,000**
hsa_miR_513a_3p	Pearson correlation coefficient (r)	**0,962**	**-0,862**	**-0,776**	-0,487	-0,464	-0,442	**-0,746**
	Adjusted p-value	**0,000**	**0,003**	**0,014**	0,184	0,209	0,234	**0,021**
hsa_miR_513a_5p	Pearson correlation coefficient (r)	**0,888**	**-0,804**	**-0,755**	-0,437	-0,413	-0,403	**-0,683**
	Adjusted p-value	**0,001**	**0,009**	**0,019**	0,240	0,269	0,283	**0,042**
hsa_miR_513c	Pearson correlation coefficient (r)	**0,934**	**-0,793**	**-0,720**	-0,440	-0,401	-0,396	**-0,675**
	Adjusted p-value	**0,000**	**0,011**	**0,029**	0,236	0,285	0,292	**0,046**
hsa_miR_514	Pearson correlation coefficient (r)	**0,920**	-0,514	-0,521	-0,458	-0,465	-0,443	-0,585
	Adjusted p-value	**0,000**	0,157	0,151	0,215	0,208	0,233	0,098
hsa_miR_515_3p	Pearson correlation coefficient (r)	**-0,840**	**0,937**	**0,797**	0,530	0,461	0,474	**0,781**
	Adjusted p-value	**0,005**	**0,000**	**0,010**	0,142	0,212	0,198	**0,013**
hsa_miR_515_5p	Pearson correlation coefficient (r)	**-0,678**	**0,911**	**0,926**	0,542	0,521	0,533	**0,764**
	Adjusted p-value	**0,045**	**0,001**	**0,000**	0,132	0,151	0,140	**0,017**
hsa_miR_516a_3p	Pearson correlation coefficient (r)	**-0,806**	**0,805**	**0,792**	0,592	0,620	0,571	**0,776**
	Adjusted p-value	**0,016**	**0,016**	**0,019**	0,122	0,101	0,139	**0,024**
hsa_miR_516a_5p	Pearson correlation coefficient (r)	-0,644	**0,731**	**0,685**	**0,750**	**0,678**	**0,695**	**0,838**
	Adjusted p-value	0,061	**0,025**	**0,042**	**0,020**	**0,045**	**0,038**	**0,005**
hsa_miR_516b	Pearson correlation coefficient (r)	-0,381	0,370	0,470	**0,996**	**0,999**	**0,999**	**0,889**
	Adjusted p-value	0,351	0,367	0,240	**0,000**	**0,000**	**0,000**	**0,003**
hsa_miR_517a	Pearson correlation coefficient (r)	**-0,760**	**0,848**	**0,956**	**0,684**	**0,691**	**0,672**	**0,904**
	Adjusted p-value	**0,017**	**0,004**	**0,000**	**0,042**	**0,039**	**0,047**	**0,001**

hsa_miR_517b	Pearson correlation coefficient (r)	**-0,677**	**0,819**	**0,932**	**0,776**	**0,744**	**0,757**	**0,936**
	Adjusted p-value	**0,045**	**0,007**	**0,000**	**0,014**	**0,021**	**0,018**	**0,000**
hsa_miR_517c	Pearson correlation coefficient (r)	**-0,714**	**0,695**	**0,793**	**0,853**	**0,860**	**0,854**	**0,933**
	Adjusted p-value	**0,031**	**0,038**	**0,011**	**0,003**	**0,003**	**0,003**	**0,000**
hsa_miR_518a_3p	Pearson correlation coefficient (r)	-0,546	0,629	**0,897**	**0,731**	**0,781**	**0,756**	**0,857**
	Adjusted p-value	0,128	0,069	**0,001**	**0,025**	**0,013**	**0,019**	**0,003**
hsa_miR_518a_5p	Pearson correlation coefficient (r)	-0,587	**0,854**	**0,890**	0,356	0,353	0,338	0,640
	Adjusted p-value	0,126	**0,007**	**0,003**	0,387	0,391	0,413	0,088
hsa_miR_518b	Pearson correlation coefficient (r)	**-0,755**	**0,974**	**0,942**	0,423	0,398	0,398	**0,752**
	Adjusted p-value	**0,030**	**0,000**	**0,000**	0,297	0,329	0,329	**0,031**
hsa_miR_518c	Pearson correlation coefficient (r)	**-0,769**	**0,948**	**0,944**	0,560	0,518	0,530	**0,824**
	Adjusted p-value	**0,015**	**0,000**	**0,000**	0,117	0,153	0,142	**0,006**
hsa_miR_518c#	Pearson correlation coefficient (r)	**-0,722**	**0,835**	**0,975**	0,591	0,615	0,606	**0,841**
	Adjusted p-value	**0,043**	**0,010**	**0,000**	0,123	0,104	0,111	**0,009**
hsa_miR_518d_5p	Pearson correlation coefficient (r)	-0,534	**0,762**	**0,784**	0,662	0,590	0,655	0,750
	Adjusted p-value	0,217	**0,047**	**0,037**	0,105	0,163	0,111	0,052
hsa_miR_518e	Pearson correlation coefficient (r)	-0,578	**0,728**	**0,886**	**0,817**	**0,781**	**0,805**	**0,924**
	Adjusted p-value	0,103	**0,026**	**0,001**	**0,007**	**0,013**	**0,009**	**0,000**
hsa_miR_518e#	Pearson correlation coefficient (r)	**-0,785**	**0,932**	**0,951**	**0,714**	**0,714**	**0,719**	**0,903**
	Adjusted p-value	**0,021**	**0,001**	**0,000**	**0,047**	**0,047**	**0,044**	**0,002**
hsa_miR_518f	Pearson correlation coefficient (r)	**-0,714**	**0,824**	**0,944**	**0,765**	**0,761**	**0,759**	**0,932**
	Adjusted p-value	**0,031**	**0,006**	**0,000**	**0,016**	**0,017**	**0,018**	**0,000**
hsa_miR_518f#	Pearson correlation coefficient (r)	-0,586	**0,868**	**0,966**	0,524	0,511	0,528	**0,749**
	Adjusted p-value	0,098	**0,002**	**0,000**	0,147	0,159	0,144	**0,020**
hsa_miR_519a	Pearson correlation coefficient (r)	-0,627	**0,755**	**0,910**	**0,800**	**0,773**	**0,784**	**0,937**
	Adjusted p-value	0,070	**0,019**	**0,001**	**0,010**	**0,015**	**0,012**	**0,000**
hsa_miR_519b_3p	Pearson correlation coefficient (r)	**-0,686**	**0,810**	**0,978**	0,609	0,649	0,626	**0,832**
	Adjusted p-value	**0,041**	**0,008**	**0,000**	0,082	0,058	0,071	**0,005**
hsa_miR_519c_3p	Pearson correlation coefficient (r)	**-0,674**	**0,679**	**0,881**	**0,845**	**0,875**	**0,857**	**0,955**
	Adjusted p-value	**0,046**	**0,044**	**0,002**	**0,004**	**0,002**	**0,003**	**0,000**
hsa_miR_519d	Pearson correlation coefficient (r)	-0,640	**0,861**	**0,895**	0,193	0,219	0,199	0,534
	Adjusted p-value	0,064	**0,003**	**0,001**	0,618	0,572	0,607	0,138
hsa_miR_519e	Pearson correlation coefficient (r)	**-0,736**	**0,777**	**0,917**	0,641	0,666	0,630	**0,868**

	Adjusted p-value	**0,024**	**0,014**	**0,001**	0,063	0,050	0,069	**0,002**
hsa_miR_519e#	Pearson correlation coefficient (r)	-0,665	**0,878**	**0,939**	0,598	0,543	0,569	**0,829**
	Adjusted p-value	0,051	**0,002**	**0,000**	0,089	0,131	0,110	**0,006**
hsa_miR_520a_3p	Pearson correlation coefficient (r)	-0,569	0,556	**0,772**	**0,733**	**0,779**	**0,736**	**0,837**
	Adjusted p-value	0,109	0,120	**0,015**	**0,025**	**0,013**	**0,024**	**0,005**
hsa_miR_520a_5p	Pearson correlation coefficient (r)	-0,612	**0,742**	**0,939**	**0,757**	**0,771**	**0,767**	**0,899**
	Adjusted p-value	0,080	**0,022**	**0,000**	**0,018**	**0,015**	**0,016**	**0,001**
hsa_miR_520b	Pearson correlation coefficient (r)	-0,664	**0,894**	**0,992**	0,494	0,491	0,494	**0,763**
	Adjusted p-value	0,051	**0,001**	**0,000**	0,176	0,180	0,177	**0,017**
hsa_miR_520c_3p	Pearson correlation coefficient (r)	**-0,694**	**0,879**	**0,992**	0,562	0,576	0,566	**0,810**
	Adjusted p-value	**0,038**	**0,002**	**0,000**	0,115	0,105	0,112	**0,008**
hsa_miR_520d_5p	Pearson correlation coefficient (r)	-0,687	**0,948**	**0,933**	**0,824**	**0,828**	**0,832**	**0,933**
	Adjusted p-value	0,132	**0,004**	**0,007**	**0,044**	**0,042**	**0,040**	**0,007**
hsa_miR_520e	Pearson correlation coefficient (r)	-0,583	**0,741**	**0,924**	0,467	0,540	0,511	**0,684**
	Adjusted p-value	0,100	**0,022**	**0,000**	0,205	0,133	0,160	**0,042**
hsa_miR_520f	Pearson correlation coefficient (r)	-0,626	**0,813**	**0,995**	0,589	0,615	0,606	**0,812**
	Adjusted p-value	0,071	**0,008**	**0,000**	0,095	0,078	0,084	**0,008**
hsa_miR_520g	Pearson correlation coefficient (r)	-0,662	**0,860**	**0,996**	0,569	0,569	0,565	**0,818**
	Adjusted p-value	0,052	**0,003**	**0,000**	0,110	0,109	0,113	**0,007**
hsa_miR_520h	Pearson correlation coefficient (r)	-0,769	**0,979**	**0,933**	0,782	0,772	0,760	**0,946**
	Adjusted p-value	0,074	**0,001**	**0,007**	0,066	0,072	0,079	**0,004**
hsa_miR_521	Pearson correlation coefficient (r)	-0,526	0,340	0,544	**0,947**	**0,950**	**0,933**	**0,909**
	Adjusted p-value	0,146	0,371	0,130	**0,000**	**0,000**	**0,000**	**0,001**
hsa_miR_522	Pearson correlation coefficient (r)	**-0,734**	**0,821**	**0,710**	**0,716**	0,642	0,664	**0,835**
	Adjusted p-value	**0,024**	**0,007**	**0,032**	**0,030**	0,062	0,051	**0,005**
hsa_miR_523	Pearson correlation coefficient (r)	**-0,751**	**0,884**	**0,677**	0,544	0,439	0,477	**0,737**
	Adjusted p-value	**0,020**	**0,002**	**0,045**	0,130	0,237	0,195	**0,023**
hsa_miR_524_3p	Pearson correlation coefficient (r)	-0,653	**0,963**	**0,882**	0,356	0,313	0,321	0,630
	Adjusted p-value	0,112	**0,000**	**0,009**	0,434	0,495	0,483	0,129
hsa_miR_524_5p	Pearson correlation coefficient (r)	-0,642	**0,950**	**0,717**	0,154	0,066	0,098	0,455
	Adjusted p-value	0,062	**0,000**	**0,030**	0,693	0,866	0,803	0,218
hsa_miR_525_3p	Pearson correlation coefficient (r)	**-0,768**	**0,817**	**0,779**	0,260	0,285	0,235	0,590
	Adjusted p-value	**0,016**	**0,007**	**0,013**	0,499	0,457	0,544	0,094

hsa_miR_525_5p	Pearson correlation coefficient (r)	-0,688	**0,719**	**0,841**	**0,776**	**0,808**	**0,776**	**0,905**
	Adjusted p-value	0,059	**0,044**	**0,009**	**0,023**	**0,015**	**0,024**	**0,002**
hsa_miR_526b	Pearson correlation coefficient (r)	-0,637	**0,759**	0,667	**0,697**	0,562	0,612	**0,823**
	Adjusted p-value	0,065	**0,018**	0,050	**0,037**	0,116	0,080	**0,006**
hsa_miR_526b#	Pearson correlation coefficient (r)	**-0,699**	**0,954**	**0,962**	0,442	0,416	0,423	**0,729**
	Adjusted p-value	**0,036**	**0,000**	**0,000**	0,234	0,265	0,257	**0,026**
hsa_miR_532_3p	Pearson correlation coefficient (r)	**0,887**	**-0,751**	**-0,851**	**-0,716**	**-0,717**	**-0,704**	**-0,892**
	Adjusted p-value	**0,001**	**0,020**	**0,004**	**0,030**	**0,030**	**0,034**	**0,001**
hsa_miR_532_5p	Pearson correlation coefficient (r)	**0,722**	**-0,812**	**-0,868**	**-0,694**	-0,663	-0,655	**-0,883**
	Adjusted p-value	**0,028**	**0,008**	**0,002**	**0,038**	0,051	0,055	**0,002**
hsa_miR_542_5p	Pearson correlation coefficient (r)	0,566	**-0,863**	-0,654	-0,463	-0,355	-0,382	**-0,672**
	Adjusted p-value	0,112	**0,003**	0,056	0,210	0,348	0,310	**0,047**
hsa_miR_543	Pearson correlation coefficient (r)	**0,738**	**-0,732**	**-0,732**	-0,426	-0,377	-0,398	-0,622
	Adjusted p-value	**0,023**	**0,025**	**0,025**	0,253	0,317	0,289	0,073
hsa_miR_545	Pearson correlation coefficient (r)	0,345	-0,490	-0,436	0,104	0,101	0,144	-0,210
	Adjusted p-value	0,363	0,181	0,241	0,791	0,797	0,712	0,588
hsa_miR_548o	Pearson correlation coefficient (r)	0,363	-0,069	-0,163	**-0,774**	**-0,767**	**-0,770**	-0,633
	Adjusted p-value	0,336	0,861	0,675	**0,014**	**0,016**	**0,015**	0,067
hsa_miR_550	Pearson correlation coefficient (r)	-0,759	**0,977**	**0,971**	0,541	0,543	0,543	0,763
	Adjusted p-value	0,080	**0,001**	**0,001**	0,268	0,266	0,265	0,078
hsa_miR_550#	Pearson correlation coefficient (r)	**-0,679**	**0,734**	0,566	0,567	0,445	0,479	**0,704**
	Adjusted p-value	**0,044**	**0,024**	0,112	0,111	0,230	0,192	**0,034**
hsa_miR_551a	Pearson correlation coefficient (r)	0,034	0,050	-0,262	**-0,819**	**-0,822**	**-0,825**	-0,673
	Adjusted p-value	0,936	0,907	0,531	**0,013**	**0,012**	**0,012**	0,067
hsa_miR_551b	Pearson correlation coefficient (r)	-0,518	0,496	**0,815**	**0,782**	**0,854**	**0,828**	**0,852**
	Adjusted p-value	0,189	0,211	**0,014**	**0,022**	**0,007**	**0,011**	**0,007**
hsa_miR_566	Pearson correlation coefficient (r)	-0,475	0,201	0,361	**0,869**	**0,880**	**0,879**	0,737
	Adjusted p-value	0,281	0,665	0,426	**0,011**	**0,009**	**0,009**	0,059
hsa_miR_574_3p	Pearson correlation coefficient (r)	**0,821**	**-0,926**	**-0,862**	**-0,668**	-0,593	-0,616	**-0,879**
	Adjusted p-value	**0,007**	**0,000**	**0,003**	**0,049**	0,093	0,077	**0,002**
hsa_miR_582_5p	Pearson correlation coefficient (r)	**0,750**	**-0,867**	**-0,861**	-0,489	-0,467	-0,463	**-0,753**
	Adjusted p-value	**0,020**	**0,002**	**0,003**	0,182	0,205	0,209	**0,019**
hsa_miR_584	Pearson correlation coefficient (r)	0,624	-0,342	-0,560	-0,650	-0,670	-0,645	-0,688

	Adjusted p-value	0,098	0,407	0,149	0,081	0,069	0,084	0,059
hsa_miR_590_5p	Pearson correlation coefficient (r)	0,243	-0,176	-0,103	0,180	0,190	0,236	-0,014
	Adjusted p-value	0,528	0,651	0,792	0,643	0,624	0,540	0,971
hsa_miR_597	Pearson correlation coefficient (r)	0,662	**-0,945**	**-0,927**	-0,530	-0,495	-0,500	**-0,795**
	Adjusted p-value	0,052	**0,000**	**0,000**	0,142	0,175	0,171	**0,010**
hsa_miR_598	Pearson correlation coefficient (r)	**0,805**	-0,650	-0,641	-0,187	-0,181	-0,155	-0,459
	Adjusted p-value	**0,009**	0,058	0,063	0,630	0,642	0,690	0,214
hsa_miR_605	Pearson correlation coefficient (r)	-0,511	0,436	0,469	0,165	0,282	0,206	0,331
	Adjusted p-value	0,160	0,241	0,203	0,672	0,462	0,595	0,384
hsa_miR_615_3p	Pearson correlation coefficient (r)	-0,458	-0,140	-0,224	0,409	0,406	0,388	0,282
	Adjusted p-value	0,301	0,765	0,630	0,362	0,366	0,389	0,540
hsa_miR_625#	Pearson correlation coefficient (r)	-0,362	0,327	0,593	**0,951**	**0,954**	**0,973**	**0,859**
	Adjusted p-value	0,338	0,390	0,092	**0,000**	**0,000**	**0,000**	**0,003**
hsa_miR_628_3p	Pearson correlation coefficient (r)	-0,664	0,519	0,386	**0,684**	0,578	0,590	**0,731**
	Adjusted p-value	0,051	0,152	0,305	**0,042**	0,103	0,095	**0,025**
hsa_miR_629	Pearson correlation coefficient (r)	-0,455	0,354	0,602	**0,844**	**0,834**	**0,847**	**0,838**
	Adjusted p-value	0,218	0,350	0,086	**0,004**	**0,005**	**0,004**	**0,005**
hsa_miR_643	Pearson correlation coefficient (r)	-0,529	0,509	0,536	**0,947**	**0,929**	**0,914**	**0,943**
	Adjusted p-value	0,177	0,198	0,171	**0,000**	**0,001**	**0,002**	**0,000**
hsa_miR_651	Pearson correlation coefficient (r)	0,204	-0,422	-0,368	-0,196	-0,195	-0,147	-0,384
	Adjusted p-value	0,598	0,258	0,329	0,612	0,614	0,706	0,308
hsa_miR_652	Pearson correlation coefficient (r)	0,609	**-0,894**	**-0,794**	-0,365	-0,315	-0,319	-0,639
	Adjusted p-value	0,082	**0,001**	**0,011**	0,334	0,409	0,403	0,064
hsa_miR_654_3p	Pearson correlation coefficient (r)	0,459	**-0,735**	-0,576	-0,215	-0,124	-0,159	-0,454
	Adjusted p-value	0,214	**0,024**	0,105	0,578	0,751	0,682	0,220
hsa_miR_654_5p	Pearson correlation coefficient (r)	0,408	-0,549	-0,381	-0,208	-0,107	-0,166	-0,340
	Adjusted p-value	0,275	0,126	0,312	0,592	0,784	0,670	0,370
hsa_miR_660	Pearson correlation coefficient (r)	**0,764**	**-0,905**	**-0,848**	-0,381	-0,338	-0,330	**-0,695**
	Adjusted p-value	**0,017**	**0,001**	**0,004**	0,312	0,373	0,385	**0,038**
hsa_miR_663	Pearson correlation coefficient (r)	0,250	0,185	0,163	0,337	0,205	0,294	0,279
	Adjusted p-value	0,551	0,661	0,700	0,415	0,627	0,480	0,504
hsa_miR_664	Pearson correlation coefficient (r)	**0,824**	**-0,683**	**-0,774**	-0,599	-0,566	-0,581	**-0,751**
	Adjusted p-value	**0,006**	**0,043**	**0,014**	0,088	0,112	0,101	**0,020**

hsa_miR_675#	Pearson correlation coefficient (r)	0,492	-0,409	-0,279	-0,127	-0,098	-0,065	-0,329
	Adjusted p-value	0,262	0,362	0,544	0,786	0,835	0,890	0,471
hsa_miR_7	Pearson correlation coefficient (r)	**-0,767**	**0,952**	**0,870**	0,360	0,331	0,332	**0,670**
	Adjusted p-value	**0,016**	**0,000**	**0,002**	0,341	0,384	0,382	**0,048**
hsa_miR_708	Pearson correlation coefficient (r)	0,619	-0,655	**-0,686**	-0,549	-0,503	-0,512	**-0,703**
	Adjusted p-value	0,075	0,055	**0,041**	0,126	0,168	0,158	**0,035**
hsa_miR_708#	Pearson correlation coefficient (r)	0,079	0,181	-0,016	-0,446	-0,447	-0,403	-0,434
	Adjusted p-value	0,899	0,771	0,980	0,451	0,450	0,501	0,465
hsa_miR_7_1#	Pearson correlation coefficient (r)	-0,563	**0,758**	**0,962**	0,442	0,470	0,454	**0,705**
	Adjusted p-value	0,114	**0,018**	**0,000**	0,233	0,201	0,220	**0,034**
hsa_miR_720	Pearson correlation coefficient (r)	-0,358	0,181	0,348	0,112	0,285	0,186	0,223
	Adjusted p-value	0,344	0,642	0,359	0,775	0,458	0,632	0,563
hsa_miR_744	Pearson correlation coefficient (r)	0,649	**-0,870**	**-0,784**	-0,494	-0,432	-0,442	**-0,749**
	Adjusted p-value	0,059	**0,002**	**0,012**	0,176	0,246	0,234	**0,020**
hsa_miR_744#	Pearson correlation coefficient (r)	0,429	-0,481	**-0,767**	-0,307	-0,356	-0,355	-0,525
	Adjusted p-value	0,336	0,275	**0,044**	0,503	0,433	0,435	0,226
hsa_miR_760	Pearson correlation coefficient (r)	0,237	0,196	0,208	-0,290	-0,232	-0,255	-0,133
	Adjusted p-value	0,609	0,673	0,654	0,528	0,617	0,580	0,777
hsa_miR_766	Pearson correlation coefficient (r)	**0,698**	**-0,706**	**-0,668**	-0,588	-0,537	-0,548	**-0,755**
	Adjusted p-value	**0,036**	**0,034**	**0,049**	0,096	0,136	0,126	**0,019**
hsa_miR_769_3p	Pearson correlation coefficient (r)	0,364	-0,207	0,071	0,259	0,340	0,308	0,152
	Adjusted p-value	0,375	0,623	0,867	0,536	0,410	0,458	0,719
hsa_miR_769_5p	Pearson correlation coefficient (r)	-0,506	0,502	0,654	**0,757**	**0,728**	**0,753**	**0,757**
	Adjusted p-value	0,165	0,168	0,056	**0,018**	**0,026**	**0,019**	**0,018**
hsa_miR_873	Pearson correlation coefficient (r)	0,460	-0,629	-0,530	-0,270	-0,246	-0,229	-0,464
	Adjusted p-value	0,213	0,069	0,143	0,482	0,524	0,553	0,209
hsa_miR_876_3p	Pearson correlation coefficient (r)	**0,819**	**-0,773**	-0,587	-0,065	-0,046	-0,013	-0,384
	Adjusted p-value	**0,013**	**0,024**	0,126	0,878	0,914	0,975	0,348
hsa_miR_876_5p	Pearson correlation coefficient (r)	**0,735**	**-0,871**	-0,688	-0,064	-0,018	-0,012	-0,402
	Adjusted p-value	**0,038**	**0,005**	0,059	0,881	0,967	0,977	0,323
hsa_miR_885_5p	Pearson correlation coefficient (r)	-0,615	0,567	**0,819**	**0,861**	**0,901**	**0,884**	**0,935**
	Adjusted p-value	0,078	0,111	**0,007**	**0,003**	**0,001**	**0,002**	**0,000**
hsa_miR_886_3p	Pearson correlation coefficient (r)	0,651	**-0,722**	**-0,803**	-0,487	-0,489	-0,490	-0,664

	Adjusted p-value	0,057	**0,028**	**0,009**	0,183	0,182	0,181	0,051
hsa_miR_886_5p	Pearson correlation coefficient (r)	0,086	0,046	0,159	0,025	0,152	0,083	0,089
	Adjusted p-value	0,825	0,907	0,683	0,950	0,697	0,831	0,820
hsa_miR_887	Pearson correlation coefficient (r)	0,159	-0,263	-0,128	-0,440	-0,382	-0,407	-0,417
	Adjusted p-value	0,707	0,528	0,763	0,275	0,350	0,316	0,304
hsa_miR_888	Pearson correlation coefficient (r)	-0,325	0,345	0,461	**0,973**	**0,984**	**0,991**	**0,846**
	Adjusted p-value	0,432	0,403	0,250	**0,000**	**0,000**	**0,000**	**0,008**
hsa_miR_891a	Pearson correlation coefficient (r)	**-0,772**	**0,819**	0,519	0,409	0,364	0,363	0,592
	Adjusted p-value	**0,042**	**0,024**	0,233	0,362	0,423	0,423	0,162
hsa_miR_9	Pearson correlation coefficient (r)	-0,541	0,416	0,694	**0,693**	**0,802**	**0,741**	**0,749**
	Adjusted p-value	0,133	0,265	0,038	**0,039**	**0,009**	**0,022**	**0,020**
hsa_miR_92a	Pearson correlation coefficient (r)	**0,688**	**-0,886**	**-0,864**	-0,532	-0,480	-0,499	**-0,773**
	Adjusted p-value	**0,041**	**0,001**	**0,003**	0,140	0,191	0,171	**0,015**
hsa_miR_92b	Pearson correlation coefficient (r)	-0,346	0,064	-0,098	0,174	0,176	0,170	0,087
	Adjusted p-value	0,361	0,869	0,802	0,655	0,650	0,662	0,823
hsa_miR_92b#	Pearson correlation coefficient (r)	-0,127	0,433	0,418	0,186	0,102	0,153	0,264
	Adjusted p-value	0,744	0,244	0,262	0,631	0,793	0,694	0,493
hsa_miR_93	Pearson correlation coefficient (r)	-0,563	0,559	**0,767**	0,535	0,628	0,577	**0,704**
	Adjusted p-value	0,114	0,118	**0,016**	0,138	0,070	0,104	**0,034**
hsa_miR_93#	Pearson correlation coefficient (r)	-0,361	0,448	0,451	-0,033	-0,009	-0,017	0,201
	Adjusted p-value	0,340	0,227	0,224	0,933	0,982	0,964	0,605
hsa_miR_940	Pearson correlation coefficient (r)	0,597	-0,132	-0,084	0,085	0,064	0,112	-0,040
	Adjusted p-value	0,118	0,756	0,842	0,841	0,880	0,791	0,925
hsa_miR_941	Pearson correlation coefficient (r)	0,651	-0,530	-0,380	-0,390	-0,327	-0,347	-0,497
	Adjusted p-value	0,058	0,142	0,313	0,299	0,391	0,360	0,174
hsa_miR_95	Pearson correlation coefficient (r)	-0,065	-0,187	0,164	0,100	0,291	0,203	0,079
	Adjusted p-value	0,869	0,630	0,674	0,798	0,447	0,601	0,839
hsa_miR_96	Pearson correlation coefficient (r)	-0,515	0,390	0,667	**0,888**	**0,956**	**0,926**	**0,871**
	Adjusted p-value	0,156	0,299	0,050	**0,001**	**0,000**	**0,000**	**0,002**
hsa_miR_98	Pearson correlation coefficient (r)	**0,774**	**-0,788**	**-0,781**	-0,556	-0,486	-0,506	**-0,770**
	Adjusted p-value	**0,014**	**0,012**	**0,013**	0,120	0,185	0,165	**0,015**
hsa_miR_99a	Pearson correlation coefficient (r)	0,658	**-0,904**	**-0,832**	-0,590	-0,498	-0,529	**-0,807**
	Adjusted p-value	0,054	**0,001**	**0,005**	0,094	0,172	0,143	**0,008**

hsa_miR_99a#	Pearson correlation coefficient (r)	0,595	**-0,822**	**-0,746**	**-0,716**	-0,605	-0,648	**-0,859**
	Adjusted p-value	0,091	**0,007**	**0,021**	**0,030**	0,084	0,059	**0,003**
hsa_miR_99b	Pearson correlation coefficient (r)	**0,743**	**-0,826**	**-0,847**	-0,646	-0,596	-0,611	**-0,845**
	Adjusted p-value	**0,022**	**0,006**	**0,004**	0,060	0,090	0,081	**0,004**
hsa_miR_99b#	Pearson correlation coefficient (r)	**0,692**	**-0,679**	**-0,885**	**-0,722**	**-0,744**	**-0,737**	**-0,867**
	Adjusted p-value	**0,039**	**0,044**	**0,002**	**0,028**	**0,021**	**0,024**	**0,002**
hsa_miR_505#	Pearson correlation coefficient (r)	0,455	-0,685	**-0,908**	**-0,912**	**-0,877**	**-0,882**	**-0,938**
	Adjusted p-value	0,365	0,133	**0,012**	**0,011**	**0,022**	**0,020**	**0,006**
hsa_miR_629#	Pearson correlation coefficient (r)	-0,496	**0,839**	**0,858**	0,176	0,161	0,146	0,498
	Adjusted p-value	0,317	**0,037**	**0,029**	0,739	0,761	0,783	0,314
hsa_miR_661	Pearson correlation coefficient (r)	-0,761	0,686	0,556	0,719	0,687	0,649	**0,825**
	Adjusted p-value	0,079	0,133	0,252	0,107	0,132	0,163	**0,043**
hsa_miR_649	Pearson correlation coefficient (r)	**-0,819**	**0,887**	**0,837**	0,687	0,617	0,589	**0,851**
	Adjusted p-value	**0,046**	**0,019**	**0,038**	0,132	0,192	0,219	**0,032**
hsa_miR_20b#	Pearson correlation coefficient (r)	0,865	**-0,989**	**-0,879**	-0,478	-0,414	-0,438	-0,731
	Adjusted p-value	0,058	**0,001**	**0,049**	0,415	0,488	0,461	0,160
hsa_miR_767_5p	Pearson correlation coefficient (r)	-0,455	0,762	0,656	0,519	0,432	0,495	0,548
	Adjusted p-value	0,364	0,078	0,157	0,292	0,393	0,318	0,260
hsa_miR_548b_3p	Pearson correlation coefficient (r)	-0,583	0,537	0,738	0,723	0,847	0,799	0,750
	Adjusted p-value	0,225	0,272	0,094	0,105	0,033	0,057	0,086
hsa_miR_675b	Pearson correlation coefficient (r)	0,492	-0,409	-0,279	-0,127	-0,098	-0,065	-0,329
	Adjusted p-value	0,262	0,362	0,544	0,786	0,835	0,890	0,471
hsa_miR_338_5p	Pearson correlation coefficient (r)	**-0,846**	0,647	0,425	0,188	0,108	0,078	0,405
	Adjusted p-value	**0,034**	0,165	0,401	0,721	0,838	0,883	0,426
hsa_miRPlus_C1089	Pearson correlation coefficient (r)	0,396	-0,412	**-0,743**	-0,320	-0,381	-0,371	-0,484
	Adjusted p-value	0,292	0,271	**0,022**	0,401	0,311	0,326	0,187
hsa_miRPlus_D1033	Pearson correlation coefficient (r)	-0,113	-0,058	-0,085	0,527	0,535	0,506	0,392
	Adjusted p-value	0,773	0,881	0,827	0,144	0,137	0,165	0,296
SNORD38B	Pearson correlation coefficient (r)	0,177	-0,158	-0,072	-0,454	-0,325	-0,385	-0,387
	Adjusted p-value	0,649	0,684	0,854	0,219	0,394	0,306	0,303
SNORD38B_dup	Pearson correlation coefficient (r)	-0,051	-0,073	-0,034	-0,291	-0,175	-0,230	-0,254
	Adjusted p-value	0,897	0,851	0,930	0,447	0,652	0,552	0,510
SNORD49A	Pearson correlation coefficient (r)	0,129	-0,292	-0,256	-0,429	-0,286	-0,361	-0,435

	Adjusted p-value	0,740	0,445	0,507	0,250	0,455	0,340	0,242
SNORD49A_dup	Pearson correlation coefficient (r)	0,220	-0,432	-0,265	-0,292	-0,114	-0,200	-0,362
	Adjusted p-value	0,569	0,246	0,490	0,446	0,770	0,605	0,338
U6	Pearson correlation coefficient (r)	0,030	0,045	-0,040	-0,455	-0,388	-0,423	-0,371
	Adjusted p-value	0,939	0,908	0,919	0,219	0,302	0,257	0,326
U6_dup	Pearson correlation coefficient (r)	-0,322	0,024	-0,041	-0,045	0,049	-0,053	0,036
	Adjusted p-value	0,398	0,950	0,916	0,908	0,900	0,892	0,926

Supplementary Table S3. List of the 50 stables miRNAs selected to normalize.

miRNAs	CS1 *Cp* value	CS2 *Cp* value	CS3 *Cp* value	SpF-scMF1 *Cp* value	SpF-scMF2 *Cp* value	SpF-scMF3 *Cp* value	SCO1 *Cp* value	SCO2 *Cp* value	SCO3 *Cp* value	mean (*Cp* value)	SD (*Cp* value)	CV (*Cp* value)
average 50 stables miRNAs	31,65	31,83	31,68	32,07	31,66	31,86	31,72	31,28	31,38	31,68	0,24	0,0075
hsa-let-7d*	25,08	25,49	25,63	25,03	25,21	25,77	25,21	25,42	25,27	25,34	0,25	0,0099
hsa-miR-1296	31,95	31,60	31,99	32,27	31,65	31,78	32,61	32,27	31,99	32,01	0,33	0,0102
hsa-miR-149*	34,07	33,84	33,96	34,18	33,65	34,91	34,46	34,10	34,11	34,14	0,36	0,0107
hsa-miR-628-3p	34,44	33,90	34,67	34,73	34,93	35,21	34,74	34,75	35,03	34,71	0,38	0,0108
hsa-miR-340	36,03	36,24	36,99	36,87	36,47	35,88	36,89	36,20	36,20	36,42	0,41	0,0111
hsa-miR-16	26,49	26,81	26,20	27,27	26,65	26,49	26,94	26,64	26,60	26,68	0,30	0,0114
hsa-miR-30e*	31,00	31,30	30,87	31,82	31,21	31,71	31,63	30,78	31,15	31,27	0,37	0,0120
hsa-miR-25	31,57	31,57	31,27	32,44	31,60	31,74	31,72	31,06	31,43	31,60	0,38	0,0121
hsa-miR-1201	31,73	32,00	31,65	32,16	31,52	32,66	32,27	31,62	31,59	31,91	0,39	0,0121
hsa-miR-92b	32,62	33,16	32,77	32,74	33,35	33,23	33,29	32,04	32,72	32,88	0,42	0,0128
hsa-miR-342-3p	30,63	30,73	30,44	30,36	30,35	29,99	30,35	29,64	29,67	30,24	0,39	0,0129
hsa-miR-93	29,79	29,81	29,42	30,49	29,93	29,65	30,65	29,94	29,84	29,95	0,39	0,0129
hsa-miR-188-5p	35,02	34,80	34,93	34,30	35,23	34,87	34,53	34,11	33,90	34,63	0,45	0,0130
hsa-miR-20a	27,22	27,67	27,09	27,94	27,10	27,51	27,01	26,97	26,92	27,27	0,36	0,0130
hsa-miR-132	30,84	30,92	30,79	31,42	30,95	30,92	31,05	29,91	30,66	30,83	0,40	0,0131
hsa-miR-215	33,57	34,14	33,82	34,43	34,14	34,16	35,12	34,33	34,68	34,27	0,45	0,0133
hsa-miR-95	34,35	34,62	33,44	34,85	34,00	34,25	33,69	33,75	34,13	34,12	0,45	0,0133
hsa-miR-605	31,32	31,59	30,31	30,91	31,15	30,81	31,02	31,55	31,46	31,13	0,42	0,0134
hsa-miR-193a-5p	36,00	36,05	35,81	36,24	35,95	35,80	35,72	34,79	35,05	35,71	0,48	0,0134
hsa-miR-301a	32,24	32,71	32,61	33,43	32,63	33,02	32,47	31,86	32,54	32,61	0,45	0,0137
hsa-let-7a-2*	35,58	35,57	36,51	35,81	35,12	35,64	34,94	35,21	36,11	35,61	0,49	0,0138
hsa-miR-30c	27,48	27,61	27,45	27,73	27,43	27,59	27,15	26,60	26,84	27,32	0,38	0,0139
hsa-miR-1248	31,66	32,06	32,70	31,94	31,83	31,57	31,98	32,66	31,42	31,98	0,45	0,0140
hsa-miR-93*	33,88	33,75	33,87	34,55	33,74	32,94	33,68	34,47	34,15	33,89	0,48	0,0141
hsa-miR-550*	34,82	34,56	35,46	35,18	35,15	35,58	35,25	35,94	36,12	35,34	0,50	0,0141
hsa-miR-483-3p	34,76	34,89	34,67	34,25	34,96	35,08	34,93	33,77	33,86	34,58	0,49	0,0142
hsa-miR-378	32,24	32,28	32,84	33,01	32,89	32,78	33,07	31,67	32,33	32,57	0,47	0,0143
hsa-miR-484	31,27	31,07	30,61	31,19	31,10	30,94	31,21	29,92	30,54	30,87	0,44	0,0143
hsa-miR-106b	33,76	34,23	33,84	34,96	34,12	33,70	34,21	33,49	33,32	33,96	0,49	0,0144
hsa-miR-296-5p	31,74	31,51	31,85	32,81	31,85	32,15	32,76	32,59	32,12	32,15	0,47	0,0146
hsa-miR-30e	32,96	32,47	32,23	32,75	31,79	32,71	32,26	31,49	32,01	32,30	0,48	0,0149
hsa-miR-101*	35,98	35,62	35,54	35,75	35,74	35,84	35,92	34,48	34,76	35,51	0,53	0,0149

hsa-miR-150	31,36	30,79	31,09	30,66	30,49	31,13	31,05	29,79	30,86	30,80	0,46	0,0150
hsa-let-7b*	31,24	31,70	31,67	31,58	31,21	31,28	30,97	30,49	30,43	31,17	0,47	0,0151
hsa-miR-18a*	32,99	33,44	33,88	33,52	33,01	34,19	33,33	34,09	32,67	33,46	0,52	0,0155
hsa-miR-335	32,10	32,66	31,58	32,71	31,79	31,91	31,69	31,70	31,17	31,92	0,50	0,0157
hsa-miR-15b	28,02	28,11	27,92	28,91	28,10	29,11	28,82	28,17	28,13	28,36	0,45	0,0158
hsa-miR-191	30,02	30,29	30,09	30,92	30,07	30,52	29,73	29,59	29,34	30,06	0,48	0,0160
hsa-miR-501-5p	32,12	32,80	32,28	31,94	32,55	32,28	31,31	31,80	31,29	32,04	0,51	0,0160
hsa-miR-425	31,28	31,77	31,47	32,52	31,43	31,13	31,20	31,00	30,80	31,40	0,51	0,0161
hsa-miR-191	30,15	30,44	30,21	30,98	29,94	30,55	29,82	29,46	29,53	30,12	0,49	0,0163
hsa-miR-126*	31,31	30,90	30,32	31,17	30,80	30,93	31,24	29,70	30,60	30,77	0,51	0,0166
hsa-miR-106a	28,13	28,72	28,19	28,82	28,02	28,70	27,75	27,63	27,66	28,18	0,47	0,0167
hsa-miR-590-5p	31,73	33,18	32,49	32,79	32,69	32,61	32,87	31,67	31,77	32,42	0,56	0,0172
hsa-miR-210	31,71	31,73	31,86	31,50	31,09	31,52	30,61	30,55	30,56	31,24	0,54	0,0173
hsa-miR-152	30,10	30,20	29,65	30,86	30,08	30,13	29,98	29,03	29,43	29,94	0,52	0,0174
hsa-miR-190	32,86	32,72	32,90	33,69	32,84	33,28	32,33	31,90	32,00	32,72	0,58	0,0176
hsa-miR-20a*	33,10	33,45	32,90	33,24	32,54	32,98	32,03	32,14	31,84	32,69	0,58	0,0176
hsa-miR-1974	21,12	22,17	21,70	22,00	21,56	22,19	21,48	21,27	21,49	21,66	0,38	0,0177
hsa-miR-30d	31,22	31,93	31,71	31,79	31,48	31,53	31,04	30,21	30,76	31,30	0,55	0,0177

Supplemental Table S4. List of selected miRNAs analysed by RT-qPCR

miRNA	miRBase v18	Sequence	Assay no.	Sample type
hsa-miR-34c	hsa-miR-34c-5p	AGGCAGUGUAGUUAGCUGAUUGC	204407; 205659	Sperm; Testis
hsa-miR-320a	hsa-miR-320a	AAAAGCUGGGUUGAGAGGGCGA	204154	Sperm
hsa-let-7b	hsa-let-7b-5p	UGAGGUAGUAGGUUGUGUGGUU	204750	Sperm
hsa-miR-22	hsa-miR-22-3p	AAGCUGCCAGUUGAAGAACUGU	204606	Sperm
hsa-miR-122	hsa-miR-122-5p	UGGAGUGUGACAAUGGUGUUUG	204090; 205664	Sperm; Testis
hsa-miR-423	hsa-miR-423-3p	AGCUCGGUCUGAGGCCCCUCAGU	204488	Sperm
	hsa-miR-423-5p	UGAGGGGCAGAGAGCGAGACUUU	204593	Sperm
hsa-miR-375	hsa-miR-375	UUUGUUCGUUCGGCUCGCGUGA	204362	Sperm
hsa-let-7c	hsa-let-7c-5p	UGAGGUAGUAGGUUGUAUGGUU	204444	Sperm
hsa-miR-140	hsa-miR-140-3p	UACCACAGGGUAGAACCACGG	204304	Sperm
hsa-miR-21	hsa-miR-21-5p	UAGCUUAUCAGACUGAUGUUGA	204230	Sperm
hsa-miR-152	hsa-miR-152-3p	UCAGUGCAUGACAGAACUUGG	204294	Sperm
hsa-miR-30a	hsa-miR-30a-5p	UGUAAACAUCCUCGACUGGAAG	204039	Sperm
hsa-miR-148a	hsa-miR-148a-3p	UCAGUGCACUACAGAACUUUGU	204121	Sperm
hsa-let-7g	hsa-let-7g-5p	UGAGGUAGUAGUUUGUACAGUU	204565	Sperm
hsa-miR-192	hsa-miR-192-5p	CUGACCUAUGAAUUGACAGCC	204099	Sperm
hsa-miR-10a	hsa-miR-10a-5p	UACCCUGUAGAUCCGAAUUUGUG	204600	Sperm
hsa-miR-335	hsa-miR-335-5p	UCAAGAGCAAUAACGAAAAAUGU	204151	Sperm
hsa-miR-191	hsa-miR-191-5p	CAACGGAAUCCCAAAAGCAGCUG	204306	Sperm
hsa-miR-25	hsa-miR-25-3p	CAUUGCACUUGUCUCGGUCUGA	204361	Sperm
hsa-miR-34b	hsa-miR-34b-3p	CAAUCACUAACUCCACUGCCAU	204005	Sperm
hsa-miR-221	hsa-miR-221-3p	AGCUACAUUGUCUGCUGGGUUUC	204532	Sperm
hsa-miR-449a	hsa-miR-449a	UGGCAGUGUAUUGUUAGCUGGU	204481	Sperm; Testis
hsa-miR-30e-3p	hsa-miR-30e-3p	CUUUCAGUCGGAUGUUUACAGC	204410	Testis
